# Correlations Between the Metabolome and the Endophytic Fungal Metagenome Suggests Importance of Various Metabolite Classes in Community Assembly in Horseradish (*Armoracia rusticana*, Brassicaceae) Roots

**DOI:** 10.3389/fpls.2022.921008

**Published:** 2022-06-17

**Authors:** Tamás Plaszkó, Zsolt Szűcs, Zoltán Cziáky, Lajos Ács-Szabó, Hajnalka Csoma, László Géczi, Gábor Vasas, Sándor Gonda

**Affiliations:** ^1^Department of Botany, Faculty of Science and Technology, University of Debrecen, Debrecen, Hungary; ^2^Doctoral School of Pharmaceutical Sciences, University of Debrecen, Debrecen, Hungary; ^3^Agricultural and Molecular Research and Service Institute, University of Nyíregyháza, Nyíregyháza, Hungary; ^4^Department of Genetics and Applied Microbiology, Faculty of Science and Technology, University of Debrecen, Debrecen, Hungary

**Keywords:** plant holobiont, fungal endophytes, glucosinolate decomposition, flavonoid glycosides, tryptophan derivatives

## Abstract

The plant microbiome is an increasingly intensive research area, with significance in agriculture, general plant health, and production of bioactive natural products. Correlations between the fungal endophytic communities and plant chemistry can provide insight into these interactions, and suggest key contributors on both the chemical and fungal side. In this study, roots of various horseradish (*Armoracia rusticana*) accessions grown under the same conditions were sampled in two consecutive years and chemically characterized using a quality controlled, untargeted metabolomics approach by LC-ESI-MS/MS. Sinigrin, gluconasturtiin, glucoiberin, and glucobrassicin were also quantified. Thereafter, a subset of roots from eight accessions (*n* = 64) with considerable chemical variability was assessed for their endophytic fungal community, using an ITS2 amplicon-based metagenomic approach using a custom primer with high coverage on fungi, but no amplification of host internal transcribed spacer (ITS). A set of 335 chemical features, including putatively identified flavonoids, phospholipids, peptides, amino acid derivatives, indolic phytoalexins, a glucosinolate, and a glucosinolate downstream product was detected. Major taxa in horseradish roots belonged to Cantharellales, Glomerellales, Hypocreales, Pleosporales, Saccharomycetales, and Sordariales. Most abundant genera included typical endophytes such as *Plectosphaerella, Thanatephorus, Podospora, Monosporascus, Exophiala*, and *Setophoma*. A surprising dominance of single taxa was observed for many samples. In summary, 35.23% of reads of the plant endophytic fungal microbiome correlated with changes in the plant metabolome. While the concentration of flavonoid kaempferol glycosides positively correlated with the abundance of many fungal strains, many compounds showed negative correlations with fungi including indolic phytoalexins, a putative glucosinolate but not major glucosinolates and a glutathione isothiocyanate adduct. The latter is likely an *in vivo* glucosinolate decomposition product important in fungal arrest. Our results show the potency of the untargeted metabolomics approach in deciphering plant–microbe interactions and depicts a complex array of various metabolite classes in shaping the endophytic fungal community.

## Introduction

It was recognized long ago that organic compounds of plant metabolism serve as nutrients for microorganisms and that plants defend themselves against pathogenic microbes by producing antimicrobial specialized products. Recent advancements show that this situation is much more complex *in situ* (Pang et al., 2021). Namely, evolutionary selection results in the building of flexible meta-organisms in which the plant actively feeds its microbiome (the consortia of various microbes associated with the plant) and shapes its composition by exudates to prevent the assembly of a pathogenic microbial community, while the microbiome members strive to take advantage of the plant compounds and nutrients in strong competition with other candidates and defend against plant antimicrobials at the same time (Sasse et al., 2018; Pang et al., 2021). This results in a dynamically changing, interacting plant metabolome and plant microbiome. This interaction was shown to occur with both primary (e.g., the polysaccharide mucilages and various organic acids) and specialized plant metabolites concerning the rhizoplane microbiome. The border cells with more active specialized and less active primary metabolism suggest the importance of specialized metabolites (Sasse et al., 2018), but data on the exact proportion of the contribution of various compounds are limited. While most of the scientific data is still exclusively gathered on bacterial interactions, there is some evidence about the same phenomena in the fungal kingdom.

The interaction between endophytic fungi and the plant metabolome can take the form of one of two main effects. First, a fungal challenge typically results in elicitation—the induction of biosynthesis of specialized metabolites. Several studies of this approach focus on specialized metabolite production in tissue cultures, which is triggered to increase after mimicking fungal challenges with fungal cell wall extracts (Narayani and Srivastava, 2017). In addition, various models of plant pathogenesis also showed induction of defensive components: lots of studies have reported increased production of various subclasses of glucosinolates (GSLs) in Brassicaceae plants after fungal challenge (Ishimoto et al., 2004; Robin et al., 2017; Madloo et al., 2019). The results of such studies are excellent sources of information on key pathogens of interest and possible defense mechanisms but cannot be extrapolated to situations when the plants are challenged by a mixture of fungal strains: even dual combinations of fungi can have dramatically different effects on plants (Hori et al., 2021). One also cannot estimate the relative importance of metabolite classes with studies focusing on a single compound class.

The chemical complexity of the same issue is well exemplified by an NMR metabolomics study which showed an increase in concentrations of various compound classes, including phenylpropanoids, flavonoids and GSLs after fungal challenge (Abdel-Farid et al., 2009). What is more, most studies focus on the specialized metabolites of plants, but this does not mean that they are the sole contributors to defense. For example, in a ^1^H-NMR study on *Combretum lanceolatum* (Combretaceae), colonization by an endophytic fungus significantly changed the primary metabolism of the plant. Induced compounds included threonine, malic acid, and N-acetyl-mannosamine, fold-changes were in the 290- to 740-fold range (Lacerda et al., 2021). The authors concluded that these compounds might serve as precursors of special metabolites involved in plant self-defense.

Challenges and colonization by non-pathogenic endophytic fungi also frequently result in an increase in the biosynthesis of specialized metabolites in plant tissue cultures and plants under field conditions as well. Examples include labdane diterpenes of *Coleus forskohlii* after colonization with *Fusarium redolens*, *Phialemoniopsis cornearis*, and *Macrophomina pseudophaseolina* (Mastan et al., 2019). The same plant can produce different specialized metabolites in different locations, some of which are due to the microbes related to different sites of residence. Microorganisms that adapt to specific locations and are associated with certain plants may have unique effects on host plants, like specialized metabolism (Pang et al., 2021).

Studies of another approach reported the selection and recruitment of various microbes by plants using metabolites. Results of these studies suggest that specialized metabolites are the significant factors driving microbes toward plants, as microbes can recognize their hosts via root exudates, e.g., strigolactones and flavonoids serve as signals for arbuscular mycorrhizal symbioses (Holmer et al., 2017). The importance of specialized metabolites is also apparent in the microbial composition of the host plant: studies on bacteria and fungi have shown that alterations in communities can be induced by several compound classes, including benzoxazinoids (Hu et al., 2018), coumarins (Voges et al., 2019), flavonoids (Szoboszlay et al., 2016), triterpenes (Huang et al., 2019) or GSL decomposition products (Plaszkó et al., 2021). However, data on complete plant metabolomes (which enable studying rank of contribution strength) are quite limited.

The amount of data on communities of endophytic fungi is also limited compared to that on soil and rhizosphere fungi or endophytic bacteria, though endophytic fungi are ubiquitous in plants (Rodriguez et al., 2009), they also likely show functions of well-studied mycorrhizae in non-mycorrhizal species (Hiruma et al., 2018), such as most Brassicaceae (Pongrac et al., 2013). Root endophytic communities are logically assembled from the highly variable soil communities. Although the rhizosphere is rich in carbon sources compared to bulk soil, only a low percentage of the soil microbes are abundant around plants (DeAngelis et al., 2009) and only a subset of these fungi have the ability to colonize the plants’ endosphere (Sasse et al., 2018). This phenomenon indicates that microbes have to cope with a different chemical milieu around the rhizosphere, which leads to stronger selection pressure. It seems that endophytic fungi are more likely to develop mechanisms to neutralize or tolerate the specialized metabolome of the host plant (Szűcs et al., 2018).

Data on the assembly of the endophytic community as a function of plant chemistry are warranted. As most Brassicaceae plants have no mycorrhizae (Pongrac et al., 2013), they are good candidates to study the assembly of the endophytic community. The very high amount of GSLs in horseradish (*Armoracia rusticana*) (Agneta et al., 2014; Gonda et al., 2016) makes it an excellent model to study correlations between abundances of chemical and endophytic features.

The aim of the current study was to assess the interaction between the fungal endophyte composition and the metabolome, using horseradish as a model plant. To obtain these data, a variety of different horseradish accessions was raised under identical conditions for two consecutive years to generate a chemically variable set of roots from the same soil.

## Materials and Methods

### Horseradish Accessions Cultivation and Sampling

Different horseradish (*A. rusticana* G.Gaertn., B.Mey. & Scherb.) accessions were grown at an agricultural site in Hungary (Site 1, 47°39′09.8″N 21°42′30.5″E) for maintenance of cultivars, with the same agricultural methodology described in a previous publication (Papp et al., 2018). Samples from 13 different accessions (*n* ≥ 4 for each accession each year) and bulk soil samples (*n* = 4 each year) were collected in November 2018 and 2019. Meteorological data obtained by interpolation of three official weather stations’ data, nearest to this site can be found in Supplementary Table 1. As controls, carrots (*Daucus carota* L.) from the same site and additional horseradish roots from a different site with different soil types (Site 2, 47°32′02.7″N 21°49′42.6″E) were also collected in November of 2019. The leaves of the carrot and horseradish plants were removed and the roots were placed in sterile plastic bags. The roots were transferred to our department for further processing. The roots were washed with tap water to remove any soil residues, then immersed for 30 s in 96% ethanol. Subsequently, the roots were immersed for 10 min in NaOCl solution containing 2.5% active chlorine and 0.1% Tween 20. The exact NaOCl concentration was determined by titrimetry. After the surface sterilization, the samples were washed with autoclaved type II water 5 times in sterile plastic bags. The sterility of the washing fluids were tested by a similar methodology described in our previous publication (Szűcs et al., 2018). A representative vertical piece (∼1/2 to 1/16 part, depending on root size) of the roots was cut out and cut into pieces approximately 1 × 1 × 1 cm in size, with a sterile steel knife under a laminar airflow. The samples (∼10 g) were put in aseptic plastic specimen jars, frozen in liquid nitrogen and stored in −80°C until further processing. The bulk soil samples were collected in specimen jars, frozen in liquid N_2_ and stored in −80°C.

### Sample Preparation and Cryogenic Grinding

The frozen plant materials were loaded into autoclaved 50 mL stainless steel grinding jars (Retsch GmbH, Haan, Germany) pre-cooled in liquid N_2_. The loaded and assembled jars were immersed in LN_2_ again to avoid thawing of the plant material. Samples were homogenized in a Retsch Mixer Mill MM 400, at 30 s^–1^ frequency for 45–90 s using a 20 mm stainless steel ball. Homogenized samples were lyophilized prior to genomic DNA and metabolome extractions and kept in exsiccators over silica adsorbent at room temperature.

### Phytochemical Analysis

A total of 25 mg of the lyophilized horseradish material was accurately weighed and extracted at 4°C for 5 min with 1 mL 75% aqueous methanol supplemented with 0.1% formic acid, in a vortex disruptor. In preliminary examinations, this extractant showed stability of the extract for 48 h, as well as high metabolite coverage. Following centrifugation at 4°C, 24,000 *g* for 3 min, extracts were diluted 10- or 200-fold with the extraction solvent for untargeted metabolomics and quantification of GSLs, respectively. The extracts were filtered (PTFE Syringe Filter, 0.22 μm pore size, Filter-Bio, Nantong, China) before instrumental analysis. From accessions that had ≥4 replicates for both years (*n* = 13), 10 accessions (*n* = 10 × 2 × 4) were selected based on preliminary chemical analysis and subjected to untargeted metabolomics.

#### Instrumentation and LC-MS Measuring Parameters

For LC-MS measurements, a UHPLC system (Dionex Ultimate 3000RS) coupled to a Thermo Q Exactive Orbitrap mass spectrometer (Thermo Fisher Scientific Inc., Waltham, MA, United States) with an electrospray ionization source (ESI) was used.

For untargeted metabolomics, the separation column was a Kinetex Polar C_18_ 100 × 3 mm × 2.6 μm, 100 Å column; thermostated to operate at 25°C. A gradient elution method was used, using mixtures of solvent A (water + 0.1% formic acid) and solvent B (MeCN + 0.1% formic acid) at a flow rate of 0.2 mL min^–1^, as follows: 0–2 min, 0% B; 2–14 min, 0–100% B; 14–15 min, 100% B; 15–16 min, 100–0% B; and 16–25 min, 0% B. From all samples, 1 μL was injected. The Orbitrap was operated in full MS mode at m/z range 125–800 and FWHM resolution 35,000, with polarity switching enabled during all quantitative measurements. The capillary temperature was 320°C, the maximum injection time was 100 ms, and sheath gas and aux flow rates were 32 and 7 arb, respectively. Spray voltage was 3.8 and 4.0 kV for negative and positive ion modes, respectively. For quantitative evaluation of the main GSLs (sinigrin, gluconasturtiin, glucoiberin, and glucobrassicin), the method described in (Szűcs et al., 2018) was used without modifications, using the same instrument.

#### MS/MS of Quality Control-Passed Features

Targeted fragmentation of all features of interest was accomplished with similar parameters, except that the mass range was reduced to include the ions of the inclusion list only and that positive and negative ion mode data were separately recorded. The list of candidate features was prepared from features that passed metabolomics quality control (QC) (see section “False Discovery Rate Adjustments”). The list of candidates was split into inclusion lists so that at most 5 co-eluting features were included in a single list, resulting in good coverage of all list members around their peak tops. Depending on the inclusion list overlaps, the top 2–5 features were selected for fragmentation at 30 normalized collision energy (NCE) in a rotation scheme (loop count = 5, topN = 5). The maximum ion collection time was set at 250 ms.

#### Peak Detection

Raw instrument files were converted into mzXML and processed using XCMSOnline 2.7.2 (XCMS 1.47.3) (Gowda et al., 2014). The feature detection method was developed from the default settings suggested for Orbitrap default, with some modifications. The detailed list of parameters is available as a supplementary in Supplementary Table 2. For quantitative evaluation of the main GSLs, four-point calibration curves were used for all GSLs to be in the linear range of calibration (Szűcs et al., 2018). In this case, a targeted peak detection was conducted in mzMine 2.53 (Pluskal et al., 2010). Parameters are available in Supplementary Table 3.

#### Metabolomics Quality Control, Performance Assessment, and Adjustments

##### Quality Control Samples for Untargeted Metabolomics

Metabolomics QC samples were prepared according to the “long-term reference samples” approach (Dudzik et al., 2018; Evans et al., 2020) by mixing equal volumes of samples from each treatment with the expectation to cover the chemical variability. This meant mixing a concentrated sample from each accession from Site 1 in 2018 (*n* = 23) and a concentrated sample from the roots of all sampling points of Site 2 in 2019 (*n* = 13). A 50 mL QC mixture was separated into aliquots and stored under LN_2_ until use. For each sequence, 2 mL of QC sample was allowed to thaw and used to generate the QC sample set for the measurement sequence by 10-fold dilution.

##### Run Order Randomization and Measurement Sequence Design

After a thorough wash, the run LC-MS sequences began with a solvent blank (Evans et al., 2020), followed by two samples of QC samples, a 3-point QC linearity sample set (25-, 10-, and 5-fold dilutions), a four-to-six sample set of QC samples and blocks of real samples with QCs in every sixth or seventh injection. Real samples were injected in a randomized order for all applications (Evans et al., 2020). The leading QC block enables equilibration with the column presumably by equilibrating with active sites, stabilizes retention times, and hence prevents the need to discard the first few injections (Dudzik et al., 2018).

##### Feature Acceptance Criteria

After discarding isotope and adduct features, the integrated values from QCs were used to filter features so that only those are kept that could be measured with sufficient linearity and precision. Features with a relative standard deviation (SD/mean) above the cut-off threshold of 30% were discarded from further analysis (Dunn et al., 2011). Later, the data were also corrected for minor deviations in sensitivity by a curve-fitting adjustment as the final step, as explained in section “Adjustments to Sensitivity Drifts.” An additional, less widely applied filter was also utilized to ensure a strong linear relationship between signals and the concentrations for the examined features. This was done by evaluating the linearity of the response between the concentration (the inverse of the dilution) and the abundance by Pearson correlation for all features that passed the former RSD filter. For this purpose, a QC linearity sample set (serial dilution) and a blank were used. This approach keeps only those features that respond linearly within the range of dilution (Broadhurst et al., 2018). The most concentrated QC sample is twofold more concentrated than that of the real samples. Only features with *R*^2^ > 0.8 were kept for further analysis. Note that this step also discards features that are present in significant amounts in the blank sample.

##### Adjustments to Sensitivity Drifts

Finally, a low-order non-linear locally estimated smoothing (LOESS) function was fitted to data from QCs for all features separately, according to Dunn et al. (2011). The assumed theoretical sensitivities between known values (QCs) were calculated by the fitted curve for each metabolite separately, and the feature intensities of real samples were corrected with these values. Essentially, this expresses all abundances as fold-changes where the reference (1.00) is the feature abundance in a pooled QC sample, enabling the merge of sequences measured months apart. The amount of extracted dry weight was corrected after this step.

#### Identification and Putative Compound Class Annotation of Unknown Compounds

MS^2^ spectra were harvested from raw measurement files with the R package CluMSID (Depke et al., 2019). For each feature, the 10 most abundant MS^2^ spectra were used to generate a consensus spectrum, which was exported from R and subsequently imported into SIRIUS 4.9.9 (Dührkop et al., 2019) for annotation. The CSI:FingerID and CANOPUS algorithms of SIRIUS (Dührkop et al., 2015, 2021; Djoumbou Feunang et al., 2016) were thereafter used to generate suggested structures and Classyfire hierarchical classes of organic compounds respectively (Djoumbou Feunang et al., 2016; Dührkop et al., 2021) for each feature separately. Sirius suggestions were manually evaluated by literature data to reach Metabolomic Standards Initiative (MSI) level 2 of identification.

### Metagenomic Analysis of the Fungal Communities

#### DNA Extraction

Eight accessions covering the chemical variability of the wider dataset, with 2 years × 4 replicates of each accession (*n* = 8 × 2 × 4) were selected for evaluation of their fungal endophytic communities. These accessions were selected to cover most of the range of the PCs of the chemical space of the samples.

Genomic DNA extracts were prepared with E.Z.N.A. Plant DNA DS Mini Kit (Omega Bio-Tek Inc., Norcross, GA, United States) and E.Z.N.A. Soil DNA Kit (Omega Bio-Tek Inc.) according to the manufacturer’s instructions, using ∼10 mg lyophilized plant material or ∼100 mg soil sample respectively. The DNA extraction protocol on empty microcentrifuge tubes and specimen jars was also carried out with both the plant and soil extraction kits for negative controls. An equimolar mixture of all horseradish DNA extract was also prepared as an average sample, similar to that used in QC of metabolomics, for the assessment of sequencing bias between identical samples. The DNA concentration of the extracts was quantified using a Nabi UV/Vis Nano Spectrophotometer (MicroDigital Co., Seongnam, South Korea).

#### Control of Universal Fungal Internal Transcribed Spacer Barcode Primers for Horseradish

Already available internal transcribed spacer (ITS) barcode primers (Toju et al., 2012) were shown to be suitable for testing fungal communities from different plant samples (Toju et al., 2019). To identify fungi from horseradish, we had to verify the extent to which the ITS region of the horseradish plant is amplified with these primers. As described in that publication, we amplified the entire ITS region of a horseradish clone using ITS1-F_KYO2 (Toju et al., 2012) and ITS4 (White et al., 1990), the ITS1 region is represented by ITS1-F_KYO2 and ITS2_KYO2 (Toju et al., 2012) primers, and the ITS2 region with ITS3_KYO2 (Toju et al., 2012) and ITS4 primers.

#### Primer Design and Evaluation of Performance

To have a specific primer pair that does not bind to the ITS2 region of the horseradish plant, but is specific to as many Ascomycota, Basidiomycota and “non-Dikarya” taxa as possible, we designed a new forward primer to complement the existing ITS4_KYO3 (5′–CTB TTV CCK CTT CAC TCG–3′) (Toju et al., 2012) reverse primer. An alignment of ITS sequences from previously described and studied fungal taxa from horseradish (e.g., *Fusarium*, *Macrophomina*, *Phoma*, etc.) (Szűcs et al., 2018; Plaszkó et al., 2020) and relevant plants (e.g., *Armoracia*, *Daucus*, etc.) was prepared with DECIPHER (Wright, 2020), and the consensus sequences were manually checked for prominent primer candidates. Primers with meaningful melting temperatures were considered for further *in silico* analyses. The forward primers designed by us and ITS4_KYO3 reverse primers were tested on horseradish clones and various fungal templates *in vitro* using the PCR protocol described by Toju et al. (2012), with 50°C annealing temperature. According to the analyses, the best performing candidate was ITS3_NOHR (5′–TTT CAA CAA CGG ATC TCT T–3′). The performance of the primer was also assessed with a more comprehensive *in silico* analysis using the UNITE 8.3 fungi ITS database (Abarenkov et al., 2021a), and a preliminary sequencing experiment, the results can be found in Supplementary Table 4 and section “Amplicon Sequencing.”

#### Library Preparation and Sequencing

For both the plant, soil and “QC” samples the ITS2 region was amplified, with primers ITS3_NOHR and ITS4_KYO3. The primer pairs contained the appropriate Illumina adapter sequences: 5′–TCG TCG GCA GCG TCA GAT GTG TAT AAG AGA CAG–3′ (forward), 5′–GTC TCG TGG GCT CGG AGA TGT GTA TAA GAG ACA G–3′ (reverse). Primer oligonucleotides were synthesized by Generi Biotech (Hradec Králové, Czechia). PCR reaction mixture for one sample contained the following: 5 μL genomic DNA (5 ng μL^–1^ concentration), 5-5 μL of each primer (1 μM), and 12.5 μL 2X KAPA HiFi HotStart ReadyMix (Roche, Basel, Switzerland). The DNA was amplified using a ProFlex PCR System (Thermo Fisher Scientific Inc.), and the following thermal program: denaturation at 95°C for 10 min, 35 amplification cycles (94°C for 20 s, 50°C for 30 sec, and 72°C for 20 s) and a final extension step at 72°C for 7 min. The second, indexing PCR reaction was prepared using Nextera XT Index Primer 1 (Illumina Inc., San Diego, CA, United States) and the following thermal program: denaturation at 95°C for 3 min, 8 amplification cycles (95°C for 30 s, 55°C for 30 s, and 72°C for 30 s) and finally 72°C for 5 min. PCR clean-up after both reactions were performed using AMPure XP beads (Beckman Coulter Inc., Brea, CA, United States). The DNA quantity of the libraries was assessed using a Qubit Fluorometer (Thermo Fisher Scientific Inc.), and quality analysis was carried out by BioAnalyzer DNA 1000 Chip (Agilent Technologies Inc., Santa Clara, CA, United States). An equimolar mixture of the libraries was sequenced with an Illumina MiSeq instrument using a MiSeq Reagent Kit v3 (600 cycles, 2 × 300 bp paired-end reads, Illumina) or a Miseq Reagent Nano v2 (150 cycles, 150 bp paired-end reads, Illumina) for the primer performance tests. Library preparation and sequencing were performed by Genomic Medicine and Bioinformatics Core Facility, University of Debrecen, Hungary. As most of the laboratory materials and surfaces may be contaminated with DNA and even the DNA purification kits have a unique microbiome or “kitome” (Hornung et al., 2019), we included 2-2 process controls using the plant DNA extraction kit and the soil DNA extraction kit.

#### Analysis of Metagenomic Data

Sequences were demultiplexed and FASTQ files were generated using the Illumina BaseSpace Sequence Hub service. Further sequence analyses were carried out using the DADA2 (Callahan et al., 2016) package in R (R Core Team, 2021). After optimization of the values, the first 19, 18 and the last 15, 60 nucleotides were trimmed from the forward and reverse reads (respectively) with the filterAndTrim function to remove primers and low-quality tails. Reads with more than 2 expected errors or containing ambiguous N nucleotides were removed during the filtering step (filterAndTrim parameters: maxEE = 2, maxN = 0, trimLeft = c(19, 18), trimRight = c(15, 60), truncQ = 2). After filtering, error models were created with randomized sampling (randomize = TRUE), then reads were dereplicated and DADA2 sample inference was performed using the default parameters. The filtered, denoised paired-end reads were merged if there was a minimum of 20 nucleotide overlap. The forward reads of the unmergeable pairs were also kept for downstream analysis, as some fungal genera have longer ITS2 sequences (Pauvert et al., 2019). As proposed by the authors of DADA2, we worked with ASVs rather than OTUs as (1) it is more beneficial if the merging of sequencing data of different sequencing runs is considered (Callahan et al., 2017) and (2) it is easier to distinguish unique contaminant sequences identified in the control samples. Discarding every single ASV detected in different negative controls would not be preferable, as it might result in the removal of ecologically valid sequences, making the biological interpretation harder (Nguyen et al., 2015). Therefore, only ASVs that were present in negative controls at a minimum of 0.5%, with at least 25 reads were discarded. Taxonomy assignment was carried out by the Naive Bayesian classifier (Wang et al., 2007) implemented in DADA2, using the UNITE 8.3 fungi ITS database (Abarenkov et al., 2021a). Taxonomy assignments were filtered by an 80% bootstrap confidence, and ASVs only assigned to the Kingdom level were discarded from further analysis. ASVs present in negative controls at 0.5% of all reads and >25 reads were considered artifacts and also excluded from further work. Taxonomic identifications in certain cases were validated by BLASTn searches in the RefSeq database using an E-value = 1E−70 threshold. Associated taxonomic levels for the RefSeq data were obtained from the Taxonomy Browser of the NCBI database.

#### Structural Diversity Measures of Metagenomic Data

Alpha and beta diversity of the samples was characterized and compared concerning the “type” set (Site1 horseradish, Site2 horseradish, Site1 carrot, and Site1 soil samples) and the “accession” set (horseradish samples from Site1). The taxonomic richness of the samples was estimated with the ACE index. The diversity of the samples was measured with Shannon, Dominance (1-Simpson) and Buzas and Gibson’s evenness indices. Kruskal–Wallis tests were used for the statistical comparisons of the taxonomic richness and diversity of the above-mentioned groups. Beta diversity was estimated with Bray–Curtis similarity, Whittaker diversity, and unweighted UniFrac distance. Principal Coordinates Analysis (PCoA) based on the similarities or distances was performed to visualize beta diversity. One-way analysis of similarity (ANOSIM) was performed to determine the differences within sets and between sets. In the cases of significantly dissimilar sets, SIMPER (Clarke, 1993) was used to determine the taxonomic units responsible for the dissimilarity. All diversity analyses were executed in Past v4.09 (Hammer et al., 2001) except the unweighted UniFrac analysis.

Since the occurrences of phylogenetically diverse species were expected in the samples, random sample sets of 200 ASVs were chosen to test the reliability of the tree building. A phylogenetic workflow consisting of MUSCLE (Edgar, 2004) for alignments, GBLOCKS (Castresana, 2000) for the curation of the alignments and PhyML (Guindon and Gascuel, 2003) for the phylogenies was constructed using the Phylogeny.fr platform (Dereeper et al., 2008) for preliminary tests. Substitution model selection was done by SMS (Lefort et al., 2017). According to Akaike Information Criterion (18516, 25156) the best model was GTR + G + I. Reliability of the created trees was estimated with aLRT (SH-like) (Anisimova and Gascuel, 2006). Unweighted UniFrac analysis was performed in R (R Core Team, 2021) with phyloseq (McMurdie and Holmes, 2013) using a GTR + G + I substitution model for the phylogenetic tree constructed with phangorn (Schliep, 2011) based on an alignment made with the algorithm provided by DECIPHER (Wright, 2020).

### Data-Mining for Correlations Between Metagenomic and Metabolomic Features

All additional calculations were implemented in R 4.1 (R Core Team, 2021). For downstream analysis, the ASVs with identical taxonomic assignments were pooled and the number of ASVs within each pool was not dealt with later on. Data were evaluated at four levels: abundances pooled at phylum, order and genus levels as well as diversity indices (previously calculated from raw non-pooled ASVs). When the identification was not available at the genus level, the deepest reliable identification level was used instead, resulting in mixed identification levels for each pooled ASV at order and genus examination levels. Correlation analysis of metagenomic and metabolomic features was only examined within the “accessions” dataset (horseradish accessions from Site 1, *n* = 8 × 2 × 4), while community differences among sample groups was examined using both the “accession” dataset and the “type” dataset (also containing Site 2 horseradish samples, carrot samples and soil samples). Site 2 horseradish samples were included to show that we are not doing any examination on a very distinct microbial community.

To be able to carry out the log-ratio transformation in order to remove the compositional nature of the obtained dataset (Gloor et al., 2017), inputting of zeroes was optimized and carried out. The percentage of zeroes was 71.7, 79.3, and 90.8% in the raw data at levels phylum, order, and genus, respectively. For comparison of sample types, and accessions, different inputting parameters were used, summarized in Supplementary Table 5. Optimal parameters were chosen to result in the highest retention of reads, and the highest retention of unique features, assuming the ratio of zeroes is smaller than 0.5. Thereafter, a Bayesian-multiplicative replacement of count zeroes was carried out with clr function of the zCompositions package (Palarea-Albaladejo and Martín-Fernández, 2015), followed by normalization by ilr according to Gloor et al. (2017) with the compositions package (van den Boogaart et al., 2022) in R.

Significant differences among sample types and samples of accessions were sought in a principal component (PC) regression approach. In brief, either the log-ratio transformed fungal abundance dataset or the autoscaled (van den Berg et al., 2006) chemical dataset was subjected to sparse principal component analysis (sPCA) of the mixOmics package (Rohart et al., 2017) in R. The maximum number of PCs was 6 and 12 for fungal and chemical data, respectively. Statistical tests were only conducted for PCs accounting for more than 2.5% SD of the total variance of the underlying dataset. The PC values were subjected to ANOVA models in R.

Spearman correlations were calculated in R using the cor.test function, between either sPCA scores or properly scaled data. The four approaches were as follows: “rawcordf,” correlation between fungal abundance and chemical feature abundance data; “p2cordf,” correlation between fungal sPCA scores and chemical sPCA scores; “p1cordf_f,” correlation between fungal sPCA scores and chemical feature abundance data; “p1cordf_c,” correlation between fungal abundance and chemical sPCA scores. All meaningful combinations were tested. Correlations were sought at all three levels for fungal data (phylum-aggregated, order-aggregated, and genus level). Diversity data were only subjected to the “rawcordf” approach. At *n* = 250, a confidence interval for all correlation values were also calculated. Raw correlations in the “rawcordf” approach were restricted to compounds with putative annotations or an RSD > 0.66.

### False Discovery Rate Adjustments

All *p*-values from any statistical tests were subjected together to a Benjamini–Hochberg procedure (*n* = 18004). All *p*-values presented in the paper are adjusted values.

## Results

### Glucosinolate Content of Horseradish Root Accessions

Major GSLs were sinigrin and gluconasturtiin, while glucobrassicin and glucoiberin were present in smaller amounts: Sinigrin and gluconasturtiin concentrations spanned the range 1.14 ± 0.66 to 3.43 ± 0.65%DW, and 0.36 ± 0.18 to 0.67 ± 0.31%DW, respectively. Glucoiberin and glucobrassicin were present at around 0.03 and 0.07%DW, respectively. The relative standard deviation within an accession was relatively high (Supplementary Table 6): 26.9 and 19.8% for sinigrin and gluconasturtiin respectively, while variability among accessions was relatively low compared to expectations. Data on the GSL amounts of the eight accessions (selected later for fungal community assessment) is available in Supplementary Table 6. For sinigrin, gluconasturtiin, glucobrassicin, and glucoiberin, the difference between the highest and lowest average values was 3.33, 1.99, 20.5, and 2.81-fold, respectively. The aliphatic sinigrin and glucoiberin were more influenced by accession (*p*_*unadj*_ = 0.0008 and 0.0049, respectively) than glucobrassicin and gluconasturtiin (*p*_*unadj*_ = 0.6510 and 0.0227, respectively). Overall, the year 2019 resulted in fewer aliphatic GSLs (sinigrin and glucoiberin) and the benzenic gluconasturtiin (*p*_*unadj*_ = 5.21E−7, 0.0019, and 0.0024, respectively), while the indolic glucobrassicin seemed to be unaffected by the year of harvest (*p*_*unadj*_ = 0.2465). Additional GSLs were assessed for their relative abundance among samples using untargeted metabolomics.

### Untargeted Metabolomics of Horseradish Roots

An automated peak detection by XCMS online resulted in 2,576 features (positive and negative ion mode combined). After removing isotopes and adducts, a set of 1,310 was subjected to assessment of reliability via calculating linearity and relative standard deviation using a serial dilution of QCs and reproducibility of in-sequence QCs respectively. At a maximal RSD threshold of 30% and minimal linearity of 0.8, 355 features could be kept for submission to further analysis. The complete list of features is available in Supplementary Table 7.

Of the above sample-derived features, compounds of a highly variable subset (*n* = 233) were subjected to MS/MS fragmentation and the putative structures suggested from the MS/MS spectra by Sirius were manually verified using literature data (MSI level 2 identification); structures that could not be verified are referred to by their putative compound class using the Classyfire hierarchy (Djoumbou Feunang et al., 2016) (MSI level 3 identification) throughout the paper or their m/z – retention time value pairs (no identification). The compounds with MSI level 2 identification are shown in Table 1. The list includes specialized metabolites like flavonoid glycosides (kaempferol aglycon) and other polyphenolic compounds such as a phenylpropanoid glycoside and a coumarin glycoside, as well as indole derivatives and primary metabolites like phospholipids, amino acid derivatives and peptides.

**TABLE 1:** Compounds identified at MSI level 2 from horseradish samples.

m/z	Pol.	Rt	Name	MS/MS fragments	References
166.0684	Positive	12.18	N,N-(Dimethyl)thiobenzamide	120.081, 149.042, 103.0545	Holmes and Benoit, 1971
247.1451	Positive	12.3	Indol-3-ylmethyl amino derivative	167.1067, 149.0962, 139.1119, 121.1016, 116.0709	Takasugi et al., 1988
251.0857	Positive	12.44	Indol-3-ylmethyl cysteine	205.0794, 187.0756, 162.0597, 130.0653	Takasugi et al., 1988
295.1299	Positive	11.82	γ-Glu-Phe	278.1016, 232.0966, 186.0914, 166.0863, 120.081	Lee et al., 2017
308.114	Positive	10.35	1-Hexosyl-indole-3-carboxaldehyde	146.06005	Takasugi et al., 1988
353.1066	Positive	13.49	Methoxy-coumarin hexoside	249.0757, 207.06532, 189.0545	Szűcs et al., 2019
357.1299	Positive	2.88	1-OH-indole-3-carboxylic acid Gly derivative	325.2126, 255.1705, 202.0487, 178.05, 160.0393, 145.0496, 134.0603, 127.0393, 109.0288	Takasugi et al., 1988
369.1195	Positive	12.81	5-O-Feruloylquinic acid	193.0862	Szűcs et al., 2019
423.1370	Positive	12.51	Cys-Cys-Pro-Thr	277.06765, 191.0672, 179.04861, 162.02196, 116.05315, 76.02218	Smith et al., 2005
454.2942	Positive	17.75	1-16:0-lysoPE	313.2736, 282.2781, 239.23677, 155.01043	Godzien et al., 2015
471.1042	Positive	11.02	3-Methylsulfinyl-propyl isothiocyanate glutathione conjugate	308.0918, 199.0714, 162.02189, 179.04857, 122.06358	Szűcs et al., 2019
480.31	Positive	18.06	1-18:0-lysoPE	339.28943, 308.29481, 265.2537, 155.01046	Godzien et al., 2015
518.3259	Positive	16.79	1-18:3-lysoPC	184.07345	Godzien et al., 2015
535.1106	Positive	13.49	Kaempferol derivative	282.705519	Szűcs et al., 2019
160.0394	Negative	11.19	Indole-3-carboxylic acid	132.0444	Takasugi et al., 1988
208.0612	Negative	10.38	Formyl tyrosine	191.0344, 164.0707, 146.0602	Blasiak and Clardy, 2010
247.0723	Negative	11.89	5-OH-indole-acetic acid hexoside	218.9611, 200.9504, 160.0394, 116.0492	Takasugi et al., 1988
388.0748	Negative	11.52	Pentyl GSL	274.9911, 259.0126, 195.0331, 146.0635	Mellon et al., 2002; Bell et al., 2015
447.0938	Negative	12.86	Kaempferol hexoside	285.041, 284.0331, 255.03, 227.0347, 151.0023	Szűcs et al., 2019
617.1502	Negative	12.98	Kaempferol dihexoside	493.1205, 285.041, 284.0332, 255.0302, 227.0341, 151.003	Szűcs et al., 2019
549.1256	Negative	13.13	Kaempferol dipentoside	285.04095, 284.03318, 255.02927, 178.99794	Szűcs et al., 2019

*Pol., polarity; Rt, retention time (min).*

Metabolomic Standards Initiative level 3 identification added features from several putative compound classes: 2 cyanogenic glycosides, a flavonoid glycoside, a glucosinolate, 3 lipids (or lipid-like structures), 9 amino acids derivatives and derivatives as well as 14 glycosides and 19 other apparently aromatic compounds (Supplementary Table 7).

As some accessions turned out to be chemically quite similar, we selected a subset that could serve as a chemically variable model set to search for chemical compound – fungal feature correlations. Based on PCA plots of the chemical data, and by manual evaluation of the covered range of abundance of putatively identified and high RSD compounds, eight accessions (with four samples from each year) were selected for further study.

A PC score value of the chemical space of these accessions was statistically significantly different among the selected accessions (*p* = 0.0332, Supplementary Table 8), telling us that the accessions show significant differences in chemistry. The changes in the metabolome are perhaps best shown by examination of the distribution of effect size values when comparing the 2 years or different accessions. By calculating the difference between the accessions containing the lowest and highest amount of a compound, and expressing this difference in the absolute value of standard deviations, effect size values were obtained for all chemical features. A median of 1.289 shows that a high amount of chemical variability of the accessions is present. On the other hand, by comparing the effect size of the year factor, it turned out that the median was as high as 0.4678, meaning a relatively high year-to-year variability of the chemistry of the roots. What is more, 59 of 359 chemical features had an absolute effect size above 1 for the year factor, likely due to differences in the weather of the years 2018 and 2019 (Supplementary Table 1). As we did not intend to assess chemical differences of various accessions, data were fed into further analysis models without grouping along accessions or years. As we will see later, this chemical variability enabled us to find direct positive and negative correlations between the abundance of chemical entities and endophytic fungi.

### Amplicon Sequencing

The use of the ITS2 subregion within the ITS region as a fungal barcode has its advantages (length variation could be lower, with more universal primer sites than the ITS1 subregion) and is accepted when using second-generation sequencing platforms (Ihrmark et al., 2012; Toju et al., 2012; Nilsson et al., 2019). Where living plant tissue is used as a source for fungal metabarcoding, it is appropriate to use at least one primer that amplifies the fungi over the plants (Toju et al., 2012). In an *in silico* preliminary pilot, previously designed universal primers were found to be ineffective in the horseradish-endophytic system due to amplification of host DNA (data not shown). Therefore, we designed a primer that does not amplify the ITS2 region of horseradish, but amplifies the ITS2 region of fungal community members using an existing reverse primer. A pilot sequencing experiment was conducted on Illumina which showed that >99 and >40% of the amplicons were assigned to the Viridiplantae ITS according to the UNITE 8.3 eukaryote database (Abarenkov et al., 2021b) in the case of ITS3_KYO2/ITS4_KYO3 and fITS7/ITS4_KYO3, respectively. With the proposed forward ITS3_NOHR/ITS4_KYO3 primer pair, the number of plant-associated ITS reads was less than 0.2%. Both the *in silico* and preliminary experimental sequencing results were convincing enough for the usage of this primer pair in later experiments.

The proposed forward primer showed affinity to 95.3% of Ascomycota and 85.8% of Basidiomycota unique ITS sequences in the UNITE 8.3 database (Abarenkov et al., 2021a), allowing one mismatch (which was most prevalent at bp 6 from the 5′ primer end). These results are comparable to the coverage achieved by ITS3_KYO2 and fITS7 (Ihrmark et al., 2012) in these phyla.

The filtering step discarding abundant ASVs also present in negative controls affected about 0.7% of unique ASVs, including human dermatophytes like *Malassezia*. The exclusion of ASVs with neither meaningful UNITE assignment nor NCBI RefSeq (fungal ITS) assignment had much more impact on the dataset as it resulted in discarding 78.5% of unique ASVs (and 26.35% of reads). By checking the 50 most abundant of these sequences in BLAST manually, all of these sequences turned out to belong to various non-ITS plant genes. The filtration steps resulted in 2,673 ASVs in total, and a median of 26,116 reads per sample.

The inclusion of the QC samples in the sequencing was quite valuable for the optimization of the filtering parameters, as all four aliquots of the QC samples have shown the same ASV composition with negligible deviation in abundances (data not shown). By this property it also seems to be sufficient to monitor sequencing errors, although this requires further research.

### Diversity Structures of the Metagenomes

Diversity of the samples was characterized with the consideration of the 2,673 ASVs that passed the main filters. Observed ASV richness (number of ASVs in each sample) among the horseradish samples of Site1, those of Site2 and carrots from Site1 had a 45.2, 45.4, and 39.5 mean values, with ranges 27–94, 24–109, and 29–48, respectively. In contrast, the soil samples from Site1 exhibited a 383.6 mean value (range 109–609). The estimated ACE (Supplementary Figure 1A) and Shannon (Supplementary Figure 1B) indices confirmed the observed tendencies. Investigation of the Dominance index (1-Simpson) indicated that some species were more abundant than others in the horseradish samples (Supplementary Figure 1C). Although the overall dominance was quite low in the samples, the carrot and soil samples exhibited a more even abundance compared to the horseradish samples (Supplementary Figure 1C). Independently from the estimated Dominance values, the Buzas and Gibson indexes indicated a low level of evenness within the samples, except the carrot samples (Supplementary Figure 1D). Thorough examination of the diversity indices indicated that the horseradish samples showed a substantial variety within their values (Supplementary Figures 1B–D).

To examine beta diversity among the type sets, Bray–Curtis similarity was used to assess differences in the ASV abundance, moreover Whittaker distances were calculated to estimate species turnover in the sample sets (Supplementary Figures 2A,B). Both beta diversity measures represented by PCoA showed that soil samples are extremely different from other sample types (Supplementary Figures 2A,B). Besides, the figures showed that the beta diversities exhibited higher values within the horseradish sets, than in other sample sets. The low R values in the ANOSIM tests (Supplementary Figures 2A,B) also supported this finding. Although the differences between type sets were greater than the differences within sets (because of the distance of the soil sample set), the horseradish sets had a wide variety for both beta diversity metrics.

For a more sophisticated approach, unweighted UniFrac analysis was performed. Since UniFrac analysis depends on a robust phylogenetic tree, several preliminary trees were built to test their reliability. As the curation of the multiple alignments showed that at least 100 well-aligned nucleotide sites could be used and the branch supports of the created trees (aLRT) exceeded an admissible level of 0.7 on average, the UniFrac analysis could be performed with confidence. PCoA plotting of the UniFrac distances showed a disposition similar to the previous results (Supplementary Figure 2C). ANOSIM tests of the UniFrac distances were also in agreement with the other ANOSIM test results, albeit with a higher variety within the soil samples (Supplementary Figure 2C).

Both the alpha and the beta diversity metrics suggested a substantial variety among and within the horseradish samples, which might be in context with the metabolome changes of the horseradish accessions.

### Fungal Community Differences Among Sample Types

**FIGURE 1:**
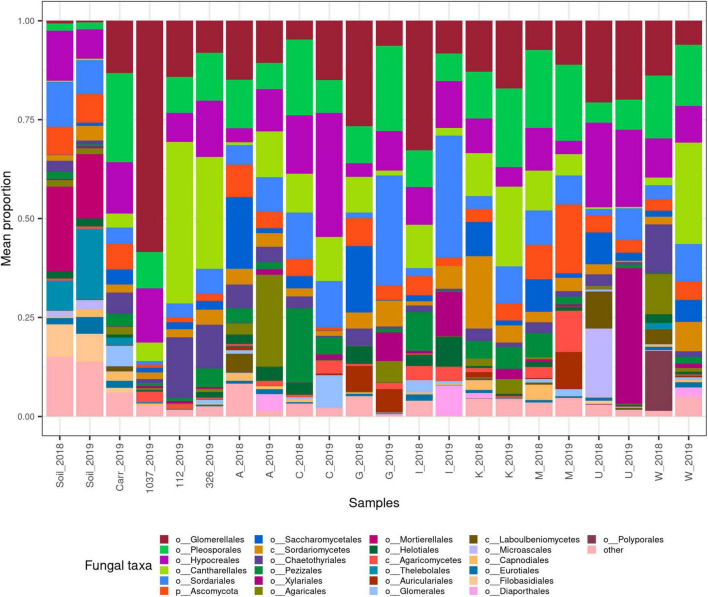
Bar plot showing relative mean proportion of fungal taxa in the endophytic fungal community in all sample groups (*n* = 4 per group). The top 25% of filtered, fungal reads are included and aggregated at order level (if not available, the lowest possible taxonomic level). Fungal taxa prefixes follow UNITE notation (“c__,” class; “o__,” order; “p__,” phylum). “Other” means a pool of ASVs from all other taxa. Sample codes: year_sample, where sample type is either “soil,” “carr” (soil or carrot both from site 1), 1,037 or 326 or 112 (site 2 horseradish samples from three soil types) or A-W (accession codes from site 1). Additional fungal composition data can be found on Supplementary Figures 3, 4 and Supplementary Tables 10–13.

The taxonomic assignments of the most abundant ASVs are summarized on Figure 1 and Supplementary Figures 3, 4. ANOVA of the fungal community among sample types has also shown significant differences at phylum, family and genus levels (*p* = 10^–18^, Supplementary Table 8), in concordance with PCoA results (Supplementary Figures 2A,B). In the case of Site 1 horseradish samples the most abundant ASVs belonged to Cantharellales, Glomerellales, Hypocreales, Pleosporales, Saccharomycetales and Sordariales, orders comprising numerous plant related genera (typical endophytes, epiphytes, pathogens, etc.), e.g., *Claviceps*, *Colletotrichum*, *Epichloë*, *Fusarium*, and *Rhizoctonia*. Almost the same composition was observed in the carrot and Site 2 horseradish samples. In addition, taxonomically less resolved mixtures of ASVs were from Ceratobasidiaceae, Nectriaceae, Pezizaceae, Sordariomycetes, and Ascomycota.

In contrast, the soil samples from Site 1 had a much different fungal community, mostly composed of Filobasidiales, Mortierellales, Hypocreales, Sordariales, Thelebolales, and Umbelopsidales orders. Typical abundant genera were *Mortierella, Pseudogymnoascus, Umbelopsis, Solicoccozyma, Metarhizium, Humicola, Penicillium*, *Trichoderma* and unidentified members of the Chaetomiaceae family and other Ascomycota – to mention the major ones only (Figure 1 and Supplementary Figures 3, 4). It is also important to note that soil communities in 2018 and 2019 showed only minor differences (Figure 1). This means that differences in the endophytic community of plant samples from different years are not the results of an altered fungal pool to recruit the fungi from.

Interestingly, in several cases, one or two taxa of endophytic fungi showed to be an extremely dominant feature (Supplementary Figure 4). What is more, this phenomenon was typical for horseradish samples of both sites, but not carrots from Site 1. This, though, might have been merely caused by differences in sampling depth (*n* = 64 + 12 vs. *n* = 4, respectively) but is apparent from differences in carrot and horseradish Dominance indices as well (Supplementary Figure 1C). Typical proportion of the most dominant strain to all reads was between 20 and 40% with a median of 33.57%, but in one sample, 95.9% was found (at genus level resolution) (Supplementary Figure 3). Soil samples showed considerably lower values, with a median of 21.61%.

As the soil–plant difference was much stronger compared to the variability among horseradish samples of Site 1, data from Site 1 horseradish samples was examined in further work separately. Several fungal groups showed a high variance among accessions, raising hopes to be able to build good correlation models later. Examples include taxa from Agaricomycetes (ranging 0.01–6.5%), Morosphaeriaceae and *Melanoleuca* (both ranging from n.d. to around 10%), *Monosporascus* (ranging from 0.04 to 17.1%) and *Setophoma* (ranging from <0.01 to 6.3%). As we will see later on, several of these differences correlated with chemical feature abundances. In some cases, considerable within-accession variability of the fungal endophytic community was detected at order level (Supplementary Figure 4). The average patterns were stable between the 2 years for accessions C, K, and M; accessions G and I showed very similar year-to-year changes at genus level, while a different year-to-year variability patterns can be observed for accessions A, U, and W (Supplementary Figure 3). A significant variance of taxa, later found to correlate with chemical features was also noted (including orders Xylariales, Sordariales, Pleosporales, Eurotiales, Capnoidales, and others).

### Chemical Feature – Fungal Abundance Correlations

The PCs of the family-level and genus-level aggregated fungal data set showed statistically significant Spearman correlation with various chemical features in 37 and 41 cases, respectively (*p* < 0.05, Supplementary Table 8). This high number encouraged us that instead of trying to interpret the loadings (the contributions to PCs) we should seek direct (Spearman) correlation between logratio-transformed fungal abundance data and chemical abundance data which is becoming a more widespread approach (Quinn and Erb, 2021). The advantage of this approach is that (1) it will not miss compounds that are not correlated with any other (and therefore would be present in an unexamined PC) and (2) interpretation is more straightforward. Disadvantages include the need to run a very high number of statistical tests, therefore corrections are needed to reduce the chance of false discoveries to an acceptable level (see section “False Discovery Rate Adjustments”). As the fungal dataset is not compositional [due to transformations (Gloor et al., 2017)], and the metabolome dataset is not compositional either (as data were expressed either as real concentrations or fold-change versus QCs for all features), no negative correlation artifacts are assumed (Morton et al., 2019), enabling usage of the approach. Though the family-level and the genus-level data clearly show some overlap if a family has a dominant genus, altogether 99 and 72 significant correlation phenomena could be found (*p* < 0.05, Supplementary Table 8, set = “rawcordf”) with this approach.

**FIGURE 2:**
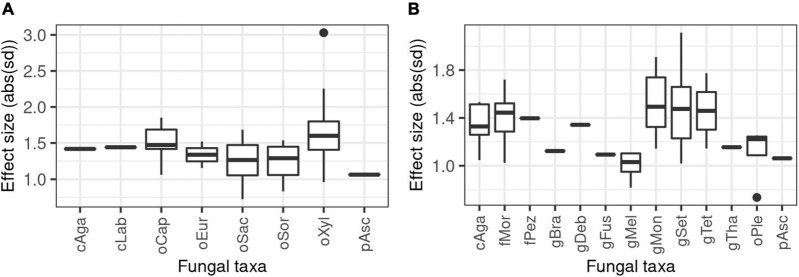
Effect size comparison of various fungal taxa, obtained from statistically significant correlations between logratio-transformed fungal abundance (proportion) data and core chemical feature abundance data. Phenomena were considered significant at *p* < 0.05 after Benjamini–Hochberg adjustment of *p*-values for all statistical tests. Effect size is defined as the difference of the fungal abundance data between the first and fourth quartile of the chemical data, expressed as absolute value of standard deviations. Subplots: **(A)** phenomena at order level resolution; **(B)** phenomena at genus level resolution. cAga, class Agaricomycetes; cLab, class Laboulbeniomycetes; fMor, family Morosphaeriaceae; fPez, family Pezizaceae; gBra, genus *Brachyphoris*; gDeb, genus *Debaryomyces*; gFus, genus *Fusarium*; gMel, genus *Melanoleuca*; gMon, genus *Monosporascus*; gSet, genus *Setophoma*; gTet, genus *Tetracladium*; gTha, genus *Thanatephorus*; oCap, order Capnodiales; oEur, order Eurotiales; oPle, order Pleosporales; oSac, order Saccharomycetales; oSor, order Sordariales; oXyl, order Xylariales; pAsc, phylum Ascomycota.

Order-level examination has revealed that the most impacted fungi include strains from the orders Xylariales, Capnodiales, Sordariales, and Saccharomycetales (median effect sizes for correlations being 1.60, 1.47, 1.29, and 1.26 standard deviations (*n* = 51, 15, 7, and 21) (Figure 2A). These taxonomic groups cover 6.14, 0.25, 8.34, and 4.45% of meaningful fungus-related reads in the whole accession dataset. It is important to note however that in some samples, as high as 73.4, 15.7, 74.2, and 38.4% of total reads were reached, respectively. A few additional taxa from other groups were found to be correlated with some chemical features, including taxa from Laboulbeniomycetes, Agaricomycetes, and Eurotiales (*n* = 1, 2, and 2, respectively). These all accounted for <1% of reads on average, however.

At the genus level, the abundance of *Monosporascus*, *Setophoma*, and *Tetracladium* as well as fungi from Morosphaeriaceae and Agaricomycetes were the most affected by changes in plant chemistry – median effect sizes for correlation phenomena being 1.49, 1.47, 1.45, 1.44, and 1.33 standard deviations (*n* = 10, 24, 2, 13, and 7), respectively (Figure 2B). These taxa accounted for 5.6, 1.3, 0.3, 1.5, and 0.8% of all reads, with their maximal proportion in the sample set being 73.4, 32.1, 28.6, 59.2, and 21.7%, respectively. Clearly, in several of the samples a single endophyte became dominant (Supplementary Figure 4). Additional fungal taxa also showed to be influenced, with less effect size (*Fusarium*, *Melanoleuca*, *Brachyphoris*, and *Thanatephorus* as well as fungi from Pezizaceae and Pleosporales), accounting for 4.0, 5.4, 0.4, 4.9, 2.4, and 3.2%. Overall, ASVs accounting for 35.23% of total reads were found to be significantly correlated with one or more chemical features in the accession dataset.

**FIGURE 3:**
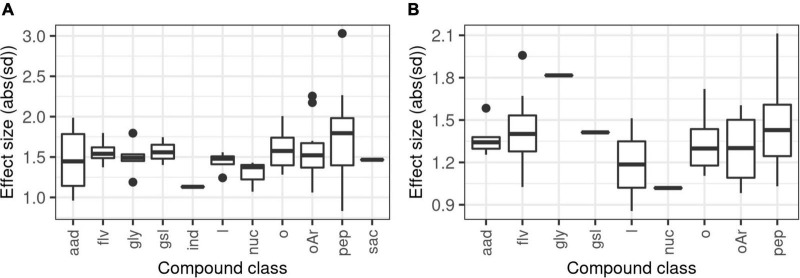
Effect size comparison of various metabolite classes, obtained from statistically significant correlations between logratio-transformed fungal abundance (proportion) data and core chemical feature abundance data. Phenomena were considered significant at *p* < 0.05 after Benjamini–Hochberg adjustment of *p*-values for all statistical tests. Effect size is defined as the difference of the fungal abundance data between the first and fourth quartile of the chemical data, expressed as absolute value of standard deviations. Subplots: **(A)** phenomena at order level resolution; **(B)** phenomena at genus level resolution. aad, amino acids and derivatives; flv, flavonoid glycosides; gly, glycosides; gsl, glucosinolates; ind, indole derivatives; l, lipids and lipid-like compounds; nuc, nucleotide derivatives; o, other compounds (unknown or non-annotated); oAr, other aromatic/polyphenolic compounds; pep, peptides; sac, saccharides.

The list of influential compounds includes natural products of many biosynthetic classes. Surprisingly, major GSLs did not result in any raw correlation with any fungal group, despite being present at high concentrations in the plant. Though there can be overlaps, order and genus level data were examined separately. The comparison of the effect size values of significant correlations between fungal orders and chemical correlations shows the following (Figure 3A): there is no single most influential compound group, though there are minor differences. A similar observation is apparent from the genus-level boxplot of significant correlations (Figure 3B). At first glance, the prejudicative assumption that GSLs are key contributors to shaping the fungal community does not seem to be evidenced in the data: GSL–fungal abundance correlations do not seem to be stronger than those of flavonoid glycosides or other putative glycosides. In addition, the contributions of lipids and lipid-like molecules seem to be similarly important, not to mention that of the peptides. In the latter case, however, a very high relative standard deviation is also present. The strongest effects (absolute effect size >2) seem to be related to peptide-like molecules, belonging to gamma-glutamyl amino acid derivatives and peptides according to the Canopus algorithm. The next group of correlations (absolute effect size >1.5) contain both specialized and primary metabolite classes as well: many flavonoid glycosides, a putative GSL and other putative glycosides show strong correlations with an abundance of some fungal taxa. In addition, several peptides, phospholipids and N, N-(dimethyl)thiobenzamide are also present in this group. The strongest impacts were shown for Xylariales, Capnodiales, and Saccharomycetales at the order level, while at the genus level, *Setophoma*, *Monosporascus*, and *Tetracladium* are worth mentioning.

## Discussion

### Identified Compounds

In addition to GSLs, horseradish roots were shown to contain kaempferol glycosides and phospholipids as well (Herz et al., 2017), supporting our findings summarized in Table 1. Isomers of the putative GSL identified were described from horseradish as breakdown products as isothiocyanate breakdown products (Blažević et al., 2020), though pentyl GSL (or methylbutyl GSL) were not found in *A. rusticana* samples (Agneta et al., 2012). Both the suggestions from the Canopus algorithm and the presence of the GSL characteristic fragment m/z 259.0126 (Rochfort et al., 2008) convinced us that it is a GSL. Other compounds include a coumarin glycoside, not specifically described from *A. rusticana* roots before, yet, known to be an important constituent of the Brassicaceae family member exudates (Sarashgi et al., 2021), so its presence is not a surprise. Indole-3-carboxaldehyde and other Trp-derived downstream products are also frequently encountered in research on Brassicaceae plants and were detected from various species of this plant family (Bednarek et al., 2011). Altogether, the suggestions generated by CSI:FingerID and Canopus significantly shortened the time required for annotation, though not all of these suggestions were found to be totally accurate on a closer look. In addition to the 21 compounds in Table 1 and Supplementary Table 7 contains a list of 69 compounds that could be classified into a compound class (“canopus_class” in Supplementary Table 7).

### Variability of Glucosinolate Content and Chemical Composition

Glucosinolates are precursors of antifungal components (Plaszkó et al., 2021) and are of primary importance, due to their role in the prevention of plant pathogenesis (Agee et al., 2010; Frerigmann et al., 2016; Kuhn et al., 2017). A comparison study on six Italian horseradish accessions reported similar ratios of sinigrin, gluconasturtiin, glucobrassicin, and glucoiberin as found in our dataset (Agneta et al., 2014). Year-to-year variability was likely due to the major differences in rainfall during the vegetation period, as temperature and rainfall are important for the production of this crop (Nguyen et al., 2013). Of more importance, the 2- to 20-fold differences between the major GSL concentrations among accessions were deemed enough to test these concentration data in Spearman correlations after autoscaling.

### Fungal Community Composition and Diversity

At genus resolution, plant samples showed a high abundance of genera that have been already described as endophytes: *Exophiala* (Yu et al., 2021), *Monosporascus* (Guo and Zou, 2020), *Paraphoma* (Kang et al., 2021), *Plectospherella* (Feng et al., 2021; Wei et al., 2021), *Podospora* (Penner and Sapir, 2021). Some of these genera were even identified in Brassicaceae plant endospheres, including *Exophiala* (Maciá-Vicente et al., 2016), *Plectosphaerella* (Plaszkó et al., 2020; Ważny et al., 2021), *Setophoma* (Szűcs et al., 2018; Poveda et al., 2020), or as a common pathogen: *Thanatephorus* (teleomorph of *Rhizoctonia*) (Dean et al., 2012).

All the alpha and beta diversity metrics used in this study unequivocally revealed substantial differences among the different sample types. As was expected, the soil samples exhibited the highest richness and almost a twofold diversity compared to the other samples. This is unsurprising, as many soil inhabitant fungi are not able to successfully colonize plant tissues, thus the plant microbiome is usually less diverse (Sasse et al., 2018). The lack of ability of saprotrophs to survive *in planta* conditions is a very powerful filter which is likely behind the similarity of carrot and horseradish samples – this surprising similarity of various root endophyte communities in distinct plant families has been reported by Toju et al. (2019).

On the other hand, indices of dominance and evenness besides the beta diversity measures revealed that the horseradish samples (from both sites) showed higher variety within their values than the soil samples. This is similar to the findings of Seabloom et al. (2019), who concluded that fungal endophyte communities differ within a single site, but are not consistently affected by the nutrient supply of hosts (which was manipulated as a treatment). Despite this, we will show later that given sufficient numbers of replicates of a single species, it is possible to successfully build models that show correlations between plant chemistry on fungal colonization. The relatively high dominances of a few strains (Figure 1) in most horseradish samples were also shown for several plant families (Toju et al., 2019). However, the extent is still striking and suggests that pioneers or fast-growing opportunists might occupy a significant portion of the plant niche. This also raises interesting questions regarding the number of replicates that should be included in such a study.

### Correlations Between Fungal Abundance and Chemical Abundance Data

**FIGURE 4:**
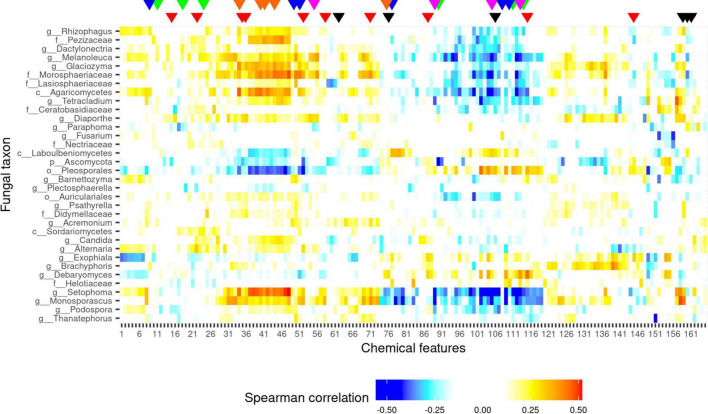
Heatmap of Spearman correlation values between logratio-transformed fungal abundance data (proportion) and core chemical feature abundance data. Both fungi and metabolic features are sorted by hierarchical clustering. Color is proportional to Spearman correlation values. Fungal taxa prefixes follow UNITE notation (“p__,” phylum; “o__,” order; “c__,” class; “f__,” family; and “g__,” genus). Chemical features marked with an arrow include: amino acid derivatives (red): 15, 22, 35, 36, 52, 58, 71, 87, 115, 145; indole derivatives (green): 11, 18, 24, 90, 111, 114; lipids (black): 62, 76, 106, 159-161; peptides (magenta): 105, 113, 89, 55; GSLs (blue): 9, 49, 51, 77, 108, 110; and flavonoid glycosides (orange): 34, 40, 41, 44, 75. For a complete list, see Supplementary Table 9. The Benjamini–Hochberg adjusted *p*-values of each correlation can be found on Supplementary Figure 5.

Figure 4 shows that there are several correlations between fungal and chemical features. What is more, several specialized and primary metabolite classes were shown to be involved in these interactions, including lipids, indolic compounds, glycosides, and peptides as well. Compounds showing high correlation with one or more fungal taxon are spread across various clusters (Figure 4), meaning that the results cannot be explained by multi-correlation of compounds only.

This shows the superiority of using a complex chemical evaluation strategy for plant-microbe interactions, such as untargeted metabolomics. Though this approach does not substitute knock-out mutant studies that can prove the contribution of a single compound in changing the plant microbiome (Voges et al., 2019), it shows that about a third of the plant endophytic fungal microbiome correlates with changes in the plant metabolome.

Our experimental design allowed searching for correlations, which should not be misinterpreted as causal relationships directly as there are several possible explanations behind them. Positive correlations between chemical features and fungal abundance can be the result of a recruitment – elicitation positive feedback loop. In these scenarios, the fungus attempts colonization by following chemical signals, produced by the plants (Sasse et al., 2018), followed by a plant response to limit fungal colonization. The biosynthesis of the compound stabilizes at a rate that the fungus can tolerate, but no additional plant reactions are triggered, resulting in a balanced antagonism (Schulz et al., 2015) between the fungus and the plant. If this rate of biosynthesis is higher than the basal rate, we possibly see a positive correlation between fungal abundance and the concentration of the metabolite. As we are seeing a snapshot of a dynamic, it is also possible though, that the concentration of a chemical feature increases due to increased penetration of the fungus, resulting in the release of for example amino acids or short peptides from proteins, or cell wall monomers. Note that during colonization of living tissue, special sets of cell wall degradation enzymes are used (Zuccaro et al., 2011). Anyway, due to various strategies, invasion by different fungal taxa results in a different mixture of plant biosynthetic compounds for defense (Narayani and Srivastava, 2017; You et al., 2021).

We will likely see negative correlations between chemical and fungal abundance features if the fungal entry attempt triggers the biosynthesis of a compound that successfully reduces fungal colonization: plants that are unable to biosynthesize such compounds will more likely end up being colonized by the strain of interest as shown for various GSL-derived products (Bednarek et al., 2009; Hiruma et al., 2010; Lipka et al., 2010; Sanchez-Vallet et al., 2010). As these networks are complex, this phenomenon is only assumed for compounds with known antifungal activity. Otherwise, seeing negative correlations between fungal colonization rate and compounds could also be explained by the depletion of monomers from plant tissues by the colonization of cells and the apoplastic space.

Flavonoid glycosides seem to be one of the most influential groups of compounds that correlate with fungal abundances (Figure 4). Lots of flavonoids including the detected kaempferol derivatives were shown to be antifungal agents *in vitro* (Jin, 2019; Al Aboody and Mickymaray, 2020) and there is also *in vivo* evidence of playing a role in fungal defense. Beyond studies that show an increased biosynthesis of flavonoids or downstream products after fungal challenge (Förster et al., 2022), increased accumulation at the fungal penetration site was also described in *Cucumis sativus* (McNally et al., 2003). As all correlations (with *Setophoma*, and members of Xylariales, Agaricomycetes, and Morosphaeriaceae) are positive, flavonoids likely play a role as agents in the establishment of beneficial interactions via the above-hypothesized colonization – elicitation feedback loop.

Fungal inhibition by GSL decomposition products has a large literature, but as they do not have antifungal effect in their native form, decomposition products are thought to be the actual agents required for fungal arrest (Plaszkó et al., 2022). Effects of GSLs on fungi seem to be mixed in the current dataset (Supplementary Table 8): while the abundance of Saccharomycetales negatively correlates with the putative GSL (Table 1), members of Xylariales and Morosphaeriaceae positively do. The major GSLs do not seem to show any correlation, but this might merely be because of the extreme amount of GSLs in horseradish compared to other Brassicaceae vegetables (Gonda et al., 2016), meaning that only downstream product generation is a prerequisite of fungal arrest, and no increase in the GSL biosynthesis is required (or possible).

Typical phytoalexin-like compounds unsurprisingly show negative correlations with fungal abundance (Supplementary Table 8): various ASVs pooled into unidentified Ascomycota were negatively correlated with an indole-3-carboxylic acid derivative. An indol-3-ylmethyl amino derivative and indol-3-ylmethyl cysteine were also active. Close relatives of these compounds are typical antifungal agents frequently encountered in *Arabidopsis thaliana* fungal challenge models where they are thought to play significant roles in restricting fungal growth *in planta* (Sanchez-Vallet et al., 2010; Bednarek et al., 2011; Gamir et al., 2014; Fukunaga et al., 2017; Kuhn et al., 2017). Therefore, we believe higher levels contribute to the inhibition of fungal colonization.

Typical peptides do not have significant antifungal activities, though there are known special peptides with antifungal activities (Chu et al., 2005), these are much larger than the ones found with a few amino acids only. Interestingly, the correlation of peptides 423.1387 at 12.58 and 471.1058 at 11.06 was massively negative: higher concentration resulted in less abundance of members of Xylariales, Capnodiales, Saccharomycetales, Sordariales, and Agaricomycetes as well as fungi from the genera *Setophoma, Monosporascus*, and *Melanoleuca*. What is more, one of these peptides was successfully identified as a glutathione – isothiocyanate adduct (Table 1), a product of isothiocyanate detoxification generated during fungal biotransformation of GSLs (Szűcs et al., 2018). A targeted search for the data in mzMine revealed the presence of 407.1071 at 11.86 and 471.1388 at 13.35, which are likely glutathione adducts of the isothiocyanates of sinigrin and gluconasturtiin, respectively, as well as and traces of the glucobrassicin-derived derivative as well. Interestingly, while in case of glucoiberin, the adduct showed a 0.709 correlation with the amount of glucoiberin, this value was below 0.35 for both the sinigrin-, and gluconasturtiin derived putative isothiocyanate adducts. This on one hand is reassuring, as it suggests that we are not seeing any artifact – myrosinase reaction products that arose during imperfections of homogenization at cryogenic temperature. On the other hand, it raises questions to be answered in further studies.

As isothiocyanate derivatives or adducts are known to be involved in defense using GSL decomposition products (Hiruma et al., 2013; Piślewska-Bednarek et al., 2018), this suggests that GSL decomposition is taking place without apparent change in GSL amounts. As mentioned above, this might be the result of the very high amount of the GSL pool in horseradish (Gonda et al., 2016), compared to other Brassicaceae plants. This also suggests that examining GSL downstream products instead of the precursors themselves can shed light on phenomena otherwise remaining hidden, when studying plant – endophytic fungus interactions. A wide range of downstream products are examined in *A. thaliana* studies (Bednarek et al., 2011), that fit this approach. The importance of glutathione in the generation of downstream products from GSL-derived isothiocyanates (and perhaps the prevention of autotoxicity) (Hiruma et al., 2013; Piślewska-Bednarek et al., 2018; Hématy et al., 2020) is hence further supported by our findings. The ability to biosynthesize various GSL downstream products is known to be a prerequisite of fungal colonization arrest (Bednarek et al., 2009; Hiruma et al., 2010; Lipka et al., 2010; Sanchez-Vallet et al., 2010; Fuchs et al., 2016), but the list of actual downstream products is incomplete (Frerigmann et al., 2016; Kuhn et al., 2017). Our findings strongly suggest that glutathione conjugates of the GSL decomposition product isothiocyanates can be such compounds and are likely good candidates for further study in plant-microbe interactions.

On the other hand, correlations with amino acid derivatives (typically smaller than the previously mentioned peptides) showed rather positive correlations, supporting the fungal recruitment theory, which is more likely for primary metabolites – an example of unsurprising usage as nutrients. It is also possible that increased fungal diversity results in a higher abundance of proteolytic enzymes in the apoplast, resulting in higher amounts of amino acids and derivatives for fungal uptake. Other primary metabolites (nucleotide derivatives, phospholipids, and other lipid-like molecules showed mixed correlations with fungal groups, Supplementary Table 8). Though there are special antifungal phospholipids reported (Cho et al., 1999), direct antifungal effects cannot be typically shown. Therefore, the reduction of the amounts might be simply the consequence of the usage of these compounds by fungi as sources of carbon and phosphorus.

## Conclusion

Altogether, metabolome changes in horseradish roots show correlations with the relative abundance of various endophytic fungi. Taxa giving about a third of fungal abundance was significantly correlated with one or more metabolomic features, showing the power of the untargeted metabolomics approach, but on the other hand, suggests the importance of inclusion of proteomic data and information from other domains in future studies. The correlation between chemical features could not explain all the observed phenomena: an example of success for using linear correlation mining between metabolomic and metagenomic data after proper data preparation steps were taken.

The high intra-species variability of the root endophytic community was suitable to detect correlations with chemical features, but it also raises questions about the optimal sampling depth of such studies. Note that in a few instances, the dominant taxon accounted for as high as >75% of fungal reads in a sample.

The untargeted metabolomics approach has resulted in lots of readily interpretable fungal abundance – chemical feature correlations. Interestingly, several specialized metabolite classes with known antifungal activity seem to be rather involved in the recruitment of fungal endophytes: flavonoid glycosides for instance showed positive correlations with many of the taxa. This could hypothetically be explained by a recruitment – elicitation feedback loop that stabilizes plant biosynthesis at a higher level that is still well tolerated by a small subset of potential colonizers.

On the other hand, several compounds showed negative correlations with fungal abundance, these include indolic phytoalexin-like molecules, a putative glucosinolate but not major glucosinolates and a glucosinolate downstream product. Literature data suggest that the phenomenon behind negative correlations is that these compounds are important contributors to restricting fungal colonization. An interesting finding is the lack of correlations with major GSLs. Our data suggested that GSLs are indeed used in fungal restriction in the form of downstream products, but perhaps their level is so high that no significant change in the GSL pool can be observed. We suggest glutathione – isothiocyanate adducts for monitoring GSL – ITC conversion *in planta*, one of which was putatively identified and showed a relatively strong negative correlation with the abundance of many fungi. These compounds definitely warrant further study.

## Data Availability Statement

The datasets presented in this study can be found in online repositories. The names of the repository/repositories and accession number(s) can be found below: https://figshare.com/, https://doi.org/10.6084/m9.figshare.19596631.

## Author Contributions

TP: data collection (all aspects), data evaluation (metagenomics), and writing of the draft. ZS: data evaluation (identification of chemical features and metabolomics). ZC: data collection (LC-ESI-MS, MS/MS). LÁ-S: data evaluation (diversity and fungal taxonomy). HC: data collection (primer design and tests, and primer coverage), data evaluation (fungal taxonomy), and proofreading. LG: maintenance of the various accessions and providing horseradish roots for study. GV: critical revision. SG: conceptualization, data evaluation (primer coverage, metabolomics, chemical – fungal correlations), writing of draft, and funding. All authors contributed to the article and approved the submitted version.

## Conflict of Interest

The authors declare that the research was conducted in the absence of any commercial or financial relationships that could be construed as a potential conflict of interest.

## Publisher’s Note

All claims expressed in this article are solely those of the authors and do not necessarily represent those of their affiliated organizations, or those of the publisher, the editors and the reviewers. Any product that may be evaluated in this article, or claim that may be made by its manufacturer, is not guaranteed or endorsed by the publisher.

## References

[B1] Abarenkov K. Zirk A. Piirmann T. Pöhönen R. Ivanov F. Nilsson R. H. (2021a). *UNITE General FASTA Release for Fungi*. UNITE Community. 10.15156/BIO/1280049

[B2] Abarenkov K. Zirk A. Piirmann T. Pöhönen R. Ivanov F. Nilsson R. H. (2021b). *UNITE QIIME Release for Eukaryotes*. UNITE Community. 10.15156/BIO/1264819

[B3] Abdel-Farid I. B. Jahangir M. van den Hondel C. A. M. J. J. Kim H. K. Choi Y. H. Verpoorte R. (2009). Fungal infection-induced metabolites in *Brassica rapa*. *Plant Sci.* 176 608–615. 10.1016/j.plantsci.2009.01.017

[B4] Agee A. E. Surpin M. Sohn E. J. Girke T. Rosado A. Kram B. W. (2010). MODIFIED VACUOLE PHENOTYPE1 is an Arabidopsis myrosinase-associated protein involved in endomembrane protein trafficking. *Plant Physiol.* 152 120–132. 10.1104/pp.109.145078 19880612 PMC2799351

[B5] Agneta R. Möllers C. De Maria S. Rivelli A. R. (2014). Evaluation of root yield traits and glucosinolate concentration of different *Armoracia rusticana* accessions in Basilicata region (southern Italy). *Sci. Hortic.* 170 249–255. 10.1016/j.scienta.2014.03.025

[B6] Agneta R. Rivelli A. R. Ventrella E. Lelario F. Sarli G. Bufo S. A. (2012). Investigation of glucosinolate profile and qualitative aspects in sprouts and roots of horseradish (*Armoracia rusticana*) using LC-ESI hybrid linear ion trap with fourier transform ion cyclotron resonance mass spectrometry and infrared multiphoton dissociation. *J. Agric. Food Chem.* 60 7474–7482. 10.1021/jf301294h 22779710

[B7] Al Aboody M. S. Mickymaray S. (2020). Anti-fungal efficacy and mechanisms of flavonoids. *Antibiotics* 9:45. 10.3390/antibiotics9020045 31991883 PMC7168129

[B8] Anisimova M. Gascuel O. (2006). Approximate likelihood-ratio test for branches: a fast, accurate, and powerful alternative. *Syst. Biol.* 55 539–552. 10.1080/10635150600755453 16785212

[B9] Bednarek P. Piślewska-Bednarek M. Svatoš A. Schneider B. Doubský J. Mansurova M. (2009). A glucosinolate metabolism pathway in living plant cells mediates broad-spectrum antifungal defense. *Science* 323 101–106. 10.1126/science.1163732 19095900

[B10] Bednarek P. Piślewska-Bednarek M. van Themaat E. V. L. Maddula R. K. Svatoš A. Schulze-Lefert P. (2011). Conservation and clade-specific diversification of pathogen-inducible tryptoph.an and indole glucosinolate metabolism in *Arabidopsis thaliana* relatives. *New Phytol.* 192 713–726. 10.1111/j.1469-8137.2011.03824.x 21793828

[B11] Bell L. Oruna-Concha M. J. Wagstaff C. (2015). Identification and quantification of glucosinolate and flavonol compounds in rocket salad (*Eruca sativa*, *Eruca vesicaria* and *Diplotaxis tenuifolia*) by LC–MS: highlighting the potential for improving nutritional value of rocket crops. *Food Chem.* 172 852–861. 10.1016/j.foodchem.2014.09.116 25442630 PMC4245720

[B12] Blasiak L. C. Clardy J. (2010). Discovery of 3-formyl-tyrosine metabolites from *Pseudoalteromonas tunicata* through heterologous expression. *J. Am. Chem. Soc.* 132 926–927. 10.1021/ja9097862 20041686 PMC2808729

[B13] Blažević I. Montaut S. Burèul F. Olsen C. E. Burow M. Rollin P. (2020). Glucosinolate structural diversity, identification, chemical synthesis and metabolism in plants. *Phytochemistry* 169:112100. 10.1016/j.phytochem.2019.112100 31771793

[B14] Broadhurst D. Goodacre R. Reinke S. N. Kuligowski J. Wilson I. D. Lewis M. R. (2018). Guidelines and considerations for the use of system suitability and quality control samples in mass spectrometry assays applied in untargeted clinical metabolomic studies. *Metabolomics* 14:72. 10.1007/s11306-018-1367-3 29805336 PMC5960010

[B15] Callahan B. J. McMurdie P. J. Holmes S. P. (2017). Exact sequence variants should replace operational taxonomic units in marker-gene data analysis. *ISME J.* 11 2639–2643. 10.1038/ismej.2017.119 28731476 PMC5702726

[B16] Callahan B. J. McMurdie P. J. Rosen M. J. Han A. W. Johnson A. J. A. Holmes S. P. (2016). DADA2: high-resolution sample inference from Illumina amplicon data. *Nat. Methods* 13, 581–583. 10.1038/nmeth.3869 27214047 PMC4927377

[B17] Castresana J. (2000). Selection of conserved blocks from multiple alignments for their use in phylogenetic analysis. *Mol. Biol. Evol.* 17 540–552. 10.1093/oxfordjournals.molbev.a026334 10742046

[B18] Cho K.-W. Seo Y.-W. Yoon T.-M. Shin J.-H. (1999). Purification and structure determination of antifungal phospholipids from a marine streptomyces. *J. Microbiol. Biotechnol.* 9 709–715.

[B19] Chu K. T. Xia L. Ng T. B. (2005). Pleurostrin, an antifungal peptide from the oyster mushroom. *Peptides* 26 2098–2103. 10.1016/j.peptides.2005.04.010 15941607

[B20] Clarke K. R. (1993). Non-parametric multivariate analyses of changes in community structure. *Aust. J. Ecol.* 18 117–143. 10.1111/j.1442-9993.1993.tb00438.x

[B21] Dean R. Van Kan J. A. L. Pretorius Z. A. Hammond-Kosack K. E. Di Pietro A. Spanu P. D. (2012). The top 10 fungal pathogens in molecular plant pathology. *Mol. Plant Pathol.* 13 414–430. 10.1111/j.1364-3703.2011.00783.x 22471698 PMC6638784

[B22] DeAngelis K. M. Brodie E. L. DeSantis T. Z. Andersen G. L. Lindow S. E. Firestone M. K. (2009). Selective progressive response of soil microbial community to wild oat roots. *ISME J.* 3 168–178. 10.1038/ismej.2008.103 19005498

[B23] Depke T. Franke R. Brönstrup M. (2019). CluMSID: an R package for similarity-based clustering of tandem mass spectra to aid feature annotation in metabolomics. *Bioinformatics* 35 3196–3198. 10.1093/bioinformatics/btz005 30649189

[B24] Dereeper A. Guignon V. Blanc G. Audic S. Buffet S. Chevenet F. (2008). Phylogeny.fr: robust phylogenetic analysis for the non-specialist. *Nucleic Acids Res.* 36 W465–W469. 10.1093/nar/gkn180 18424797 PMC2447785

[B25] Djoumbou Feunang Y. Eisner R. Knox C. Chepelev L. Hastings J. Owen G. (2016). ClassyFire: automated chemical classification with a comprehensive, computable taxonomy. *J. Cheminform.* 8:61. 10.1186/s13321-016-0174-y 27867422 PMC5096306

[B26] Dudzik D. Barbas-Bernardos C. García A. Barbas C. (2018). Quality assurance procedures for mass spectrometry untargeted metabolomics. a review. *J. Pharm. Biomed. Anal.* 147 149–173. 10.1016/j.jpba.2017.07.044 28823764

[B27] Dührkop K. Fleischauer M. Ludwig M. Aksenov A. A. Melnik A. V. Meusel M. (2019). SIRIUS 4: a rapid tool for turning tandem mass spectra into metabolite structure information. *Nat. Methods* 16 299–302. 10.1038/s41592-019-0344-8 30886413

[B28] Dührkop K. Nothias L.-F. Fleischauer M. Reher R. Ludwig M. Hoffmann M. A. (2021). Systematic classification of unknown metabolites using high-resolution fragmentation mass spectra. *Nat. Biotechnol.* 39 462–471. 10.1038/s41587-020-0740-8 33230292

[B29] Dührkop K. Shen H. Meusel M. Rousu J. Böcker S. (2015). Searching molecular structure databases with tandem mass spectra using CSI:FingerID. *Proc. Natl. Acad. Sci. U.S.A.* 112 12580–12585. 10.1073/pnas.1509788112 26392543 PMC4611636

[B30] Dunn W. B. Broadhurst D. Begley P. Zelena E. Francis-McIntyre S. Anderson N. (2011). Procedures for large-scale metabolic profiling of serum and plasma using gas chromatography and liquid chromatography coupled to mass spectrometry. *Nat. Protoc.* 6 1060–1083. 10.1038/nprot.2011.335 21720319

[B31] Edgar R. C. (2004). MUSCLE: multiple sequence alignment with high accuracy and high throughput. *Nucleic Acids Res.* 32 1792–1797. 10.1093/nar/gkh340 15034147 PMC390337

[B32] Evans A. M. O’Donovan C. Playdon M. Beecher C. Beger R. D. Bowden J. A. (2020). Dissemination and analysis of the quality assurance (QA) and quality control (QC) practices of LC–MS based untargeted metabolomics practitioners. *Metabolomics* 16:113. 10.1007/s11306-020-01728-5 33044703 PMC7641040

[B33] Feng L. Zhang A. X. Li L. Zhang X. J. Wang Z. Tan N. H. (2021). Diversity of cultivable endophytic fungi in two *Rubia* plants and their potential for production of anti-tumour Rubiaceae-type cyclopeptides. *Lett. Appl. Microbiol.* 73 759–769. 10.1111/lam.13571 34591984

[B34] Förster C. Handrick V. Ding Y. Nakamura Y. Paetz C. Schneider B. (2022). Biosynthesis and antifungal activity of fungus-induced O-methylated flavonoids in maize. *Plant Physiol.* 188 167–190. 10.1093/plphys/kiab496 34718797 PMC8774720

[B35] Frerigmann H. Piślewska-Bednarek M. Sánchez-Vallet A. Molina A. Glawischnig E. Gigolashvili T. (2016). Regulation of pathogen-triggered tryptophan metabolism in *Arabidopsis thaliana* by MYB transcription factors and indole glucosinolate conversion products. *Mol. Plant* 9 682–695. 10.1016/j.molp.2016.01.006 26802248

[B36] Fuchs R. Kopischke M. Klapprodt C. Hause G. Meyer A. J. Schwarzländer M. (2016). Immobilized subpopulations of leaf epidermal mitochondria mediate PENETRATION2-dependent pathogen entry control in Arabidopsis. *Plant Cell* 28 130–145. 10.1105/tpc.15.00887 26721862 PMC4746686

[B37] Fukunaga S. Sogame M. Hata M. Singkaravanit-Ogawa S. Piślewska-Bednarek M. Onozawa-Komori M. (2017). Dysfunction of Arabidopsis MACPF domain protein activates programmed cell death via tryptophan metabolism in MAMP-triggered immunity. *Plant J.* 89 381–393. 10.1111/tpj.13391 27711985

[B38] Gamir J. Pastor V. Kaever A. Cerezo M. Flors V. (2014). Targeting novel chemical and constitutive primed metabolites against *Plectosphaerella cucumerina*. *Plant J.* 78 227–240. 10.1111/tpj.12465 24506441

[B39] Gloor G. B. Macklaim J. M. Pawlowsky-Glahn V. Egozcue J. J. (2017). Microbiome datasets are compositional: and this is not optional. *Front. Microbiol.* 8:2224. 10.3389/fmicb.2017.02224 29187837 PMC5695134

[B40] Godzien J. Ciborowski M. Martínez-Alcázar M. P. Samczuk P. Kretowski A. Barbas C. (2015). Rapid and reliable identification of phospholipids for untargeted metabolomics with LC–ESI–QTOF–MS/MS. *J. Proteome Res.* 14 3204–3216. 10.1021/acs.jproteome.5b00169 26080858

[B41] Gonda S. Kiss-Szikszai A. Szűcs Z. Nguyen N. M. Vasas G. (2016). Myrosinase compatible simultaneous determination of glucosinolates and allyl isothiocyanate by capillary electrophoresis micellar electrokinetic chromatography (CE-MEKC). *Phytochem. Anal.* 27 191–198. 10.1002/pca.2615 27313156

[B42] Gowda H. Ivanisevic J. Johnson C. H. Kurczy M. E. Benton H. P. Rinehart D. (2014). Interactive XCMS online: simplifying advanced metabolomic data processing and subsequent statistical analyses. *Anal. Chem.* 86 6931–6939. 10.1021/ac500734c 24934772 PMC4215863

[B43] Guindon S. Gascuel O. (2003). A simple, fast, and accurate algorithm to estimate large phylogenies by maximum likelihood. *Syst. Biol.* 52 696–704. 10.1080/10635150390235520 14530136

[B44] Guo Z. Zou Z.-M. (2020). Discovery of new secondary metabolites by epigenetic regulation and NMR comparison from the plant endophytic fungus *Monosporascus eutypoides*. *Molecules* 25:4192. 10.3390/molecules25184192 32932749 PMC7570479

[B45] Hammer O. Harper D. Ryan P. (2001). PAST: paleontological statistics software package for education and data analysis. *Palaeontol. Electron.* 4 1–9.

[B46] Hématy K. Lim M. Cherk C. Piślewska-Bednarek M. Sanchez-Rodriguez C. Stein M. (2020). Moonlighting function of phytochelatin synthase1 in extracellular defense against fungal pathogens. *Plant Physiol.* 182 1920–1932. 10.1104/pp.19.01393 31992602 PMC7140922

[B47] Herz C. Tran H. T. T. Márton M.-R. Maul R. Baldermann S. Schreiner M. (2017). Evaluation of an aqueous extract from horseradish root (*Armoracia rusticana* radix) against lipopolysaccharide-induced cellular inflammation reaction. *Evid. Based Complement. Altern. Med.* 2017:1950692. 10.1155/2017/1950692 28182113 PMC5274677

[B48] Hiruma K. Fukunaga S. Bednarek P. Piślewska-Bednarek M. Watanabe S. Narusaka Y. (2013). Glutathione and tryptophan metabolism are required for *Arabidopsis* immunity during the hypersensitive response to hemibiotrophs. *Proc. Natl. Acad. Sci. U.S.A.* 110 9589–9594. 10.1073/pnas.1305745110 23696664 PMC3677473

[B49] Hiruma K. Kobae Y. Toju H. (2018). Beneficial associations between Brassicaceae plants and fungal endophytes under nutrient-limiting conditions: evolutionary origins and host–symbiont molecular mechanisms. *Curr. Opin. Plant Biol.* 44 145–154. 10.1016/j.pbi.2018.04.009 29738938

[B50] Hiruma K. Onozawa-Komori M. Takahashi F. Asakura M. Bednarek P. Okuno T. (2010). Entry mode-dependent function of an indole glucosinolate pathway in Arabidopsis for nonhost resistance against anthracnose pathogens. *Plant Cell* 22 2429–2443. 10.1105/tpc.110.074344 20605856 PMC2929114

[B51] Holmer R. Rutten L. Kohlen W. van Velzen R. Geurts R. (2017). “Chapter eight - commonalities in symbiotic plant-microbe signalling,” in *Advances in Botanical Research How Plants Communicate with their Biotic Environment*, ed. Becard G. (Cambridge, MA: Academic Press), 187–221. 10.1016/bs.abr.2016.11.003

[B52] Holmes J. L. Benoit F. (1971). The mass spectra of benzamide and thiobenzamide. *Org. Mass Spectrom.* 5 525–530. 10.1002/oms.1210050504

[B53] Hori Y. Fujita H. Hiruma K. Narisawa K. Toju H. (2021). Synergistic and offset effects of fungal species combinations on plant performance. *Front. Microbiol.* 12:713180. 10.3389/fmicb.2021.713180 34594312 PMC8478078

[B54] Hornung B. V. H. Zwittink R. D. Kuijper E. J. (2019). Issues and current standards of controls in microbiome research. *FEMS Microbiol. Ecol.* 95:fiz045. 10.1093/femsec/fiz045 30997495 PMC6469980

[B55] Hu L. Robert C. A. M. Cadot S. Zhang X. Ye M. Li B. (2018). Root exudate metabolites drive plant-soil feedbacks on growth and defense by shaping the rhizosphere microbiota. *Nat. Commun.* 9:2738. 10.1038/s41467-018-05122-7 30013066 PMC6048113

[B56] Huang A. C. Jiang T. Liu Y.-X. Bai Y.-C. Reed J. Qu B. (2019). A specialized metabolic network selectively modulates *Arabidopsis* root microbiota. *Science* 364:eaau6389. 10.1126/science.aau6389 31073042

[B57] Ihrmark K. Bödeker I. T. M. Cruz-Martinez K. Friberg H. Kubartova A. Schenck J. (2012). New primers to amplify the fungal ITS2 region – evaluation by 454-sequencing of artificial and natural communities. *FEMS Microbiol. Ecol.* 82 666–677. 10.1111/j.1574-6941.2012.01437.x 22738186

[B58] Ishimoto H. Fukushi Y. Tahara S. (2004). Non-pathogenic *Fusarium* strains protect the seedlings of *Lepidium sativum* from *Pythium ultimum*. *Soil Biol. Biochem.* 36 409–414. 10.1016/j.soilbio.2003.10.016

[B59] Jin Y.-S. (2019). Recent advances in natural antifungal flavonoids and their derivatives. *Bioorg. Med. Chem. Lett.* 29:126589. 10.1016/j.bmcl.2019.07.048 31427220

[B60] Kang L. He D. Wang H. Han G. Lv H. Xiao W. (2021). “Breeding on mountains” resulted in the reorganization of endophytic fungi in asexually propagated plants (*Ligusticum chuanxiong* Hort.). *Front. Plant Sci.* 12:740456. 10.3389/fpls.2021.740456 34858448 PMC8631752

[B61] Kuhn H. Lorek J. Kwaaitaal M. Consonni C. Becker K. Micali C. (2017). Key components of different plant defense pathways are dispensable for powdery mildew resistance of the Arabidopsis *mlo2 mlo6 mlo12* triple mutant. *Front. Plant Sci.* 8:1006. 10.3389/fpls.2017.01006 28674541 PMC5475338

[B62] Lacerda J. W. F. Siqueira K. A. Vasconcelos L. G. Bellete B. S. Dall’Oglio E. L. Sousa Junior P. T. (2021). Metabolomic analysis of *Combretum lanceolatum* plants interaction with *Diaporthe phaseolorum* and *Trichoderma spirale* endophytic fungi through 1H-NMR. *Chem. Biodivers.* 18:e2100350. 10.1002/cbdv.202100350 34399029

[B63] Lee Y. G. Cho J.-Y. Hwang E. J. Jeon T.-I. Moon J.-H. (2017). Glu–Phe from onion (*Allium cepa* L.) attenuates lipogenesis in hepatocytes. *Biosci. Biotechnol. Biochem.* 81 1409–1416. 10.1080/09168451.2017.1303358 28345482

[B64] Lefort V. Longueville J.-E. Gascuel O. (2017). SMS: smart model selection in PhyML. *Mol. Biol. Evol.* 34 2422–2424. 10.1093/molbev/msx149 28472384 PMC5850602

[B65] Lipka U. Fuchs R. Kuhns C. Petutschnig E. Lipka V. (2010). Live and let die - *Arabidopsis* nonhost resistance to powdery mildews. *Eur. J. Cell Biol.* 89 194–199. 10.1016/j.ejcb.2009.11.011 19963301

[B66] Maciá-Vicente J. G. Glynou K. Piepenbring M. (2016). A new species of Exophiala associated with roots. *Mycol. Prog.* 15:18. 10.1007/s11557-016-1161-4

[B67] Madloo P. Lema M. Francisco M. Soengas P. (2019). Role of major glucosinolates in the defense of kale against *Sclerotinia sclerotiorum* and *Xanthomonas campestris* pv. *campestris*. *Phytopathology* 109 1246–1256. 10.1094/PHYTO-09-18-0340-R 30920356

[B68] Mastan A. Bharadwaj R. Kushwaha R. K. Vivek Babu C. S. (2019). Functional fungal endophytes in *Coleus forskohlii* regulate labdane diterpene biosynthesis for elevated forskolin accumulation in roots. *Microb. Ecol.* 78 914–926. 10.1007/s00248-019-01376-w 31001657

[B69] McMurdie P. J. Holmes S. (2013). phyloseq: an R package for reproducible interactive analysis and graphics of microbiome census data. *PLoS One* 8:e61217. 10.1371/journal.pone.0061217 23630581 PMC3632530

[B70] McNally D. J. Wurms K. V. Labbé C. Bélanger R. R. (2003). Synthesis of C-glycosyl flavonoid phytoalexins as a site-specific response to fungal penetration in cucumber. *Physiol. Mol. Plant Pathol.* 63 293–303. 10.1016/j.pmpp.2004.03.005

[B71] Mellon F. A. Bennett R. N. Holst B. Williamson G. (2002). Intact glucosinolate analysis in plant extracts by programmed cone voltage electrospray LC/MS: performance and comparison with LC/MS/MS methods. *Anal. Biochem.* 306 83–91. 10.1006/abio.2002.5677 12069418

[B72] Morton J. T. Aksenov A. A. Nothias L. F. Foulds J. R. Quinn R. A. Badri M. H. (2019). Learning representations of microbe–metabolite interactions. *Nat. Methods* 16 1306–1314. 10.1038/s41592-019-0616-3 31686038 PMC6884698

[B73] Narayani M. Srivastava S. (2017). Elicitation: a stimulation of stress in in vitro plant cell/tissue cultures for enhancement of secondary metabolite production. *Phytochem. Rev.* 16 1227–1252. 10.1007/s11101-017-9534-0

[B74] Nguyen N. H. Smith D. Peay K. Kennedy P. (2015). Parsing ecological signal from noise in next generation amplicon sequencing. *New Phytol.* 205 1389–1393. 10.1111/nph.12923 24985885

[B75] Nguyen N. M. Gonda S. Vasas G. (2013). A review on the phytochemical composition and potential medicinal uses of horseradish (*Armoracia rusticana*) root. *Food Rev. Int.* 29 261–275. 10.1080/87559129.2013.790047

[B76] Nilsson R. H. Larsson K.-H. Taylor A. F. S. Bengtsson-Palme J. Jeppesen T. S. Schigel D. (2019). The UNITE database for molecular identification of fungi: handling dark taxa and parallel taxonomic classifications. *Nucleic Acids Res.* 47 D259–D264. 10.1093/nar/gky1022 30371820 PMC6324048

[B77] Palarea-Albaladejo J. Martín-Fernández J. A. (2015). zCompositions — R package for multivariate imputation of left-censored data under a compositional approach. *Chemom. Intell. Lab. Syst.* 143 85–96. 10.1016/j.chemolab.2015.02.019

[B78] Pang Z. Chen J. Wang T. Gao C. Li Z. Guo L. (2021). Linking plant secondary metabolites and plant microbiomes: a review. *Front. Plant Sci.* 12:621276. 10.3389/fpls.2021.621276 33737943 PMC7961088

[B79] Papp N. Gonda S. Kiss-Szikszai A. Plaszkó T. Lőrincz P. Vasas G. (2018). Ethnobotanical and ethnopharmacological data of *Armoracia rusticana* P. Gaertner, B. Meyer et Scherb. in Hungary and Romania: a case study. *Genet. Resour. Crop Evol.* 65 1893–1905. 10.1007/s10722-018-0663-0

[B80] Pauvert C. Buée M. Laval V. Edel-Hermann V. Fauchery L. Gautier A. (2019). Bioinformatics matters: the accuracy of plant and soil fungal community data is highly dependent on the metabarcoding pipeline. *Fungal Ecol.* 41 23–33. 10.1016/j.funeco.2019.03.005

[B81] Penner S. Sapir Y. (2021). Foliar endophytic fungi inhabiting an annual grass along an aridity gradient. *Curr. Microbiol.* 78 2080–2090. 10.1007/s00284-021-02437-5 33765191

[B82] Piślewska-Bednarek M. Nakano R. T. Hiruma K. Pastorczyk M. Sanchez-Vallet A. Singkaravanit-Ogawa S. (2018). Glutathione transferase U13 functions in pathogen-triggered glucosinolate metabolism. *Plant Physiol.* 176 538–551. 10.1104/pp.17.01455 29122987 PMC5761798

[B83] Plaszkó T. Szűcs Z. Kállai Z. Csoma H. Vasas G. Gonda S. (2020). Volatile organic compounds (VOCs) of endophytic fungi growing on extracts of the host, horseradish (*Armoracia rusticana*). *Metabolites* 10:451. 10.3390/metabo10110451 33171636 PMC7695154

[B84] Plaszkó T. Szűcs Z. Vasas G. Gonda S. (2021). Effects of glucosinolate-derived isothiocyanates on fungi: a comprehensive review on direct effects, mechanisms, structure-activity relationship data and possible agricultural applications. *J. Fungi* 7:539. 10.3390/jof7070539 34356918 PMC8305656

[B85] Plaszkó T. Szűcs Z. Vasas G. Gonda S. (2022). Interactions of fungi with non-isothiocyanate products of the plant glucosinolate pathway: a review on product formation, antifungal activity, mode of action and biotransformation. *Phytochemistry* 200:113245. 10.1016/j.phytochem.2022.113245 35623473

[B86] Pluskal T. Castillo S. Villar-Briones A. Oresic M. (2010). MZmine 2: modular framework for processing, visualizing, and analyzing mass spectrometry-based molecular profile data. *BMC Bioinformatics* 11:395. 10.1186/1471-2105-11-395 20650010 PMC2918584

[B87] Pongrac P. Vogel-Mikuš K. Poschenrieder C. Barceló J. Tolrà R. Regvar M. (2013). “*Arbuscular mycorrhiza* in glucosinolate-containing plants: the story of the metal hyperaccumulator *Noccaea* (*Thlaspi*) *praecox* (Brassicaceae),” in *Molecular Microbial Ecology of the Rhizosphere*, ed. de Bruijn F. J. (Hoboken, NJ: John Wiley & Sons, Ltd), 1023–1032. 10.1002/9781118297674.ch96

[B88] Poveda J. Zabalgogeazcoa I. Soengas P. Rodríguez V. M. Cartea M. E. Abilleira R. (2020). *Brassica oleracea* var. *acephala* (kale) improvement by biological activity of root endophytic fungi. *Sci. Rep.* 10:20224. 10.1038/s41598-020-77215-7 33214647 PMC7678862

[B89] Quinn T. P. Erb I. (2021). Examining microbe–metabolite correlations by linear methods. *Nat. Methods* 18 37–39. 10.1038/s41592-020-01006-1 33398187

[B90] R Core Team (2021). *R: A Language and Environment for Statistical Computing.* Vienna: R Foundation for Statistical Computing.

[B91] Robin A. H. K. Yi G.-E. Laila R. Hossain M. R. Park J.-I. Kim H. R. (2017). *Leptosphaeria maculans* alters glucosinolate profiles in blackleg disease–resistant and -susceptible cabbage lines. *Front. Plant Sci.* 8:1769. 10.3389/fpls.2017.01769 29075281 PMC5644266

[B92] Rochfort S. J. Trenerry V. C. Imsic M. Panozzo J. Jones R. (2008). Class targeted metabolomics: ESI ion trap screening methods for glucosinolates based on MSn fragmentation. *Phytochemistry* 69 1671–1679. 10.1016/j.phytochem.2008.02.010 18396302

[B93] Rodriguez R. J. White J. F. Jr. Arnold A. E. Redman R. S. (2009). Fungal endophytes: diversity and functional roles. *New Phytol.* 182 314–330. 10.1111/j.1469-8137.2009.02773.x 19236579

[B94] Rohart F. Gautier B. Singh A. Lê Cao K.-A. (2017). mixOmics: an R package for ‘omics feature selection and multiple data integration. *PLoS Comput. Biol.* 13:e1005752. 10.1371/journal.pcbi.1005752 29099853 PMC5687754

[B95] Sanchez-Vallet A. Ramos B. Bednarek P. López G. Piślewska-Bednarek M. Schulze-Lefert P. (2010). Tryptophan-derived secondary metabolites in *Arabidopsis thaliana* confer non-host resistance to necrotrophic *Plectosphaerella cucumerina* fungi. *Plant J.* 63 115–127. 10.1111/j.1365-313X.2010.04224.x 20408997

[B96] Sarashgi A. Puschenreiter M. Baune M. Paffrath V. Oburger E. Giehl R. F. H. (2021). Does the exudation of coumarins from Fe-deficient, soil-grown Brassicaceae species play a significant role in plant Fe nutrition? *Rhizosphere* 19:100410. 10.1016/j.rhisph.2021.100410

[B97] Sasse J. Martinoia E. Northen T. (2018). Feed your friends: do plant exudates shape the root microbiome? *Trends Plant Sci.* 23 25–41. 10.1016/j.tplants.2017.09.003 29050989

[B98] Schliep K. P. (2011). phangorn: phylogenetic analysis in R. *Bioinformatics* 27 592–593. 10.1093/bioinformatics/btq706 21169378 PMC3035803

[B99] Schulz B. Haas S. Junker C. Andrée N. Schobert M. (2015). Fungal endophytes are involved in multiple balanced antagonisms. *Curr. Sci.* 109, 39–45.

[B100] Seabloom E. W. Condon B. Kinkel L. Komatsu K. J. Lumibao C. Y. May G. (2019). Effects of nutrient supply, herbivory, and host community on fungal endophyte diversity. *Ecology* 100:e02758. 10.1002/ecy.2758 31306499

[B101] Smith O. Maille G. Want E. J. Qin C. Trauger S. A. Brandon T. R. (2005). METLIN: a metabolite mass spectral database. *Ther. Drug Monit.* 27 747–751. 10.1097/01.ftd.0000179845.53213.39 16404815

[B102] Szoboszlay M. White-Monsant A. Moe L. A. (2016). The effect of root exudate 7,4’-dihydroxyflavone and naringenin on soil bacterial community structure. *PLoS One* 11:e0146555. 10.1371/journal.pone.0146555 26752410 PMC4709137

[B103] Szűcs Z. Cziáky Z. Kiss-Szikszai A. Sinka L. Vasas G. Gonda S. (2019). Comparative metabolomics of *Tilia platyphyllos* Scop. bracts during phenological development. *Phytochemistry* 167:112084. 10.1016/j.phytochem.2019.112084 31415913

[B104] Szűcs Z. Plaszkó T. Cziáky Z. Kiss-Szikszai A. Emri T. Bertóti R. (2018). Endophytic fungi from the roots of horseradish (*Armoracia rusticana*) and their interactions with the defensive metabolites of the glucosinolate - myrosinase - isothiocyanate system. *BMC Plant Biol.* 18:85. 10.1186/s12870-018-1295-4 29743024 PMC5944135

[B105] Takasugi M. Monde K. Katsui N. Shirata A. (1988). Novel sulfur-containing phytoalexins from the Chinese cabbage *Brassica campestris* L. ssp. *pekinensis* (Cruciferae). *Bull. Chem. Soc. Jpn.* 61 285–289. 10.1246/bcsj.61.285 27682988

[B106] Toju H. Kurokawa H. Kenta T. (2019). Factors influencing leaf- and root-associated communities of bacteria and fungi across 33 plant orders in a grassland. *Front. Microbiol.* 10:241. 10.3389/fmicb.2019.00241 30837969 PMC6390183

[B107] Toju H. Tanabe A. S. Yamamoto S. Sato H. (2012). High-coverage ITS primers for the DNA-based identification of ascomycetes and basidiomycetes in environmental samples. *PLoS One* 7:e40863. 10.1371/journal.pone.0040863 22808280 PMC3395698

[B108] van den Berg R. A. Hoefsloot H. C. Westerhuis J. A. Smilde A. K. van der Werf M. J. (2006). Centering, scaling, and transformations: improving the biological information content of metabolomics data. *BMC Genomics* 7:142. 10.1186/1471-2164-7-142 16762068 PMC1534033

[B109] van den Boogaart K. G. Tolosana-Delgado R. Bren M. (2022). *Compositions: Compositional Data Analysis.* Available online at: https://CRAN.R-project.org/package=compositions [accessed March 11, 2022].

[B110] Voges M. J. E. E. E. Bai Y. Schulze-Lefert P. Sattely E. S. (2019). Plant-derived coumarins shape the composition of an *Arabidopsis* synthetic root microbiome. *Proc. Natl. Acad. Sci. U.S.A.* 116 12558–12565. 10.1073/pnas.1820691116 31152139 PMC6589675

[B111] Wang Q. Garrity G. M. Tiedje J. M. Cole J. R. (2007). Naive Bayesian classifier for rapid assignment of rRNA sequences into the new bacterial taxonomy. *Appl. Environ. Microbiol.* 73 5261–5267. 10.1128/AEM.00062-07 17586664 PMC1950982

[B112] Ważny R. Rozpa̧dek P. Domka A. Jêdrzejczyk R. J. Nosek M. Hubalewska-Mazgaj M. (2021). The effect of endophytic fungi on growth and nickel accumulation in *Noccaea* hyperaccumulators. *Sci. Total Environ.* 768:144666. 10.1016/j.scitotenv.2020.144666 33736318

[B113] Wei G. Ning K. Zhang G. Yu H. Yang S. Dai F. (2021). Compartment niche shapes the assembly and network of *Cannabis sativa*-associated microbiome. *Front. Microbiol.* 12:714993. 10.3389/fmicb.2021.714993 34675893 PMC8524047

[B114] White T. J. Bruns T. Lee S. Taylor J. (1990). Amplification and direct sequencing of fungal ribosomal RNA genes for phylogenetics. *PCR Protoc.* 18 315–322.

[B115] Wright E. S. (2020). RNAconTest: comparing tools for noncoding RNA multiple sequence alignment based on structural consistency. *RNA* 26 531–540. 10.1261/rna.073015.119 32005745 PMC7161358

[B116] You C. Qin D. Wang Y. Lan W. Li Y. Yu B. (2021). Plant triterpenoids regulate endophyte community to promote medicinal plant *Schisandra sphenanthera* growth and metabolites accumulation. *J. Fungi* 7:788. 10.3390/jof7100788 34682210 PMC8539763

[B117] Yu Y. Teng Z. Mou Z. Lv Y. Li T. Chen S. (2021). Melatonin confers heavy metal-induced tolerance by alleviating oxidative stress and reducing the heavy metal accumulation in *Exophiala pisciphila*, a dark septate endophyte (DSE). *BMC Microbiol.* 21:40. 10.1186/s12866-021-02098-1 33546601 PMC7863494

[B118] Zuccaro A. Lahrmann U. Güldener U. Langen G. Pfiffi S. Biedenkopf D. (2011). Endophytic life strategies decoded by genome and transcriptome analyses of the mutualistic root symbiont *Piriformospora indica*. *PLoS Pathog.* 7:e1002290. 10.1371/journal.ppat.1002290 22022265 PMC3192844

