# Supplementary material for: Correlations Between the Metabolome and the Endophytic Fungal Metagenome Suggests Importance of Various Metabolite Classes in Community Assembly in Horseradish (Armoracia rusticana, Brassicaceae) Roots
Source: Front Plant Sci. 2022 Jun 17;13:921008. doi: 10.3389/fpls.2022.921008 (PMC9247618; doi:10.3389/fpls.2022.921008)
Supplement: Supplementary file 1 [file Image_1.PDF]

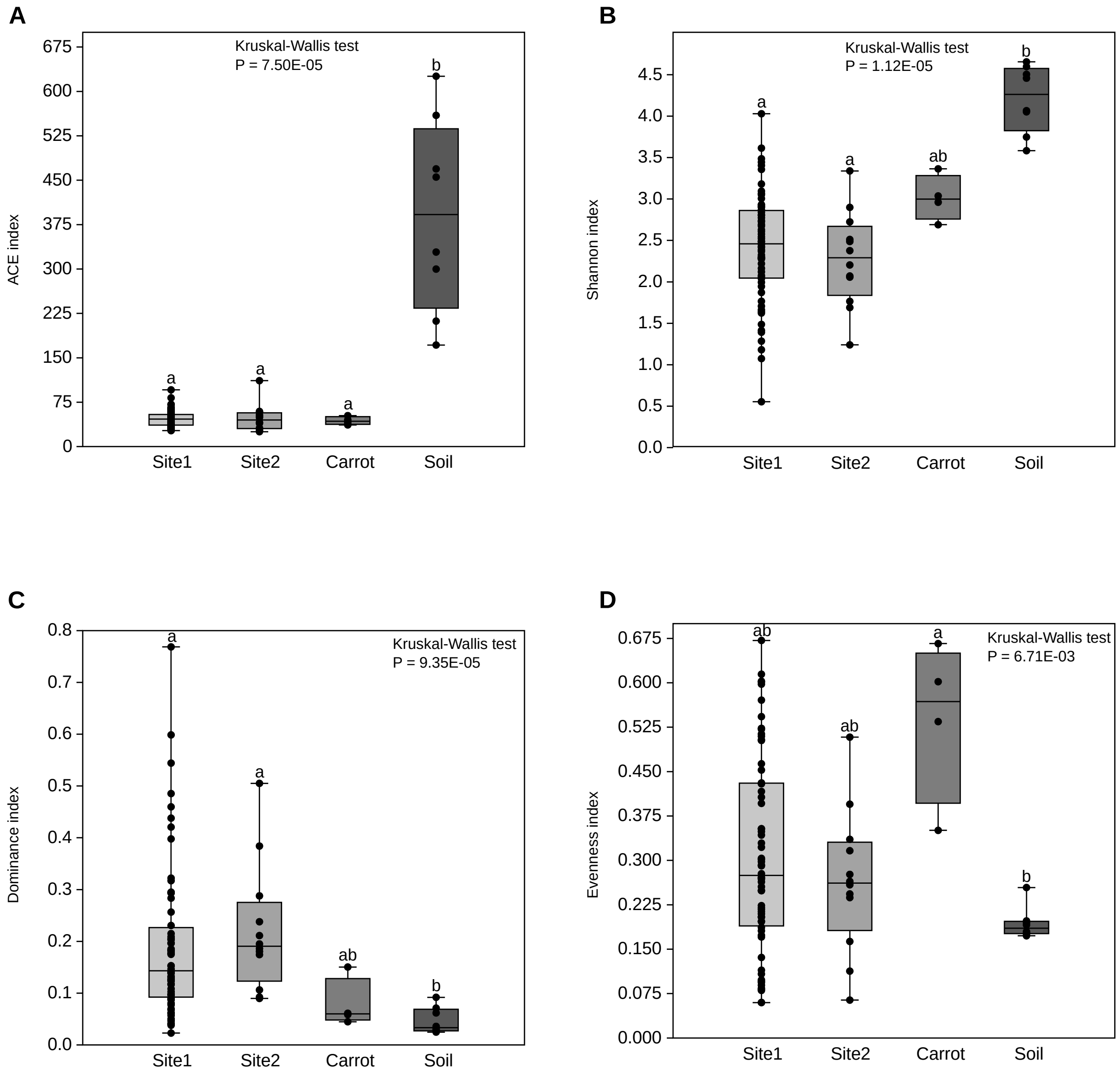

Figure S1. Key alpha diversity indices of fungal communities (total for soil samples and endophytic fungi for plant samples). (**A**) Species richness of the samples demonstrated with ACE index and (**B**) species diversity represented by Shannon index. The soil samples exhibited significantly higher richness and diversity than the other sample sets. (**C**) Dominance and (**D**) Buzas & Gibson evenness indices indicated a higher variety among the horseradish sample sets than within the other samples.

Box plots indicate the 25–75 percent quartiles. Horizontal lines within the boxes show the medians of the samples, minimal and maximal values are depicted by the whiskers. Black dots are individual values of the samples. On the X-axis Site1 and Site2 represent horseradish samples from different sites. Values of the different indices are depicted on the Y-axis. “a”, “b” and “ab” letters above the boxplots indicate similarity or significant discrepancy between the samples according to a Bonferroni corrected pairwise Dunn’s post-hoc test. In the case of the ACE index (**A**), the soil samples were significantly different from the others (P = 3.37E-05 (Site1); 0.0005558 (Site2); 0.01515 (Carrot)). In the Shannon (**B**) and Dominance (**C**) indices the Site1 and Site2 samples differed significantly from the soil sample (P = 2.82E-05; 9.67E-05 and 0.0006881; 0.0003656, respectively). As for the Buzas & Gibson (**D**) index, only the carrot and soil samples proved to be significantly different (P = 0.003761). All the other pairwise comparisons were not significantly different.
