# Supplementary material for: Correlations Between the Metabolome and the Endophytic Fungal Metagenome Suggests Importance of Various Metabolite Classes in Community Assembly in Horseradish (Armoracia rusticana, Brassicaceae) Roots
Source: Front Plant Sci. 2022 Jun 17;13:921008. doi: 10.3389/fpls.2022.921008 (PMC9247618; doi:10.3389/fpls.2022.921008)
Supplement: Supplementary file 2 [file Image_2.PDF]

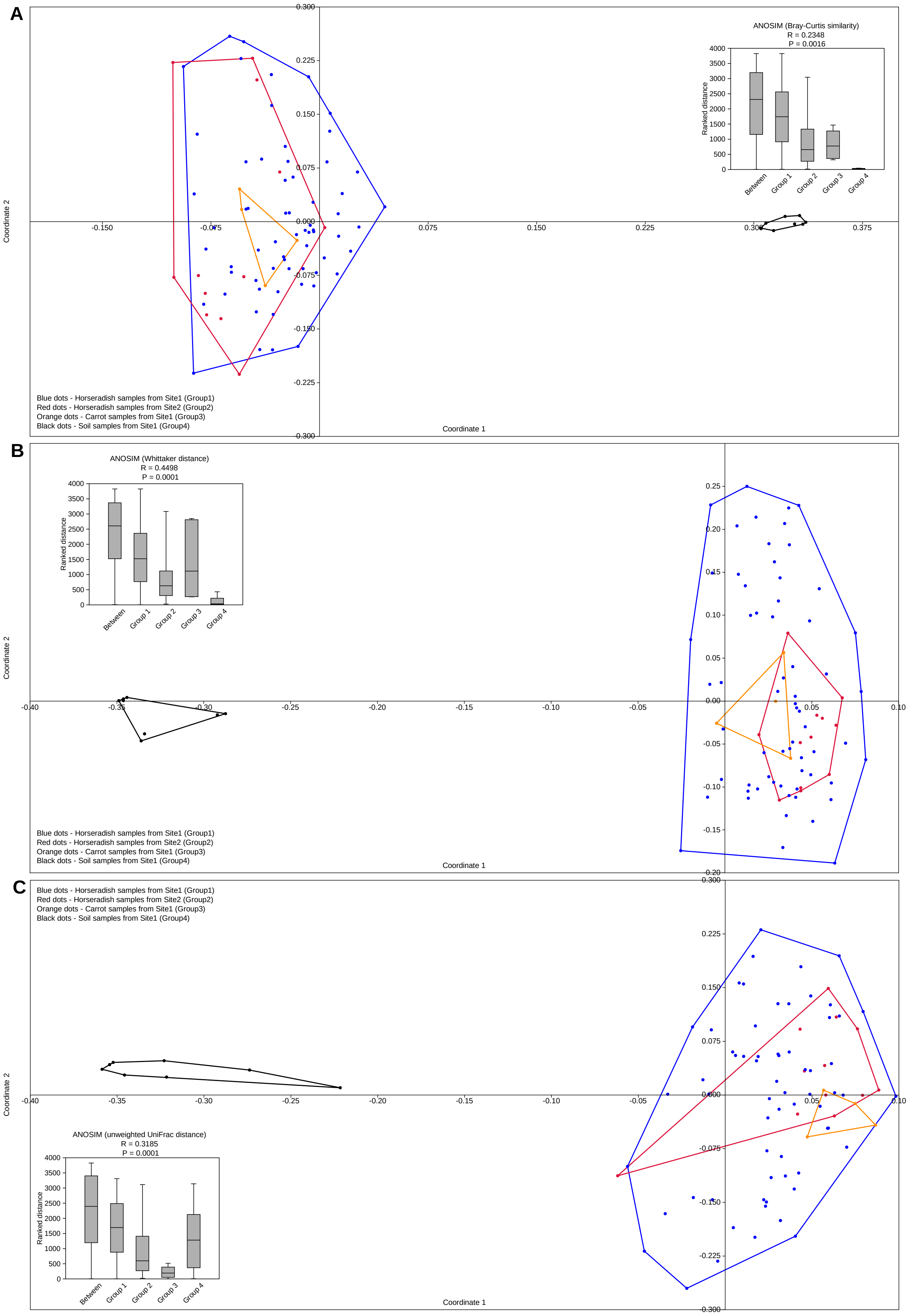

Figure S2. PCoA plots and ANOSIM results of beta diversity of the fungal communities (total for soil samples and endophytic fungi for plant samples). (**A**) PCoA plots created from abundance data using Bray-Curtis similarity index. (**B**) PCoA plots demonstrated species turnover calculated from Whittaker distances. (**C**) PCoA plots depicted phylogenetic distance measured with unweighted UniFrac analysis. Similarities between and within the sample sets examined with on-way ANOSIM tests. The low R values of the ANOSIM tests indicated that similarities between the sample sets do not differ strongly from the similarities within the sample sets.
