# Supplementary material for: Correlations Between the Metabolome and the Endophytic Fungal Metagenome Suggests Importance of Various Metabolite Classes in Community Assembly in Horseradish (Armoracia rusticana, Brassicaceae) Roots
Source: Front Plant Sci. 2022 Jun 17;13:921008. doi: 10.3389/fpls.2022.921008 (PMC9247618; doi:10.3389/fpls.2022.921008)
Supplement: Supplementary file 3 [file Image_3.PDF]

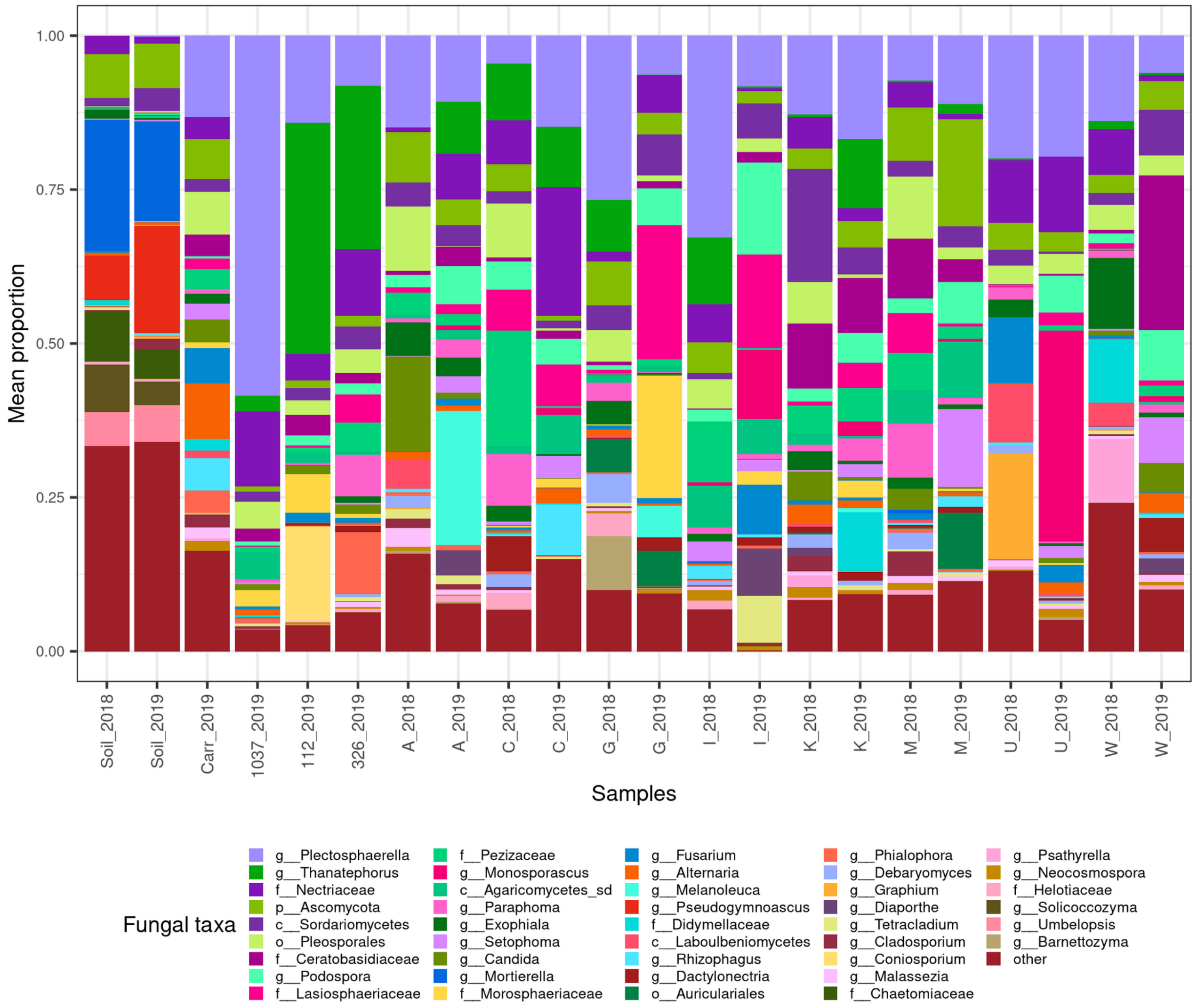

Figure S3. Bar plot showing relative mean proportion of fungal taxa in the endophytic fungal community in all sample groups (n = 4 per group). The top 10% of filtered, fungal reads are included and aggregated at genus level (if not available, the lowest possible taxonomic level). Fungal taxa prefixes follow UNITE notation (“c__”, class; “f__”, family; “g__”, genus; “o__”, order; “p__”, phylum). “Other” means a pool of ASVs from all other taxa. Sample codes: year_sample, where sample type is either “soil”, “carr” (soil or carrot both from site 1), 1037 or 326 or 112 (site 2 horseradish samples from three soil types) or A-W (accession codes from site 1).
