# Supplementary material for: Correlations Between the Metabolome and the Endophytic Fungal Metagenome Suggests Importance of Various Metabolite Classes in Community Assembly in Horseradish (Armoracia rusticana, Brassicaceae) Roots
Source: Front Plant Sci. 2022 Jun 17;13:921008. doi: 10.3389/fpls.2022.921008 (PMC9247618; doi:10.3389/fpls.2022.921008)
Supplement: Supplementary file 4 [file Image_4.PDF]

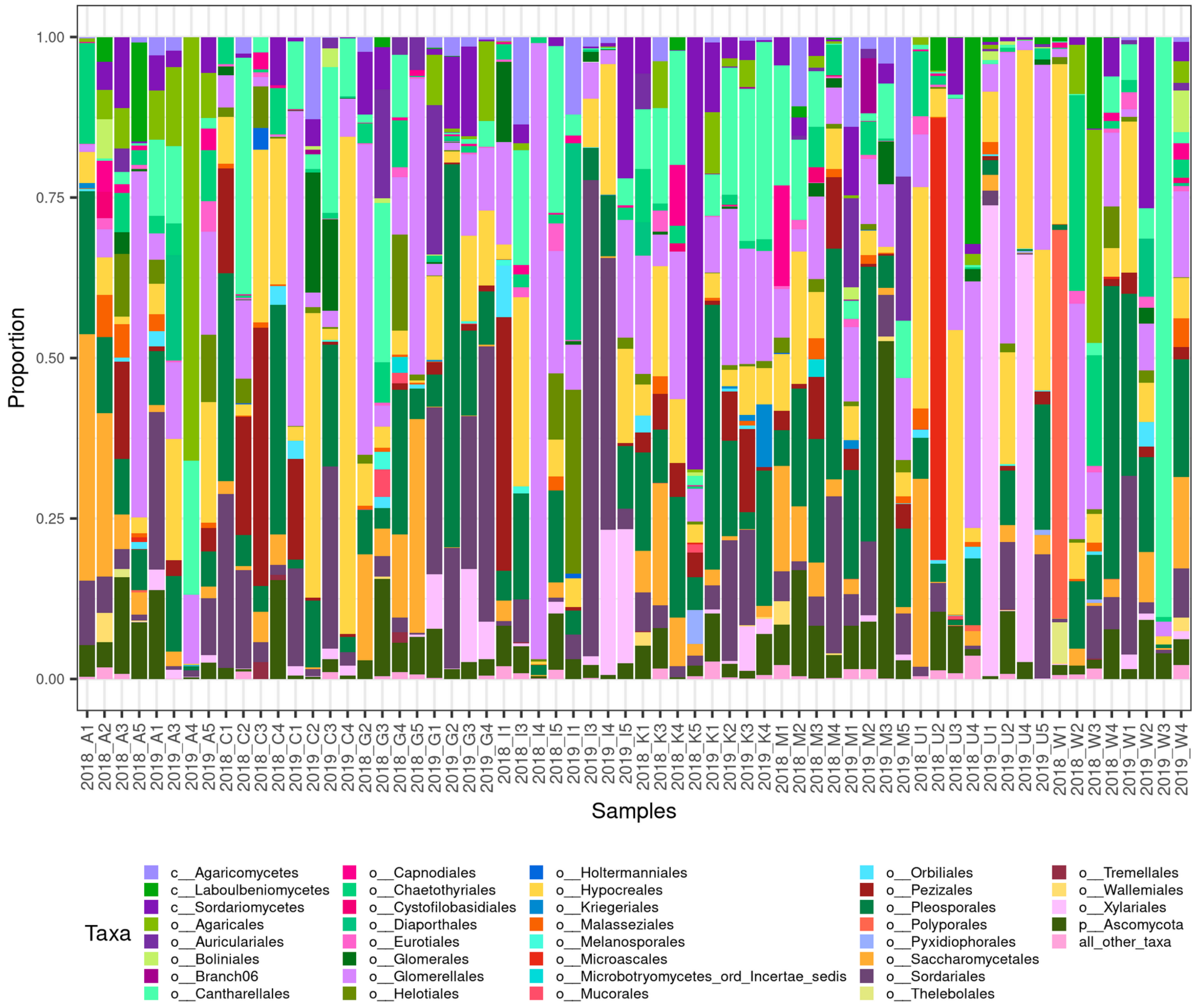

Figure S4. Bar plot showing relative mean proportion of fungal taxa in the endophytic fungal community in all samples. The top 10% of filtered, fungal reads are included and aggregated at genus level (if not available, the lowest possible taxonomic level). Fungal taxa prefixes follow UNITE notation ("c__", class; genus; "o__", order; "p__", phylum). "Other" means a pool of ASVs from all other taxa. Sample codes: year_accession_replicate, where "accession" can be A-W (accession codes from site 1).
