# Supplementary figures and images for: Correlations Between the Metabolome and the Endophytic Fungal Metagenome Suggests Importance of Various Metabolite Classes in Community Assembly in Horseradish (Armoracia rusticana, Brassicaceae) Roots
Source: Front Plant Sci. 2022 Jun 17;13:921008. doi: 10.3389/fpls.2022.921008 (PMC9247618; doi:10.3389/fpls.2022.921008)

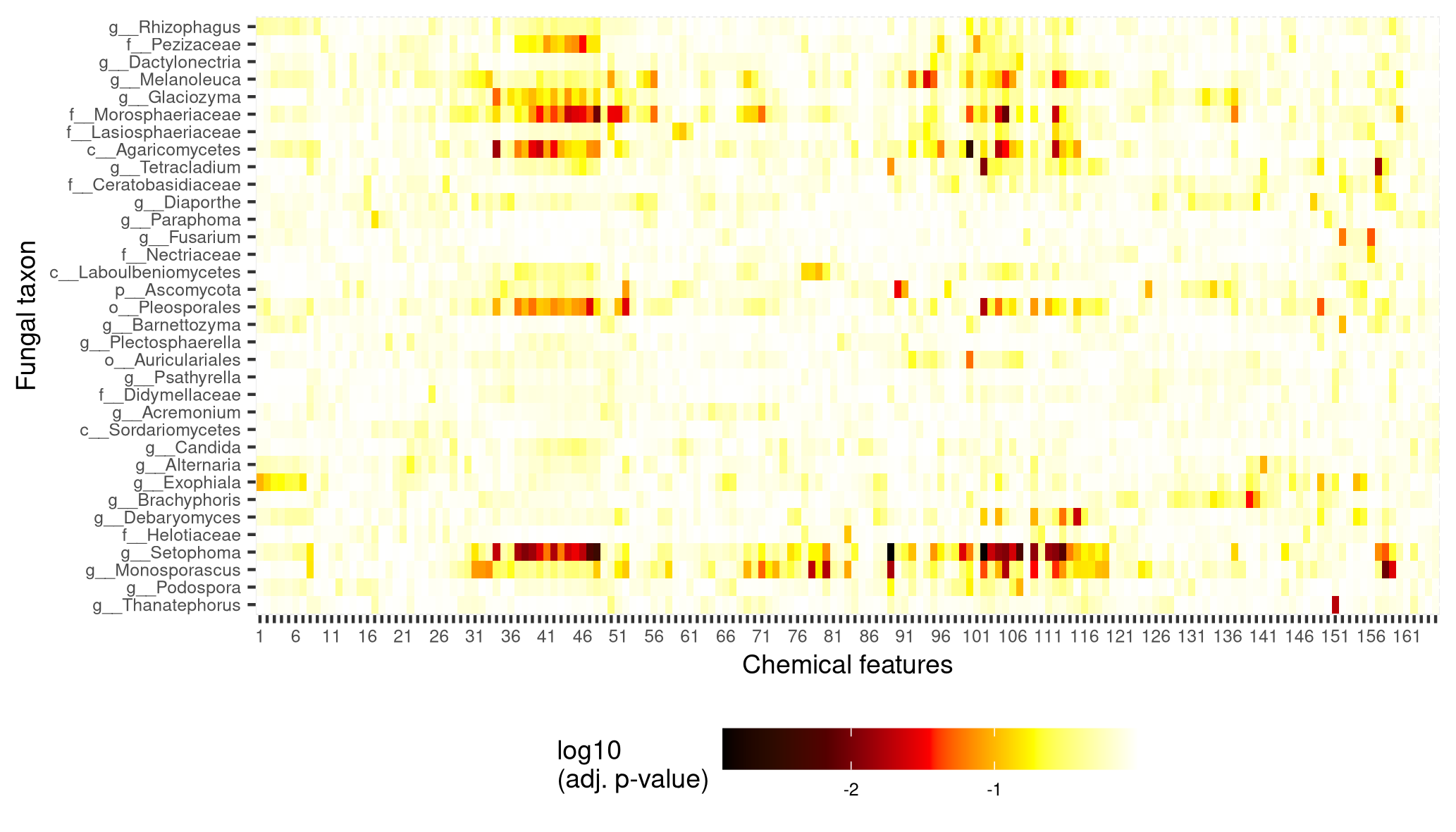

Supplement: Supplementary file 5 [file Image_5.TIFF]
