# Supplementary material for: Correlations Between the Metabolome and the Endophytic Fungal Metagenome Suggests Importance of Various Metabolite Classes in Community Assembly in Horseradish (Armoracia rusticana, Brassicaceae) Roots
Source: Front Plant Sci. 2022 Jun 17;13:921008. doi: 10.3389/fpls.2022.921008 (PMC9247618; doi:10.3389/fpls.2022.921008)
Supplement: Supplementary file 6 [file Table_1.PDF]

Table S1. Meteorological data of the 2018 and 2019 vegetation periods (2018.03.01. - 2018.11.08. and 2019.03.01 – 2019.11.06), obtained by interpolation of three official weather stations' data, nearest to Site 1.

**Parameter**	**Level**	**Value**	**Statistics**	**Unit**	**Interval**
Air temperature	NA	16.8	mean	°C	2018.03.01 – 2018.11.08.
Soil temperature	10 cm	19.2	mean	°C	2018.03.01 – 2018.11.08.
Soil temperature	20 cm	18.9	mean	°C	2018.03.01 – 2018.11.08.
Soil temperature	30 cm	18.4	mean	°C	2018.03.01 – 2018.11.08.
Soil temperature	45 cm	18.2	mean	°C	2018.03.01 – 2018.11.08.
Soil temperature	60 cm	17.8	mean	°C	2018.03.01 – 2018.11.08.
Soil temperature	75 cm	17.5	mean	°C	2018.03.01 – 2018.11.08.
Soil humidity	10 cm	12.9	mean	%	2018.03.01 – 2018.11.08.
Soil humidity	20 cm	14	mean	%	2018.03.01 – 2018.11.08.
Soil humidity	30 cm	13.9	mean	%	2018.03.01 – 2018.11.08.
Soil humidity	45 cm	14.3	mean	%	2018.03.01 – 2018.11.08.
Soil humidity	60 cm	20.1	mean	%	2018.03.01 – 2018.11.08.
Soil humidity	75 cm	21.3	mean	%	2018.03.01 – 2018.11.08.
Relative humidity	NA	71.8	mean	%	2018.03.01 – 2018.11.08.
Rainfall	NA	704.7	sum	mm	2018.03.01 – 2018.11.08.
Meteorological drought index	NA	1.68	mean	NA	2018.03.01 – 2018.11.08.
Water scarcity	35 cm	16.68	mean	mm	2018.03.01 – 2018.11.08.
Water scarcity	80 cm	6.09	mean	mm	2018.03.01 – 2018.11.08.
Air temperature	NA	15.8	mean	°C	2019.03.01. - 2019.11.06.
Soil temperature	10 cm	17.5	mean	°C	2019.03.01. - 2019.11.06.
Soil temperature	20 cm	17.3	mean	°C	2019.03.01. - 2019.11.06.
Soil temperature	30 cm	17	mean	°C	2019.03.01. - 2019.11.06.
Soil temperature	45 cm	16.8	mean	°C	2019.03.01. - 2019.11.06.
Soil temperature	60 cm	16.5	mean	°C	2019.03.01. - 2019.11.06.
Soil temperature	75 cm	16.4	mean	°C	2019.03.01. - 2019.11.06.
Soil humidity	10 cm	14.5	mean	%	2019.03.01. - 2019.11.06.
Soil humidity	20 cm	15.2	mean	%	2019.03.01. - 2019.11.06.
Soil humidity	30 cm	14.7	mean	%	2019.03.01. - 2019.11.06.
Soil humidity	45 cm	14.6	mean	%	2019.03.01. - 2019.11.06.
Soil humidity	60 cm	20.6	mean	%	2019.03.01. - 2019.11.06.
Soil humidity	75 cm	22.1	mean	%	2019.03.01. - 2019.11.06.
Relative humidity	NA	71.6	mean	%	2019.03.01. - 2019.11.06.
Rainfall	NA	1190.4	sum	mm	2019.03.01. - 2019.11.06.
Meteorological drought index	NA	1.18	mean	NA	2019.03.01. - 2019.11.06.
Water scarcity	35 cm	12.93	mean	mm	2019.03.01. - 2019.11.06.
Water scarcity	80 cm	3.45	mean	mm	2019.03.01. - 2019.11.06.
