# Supplementary material for: Correlations Between the Metabolome and the Endophytic Fungal Metagenome Suggests Importance of Various Metabolite Classes in Community Assembly in Horseradish (Armoracia rusticana, Brassicaceae) Roots
Source: Front Plant Sci. 2022 Jun 17;13:921008. doi: 10.3389/fpls.2022.921008 (PMC9247618; doi:10.3389/fpls.2022.921008)
Supplement: Supplementary file 7 [file Table_2.PDF]

Table S2. XCMS peak detection paramteres.

**Feature detection**	
ppm	2.5
minimum peak width	2.5
maximum peak width	25
mzdiff	0.01
Signal/Noise threshold	10
Integration method	1
prefilter peaks	3
prefilter intensity	5000
Noise Filter	1000
**Retention time correction**	
method	obiwarp
profStep	1
**Alignment**	
bw	5
minfrac	0.5
mzwid	0.02
minsamp	1
max	100
