# Supplementary material for: Correlations Between the Metabolome and the Endophytic Fungal Metagenome Suggests Importance of Various Metabolite Classes in Community Assembly in Horseradish (Armoracia rusticana, Brassicaceae) Roots
Source: Front Plant Sci. 2022 Jun 17;13:921008. doi: 10.3389/fpls.2022.921008 (PMC9247618; doi:10.3389/fpls.2022.921008)
Supplement: Supplementary file 8 [file Table_3.PDF]

Table S3. MzMine targeted peak detection parameters.

**Feature detection**	
intensity tolerance	50%
noise level	10
m/z tolerance (ppm)	5
m/z tolerance (Da)	0
retention time tolerance (min)	0.2
**Join Aligner**	
m/z tolerance (ppm)	5
m/z tolerance (Da)	0
retention time tolerance (min)	0.15
m/z weight	1
retention time weight	1
