# Supplementary material for: Correlations Between the Metabolome and the Endophytic Fungal Metagenome Suggests Importance of Various Metabolite Classes in Community Assembly in Horseradish (Armoracia rusticana, Brassicaceae) Roots
Source: Front Plant Sci. 2022 Jun 17;13:921008. doi: 10.3389/fpls.2022.921008 (PMC9247618; doi:10.3389/fpls.2022.921008)
Supplement: Supplementary file 9 [file Table_4.PDF]

Table S4. Coverage of the used forward primer ITS3_NOHR, compared to the widely used ITS3_KYO2 and ITSf7 primers in various fungal phyla according to in silico matching to UNITE 8.3, allowing a single mismatch. Typical mismatches were found in position 6 (not shown). Note coverage comparable to fITS7 and ITS3_KYO2 in major clades.

	Ratio			Unique sequences		
Phylum (UNITE)	ITS3_KYO2	ITS3_NOHR	fITS7	ITS3_KYO 2	ITS3_NO HR	fITS7
p__Ascomycota	0.985	0.953	0.943	14415	13943	13800
p__Basidiomycota	0.976	0.858	0.975	10152	8918	10141
p__Glomeromycota	0.987	0.911	0.763	300	277	232
p__Mucoromycota	0.978	0.479	0.768	261	128	205
p__Chytridiomycota	0.964	0.068	0.779	214	15	173
p__Mortierellomycota	0.955	0.883	0.964	106	98	107
p__unidentified	0.800	0.364	0.427	88	40	47
p__Rozellomycota	0.939	0.073	0.488	77	6	40
p__Neocallimastigomycota	0.816	0.000	0.474	31	0	18
p__Zoopagomycota	0.824	0.029	0.441	28	1	15
p__Kickxellomycota	1.000	0.038	0.577	26	1	15
p__Monoblepharomycota	1.000	0.000	0.579	19	0	11
p__Blastocladiomycota	0.750	0.000	0.125	12	0	2
p__Olpidiomycota	1.000	0.312	1.000	16	5	16
p__Entorrhizomycota	1.000	0.000	0.933	15	0	14
p__Basidiobolomycota	0.909	0.000	0.909	10	0	10
p__Entomophthoromycota	1.000	0.000	0.300	10	0	3
p__Aphelidiomycota	1.000	0.000	0.714	7	0	5
p__GS01	1.000	0.250	0.250	4	1	1
p__Calcarisporiellomycota	1.000	1.000	1.000	2	2	2
p__Fungi_phy_Incertae_sedis	1.000	1.000	1.000	1	1	1
