# Supplementary material for: Correlations Between the Metabolome and the Endophytic Fungal Metagenome Suggests Importance of Various Metabolite Classes in Community Assembly in Horseradish (Armoracia rusticana, Brassicaceae) Roots
Source: Front Plant Sci. 2022 Jun 17;13:921008. doi: 10.3389/fpls.2022.921008 (PMC9247618; doi:10.3389/fpls.2022.921008)
Supplement: Supplementary file 10 [file Table_5.PDF]

Table S5. Optimal feature discarding parameters and parameters of the resulting dataset used for subsequent imputting. Optimization was done by all combinations of min_prop values of 0.001, 0.003, 0.01, 0.03; min_occurrence values of 0.025, 0.05, 0.1, 0.2, 0.3, 0.4 for all levels. Abbreviations: kept_ratio, Proportion of kept unique features; kept_reads, Proportion of kept reads; min_prop, Minimal proportion in at least one sample; min_occurrence, Minimum occurrence in % of samples; zero_ratio, ratio of cells containing zero in the resulting dataset.

dataset	level	min_prop	min_occurrence	kept_ratio	zero_ratio	kept_reads
sample_type	Phylum	0.001	0.025	0.5333	0.4929	0.9999
sample_type	Family	0.03	0.025	0.3142	0.4876	0.9836
sample_type	Genus	0.001	0.200	0.0986	0.4926	0.7783
accessions	Phylum	0.001	0.025	0.4666	0.4642	1.0000
accessions	Family	0.01	0.025	0.3142	0.4900	0.9966
accessions	Genus	0.03	0.100	0.0802	0.4973	0.9383
