# Supplementary material for: Correlations Between the Metabolome and the Endophytic Fungal Metagenome Suggests Importance of Various Metabolite Classes in Community Assembly in Horseradish (Armoracia rusticana, Brassicaceae) Roots
Source: Front Plant Sci. 2022 Jun 17;13:921008. doi: 10.3389/fpls.2022.921008 (PMC9247618; doi:10.3389/fpls.2022.921008)
Supplement: Supplementary file 11 [file Table_6.PDF]

Table S6. Glucosinolate content expressed as %of dry weight (DW) in examined accessions (n = 4). Abbreviations: GBR, glucobrassicin; GIB, glucoiberin; GLN, gluconasturtiin; SIN, sinigrin. Accessions are shown with in-lab code.

Accession	Year	SIN mean	SIN SD	GLN mean	GLN SD	GIB mean	GIB SD	GBR mean	GBR SD
A	2018	2.769	0.686	0.603	0.112	0.017	0.012	0.072	0.074
C	2018	2.685	0.537	0.542	0.093	0.037	0.038	0.135	0.091
G	2018	3.435	0.646	0.492	0.053	0.073	0.064	0.083	0.064
I	2018	2.831	0.511	0.440	0.045	0.082	0.041	0.088	0.067
K	2018	2.608	0.196	0.498	0.031	0.046	0.047	0.077	0.062
M	2018	2.226	0.501	0.431	0.053	0.038	0.021	0.111	0.077
U	2018	2.679	0.746	0.337	0.052	0.032	0.020	0.071	0.049
W	2018	3.798	0.660	0.467	0.024	0.051	0.046	0.063	0.061
A	2019	2.567	0.527	0.629	0.126	0.004	0.003	0.074	0.010
C	2019	1.947	0.602	0.538	0.062	0.020	0.013	0.054	0.012
G	2019	1.837	0.820	0.670	0.307	0.011	0.010	0.057	0.022
I	2019	2.983	1.121	0.587	0.066	0.038	0.027	0.058	0.015
K	2019	1.915	0.254	0.480	0.073	0.032	0.023	0.057	0.030
M	2019	1.141	0.667	0.383	0.204	0.009	0.011	0.056	0.019
U	2019	2.112	0.264	0.625	0.077	0.010	0.007	0.057	0.013
W	2019	1.707	0.973	0.359	0.185	0.008	0.007	0.048	0.014
