# Supplementary material for: Correlations Between the Metabolome and the Endophytic Fungal Metagenome Suggests Importance of Various Metabolite Classes in Community Assembly in Horseradish (Armoracia rusticana, Brassicaceae) Roots
Source: Front Plant Sci. 2022 Jun 17;13:921008. doi: 10.3389/fpls.2022.921008 (PMC9247618; doi:10.3389/fpls.2022.921008)
Supplement: Supplementary file 12 [file Table_7.PDF]

Table S7. Chemical features found in horseradish roots using the quality controlled untargeted metabolomic approach and their putative identification (separate CSV file). Column legend (in order of appearance): mzmed, m/z of chemical feature; rtmed, retention time of chemical feature; polarity, 1 or -1 denotes positive or negative; putative_identification, MSI level 2 identification supported manual comparison with references, as in Table 1; manual_class, manually assigned compound class based on Table 1 putative identifications or canopus_class and CSI:FingerID suggestions; canopus_class, "most specific class" suggestion in the Classyfire hierarchy by the canopus algorithm.

mzmed	rtmed	polarity	putative_identification	manual_class	canopus_class
450.3797	20.18	1			
290.2121	17.48	1			
451.3823	20.28	1			
216.1602	13.99	1			
268.2277	17.81	1			
433.3536	20.3	1			
293.2115	17.59	1			
170.1177	20.02	1			
246.0612	3.11	1			
287.0559	12.86	1			
314.7173	12.69	1			
451.1106	3.11	1			
245.2265	18.39	1			
684.2572	3.04	1			
244.1547	12.9	1			
703.1954	3.06	1			
487.1668	3.05	1		Saccharides	Oligosaccharides
204.0158	11.29	1		other compounds	Organonitrogen compounds
212.1651	14.58	1			
202.1447	12.36	1			
376.2031	15.2	1			
224.1646	16.59	1			
219.0291	2.89	1			
216.1235	12.45	1			
295.2468	20.01	1			
266.2482	17.41	1			
489.1945	3.04	1			
204.1236	11.54	1			
220.1191	10.81	1			
170.1542	13.99	1			
434.3567	20.17	1			
452.3852	20.17	1			
775.1369	3.02	1			
438.1319	3.12	1			
212.1288	13.12	1			
793.2335	3.11	1		flavonoid glycosides	Flavonoid-3-O-glycosides
622.6707	3.1	1			
166.069	12.18	1	N,N-(Dimethyl)thiobenzamide	other compounds	Aralkylamines
451.6123	3.12	1			
351.2569	19.05	1			
162.0557	11.19	1	Indole 3 carboxylic acid	other aromatic compounds	Aryl-aldehydes
480.1706	12.3	1		other aromatic compounds	Anisoles
178.1269	10.14	1		other compounds	Sulfoxides
410.11	12.3	1			
349.062	3.1	1			
212.1649	15.03	1			
182.0834	6.85	1			
449.1098	12.86	1	Kaempferol-hexoside	flavonoid glycosides	
705.1867	3	1		Glycosides	O-glycosyl compounds
185.1369	13.43	1			
793.7291	3.09	1		Lipids and lipid-like molecules	Glycosyldiacylglycerols
203.1408	10.08	1			
518.3259	16.79	1	1-18:3-lysoPC	Lipids and lipid-like molecules	Lysophosphatidylcholines
287.056	13.51	1			
304.2853	15.73	1			
289.2359	19.07	1			
349.2313	2.74	1			
225.1129	14.48	1			
455.117	2.98	1			
188.165	13.47	1			
458.1497	12.86	1		Saccharides	Disaccharides
423.1021	11.48	1			
238.0939	11.15	1			
268.1052	9.72	1			
180.0889	10.21	1			
249.0879	11.89	1	5-OH-indole-acetic acid-hexoside	Indole derivatives	Alpha amino acids
520.3413	17.45	1			
429.1345	10.94	1		other aromatic compounds	Phenol ethers
409.1125	12.81	1			
237.1129	11.81	1			
501.6159	3.12	1			
308.114	10.35	1	1-hexosyl-indole-3-carboxaldehyde	Indole derivatives	Coumarin glycosides
443.1082	12.85	1			
772.2967	3.05	1		other compounds	Diterpenoids
448.2917	19.97	1			
389.2303	19.52	1			
471.1058	11.06	1	GSH + 3-methylsulfinylpropyl ITC	Peptides	Gamma-glutamyl peptides
443.2681	12.73	1			
251.0857	12.44	1	indole-3-ylmethyl cysteine	Indole derivatives	Alpha amino acids and derivatives
414.1627	11.71	1		Glycosides	O-glycosyl compounds
152.0573	10.03	1			
256.5652	11.81	1			
351.1564	13.14	1			
164.0206	12.71	1			
189.0488	11.85	1			
318.0346	12.94	1		Amino acid derivatives and peptides	Cysteine and derivatives
617.1502	12.98	1	Kaempferol-dihexoside	flavonoid glycosides	Flavonoid-3-O-glycosides
210.0413	17.29	1			
370.2961	19.69	1			
236.0518	11.81	1		other aromatic compounds	Benzene and substituted derivatives
202.127	11.71	1			
329.1603	19.96	1			
325.0474	12.98	1		other aromatic compounds	Benzenoids
420.115	12.1	1			
296.1175	10.54	1			
471.027	11.96	1			
364.2239	12.93	1			
360.1669	12.86	1		Cyanogenic glycosides	Cyanogenic glycosides
270.0431	13.07	1			
295.1299	11.82	1	Gamma-Glu-Phe	Peptides	Gamma-glutamyl amino acids
235.1187	12.22	1			
309.1192	12.53	1			
281.1004	11.33	1		Amino acid derivatives and peptides	Alpha amino acids and derivatives
391.13	10.89	1		Peptides	Gamma-glutamyl peptides
443.0969	12.16	1			
320.1354	10.48	1		Nucleotide derivatives	Purine nucleosides
454.2942	17.75	1	1-16:0-lysoPE	Lipids and lipid-like molecules	
384.1168	11.01	1		Nucleotide derivatives	Purine nucleosides
322.1888	12.41	1			
272.5316	13.25	1			
353.086	11.09	1			
321.1099	12.85	1			
348.1303	11.1	1		Glycosides	O-glycosyl compounds
308.1142	12.66	1			
273.0895	15.22	1			
314.1836	9.93	1			
496.3089	12.83	1			
496.1947	12.85	1		Glycosides	Glycosyl compounds
308.1352	10.55	1			
212.0546	10.76	1			
285.1683	9.54	1		Lipids and lipid-like molecules	Glycerolipids
392.0991	12.3	1		other aromatic compounds	Nitrobenzenes
458.1275	12.18	1			
384.1524	11.16	1		Glycosides	Hexoses
474.2957	12.78	1			
263.1398	14.24	1			
518.9847	10.76	1			
162.0556	13.15	1			
286.5655	12.28	1			
501.1501	12.86	1			
357.9849	12.13	1		other aromatic compounds	Benzenesulfonamides
480.31	18.06	1	1-18:0-lysoPE	Lipids and lipid-like molecules	
446.0562	12.19	1		Glucosinolates	Alkylglucosinolates
533.0001	12.33	1			
369.1195	12.81	1	5-O-Feruloylquinic acid	Glycosides	Phenolic glycosides
328.1265	9.55	1			
322.0936	12.26	1		Amino acid derivatives and peptides	Alpha amino acids and derivatives
244.228	17.67	1			
455.1615	12.79	1		Amino acid derivatives and peptides	N-acyl-alpha amino acids and derivatives
242.5313	11.64	1			
310.079	10.54	1			
503.1895	11.09	1		Peptides	Dipeptides
237.0723	12.34	1			
273.0821	2.96	1		other aromatic compounds	Benzenoids
292.1984	2.83	1			
336.2042	12.85	1			
302.1507	12.15	1			
464.1783	11.82	1		Glycosides	O-glycosyl compounds
277.1058	12.32	1			
258.171	11.22	1			
272.0495	12.14	1		other aromatic compounds	Benzenoids
320.0325	12.95	1			
154.1128	16.08	1			
291.0988	13.33	1		Amino acid derivatives and peptides	Alpha amino acids and derivatives
323.0964	13.08	1			
288.1679	9.88	1		other compounds	Morpholines
192.0519	14.16	1			
642.2248	11.73	1		other aromatic compounds	Methoxyphenols
417.0834	10.39	1			
178.0508	11.1	1			
531.1159	12.54	1			
469.1837	11.57	1		Glycosides	Monosaccharides
353.1066	13.49	1	Methoxycoumarin-hexoside	Coumarins and their glycosides	
226.0981	19.34	1			
300.0924	11.34	1			
341.039	15.29	1		Nucleotide derivatives	Pyrimidine ribonucleotides
492.2455	14.16	1		Lipids and lipid-like molecules	Alkyl glycosides
257.1655	13.42	1			
386.1317	10.52	1			
192.0665	10.56	1		other aromatic compounds	Benzene and substituted derivatives
423.1387	12.58	1	Cys Cys Pro Thr	Peptides	Peptides
483.0212	11.12	1			
520.0087	10.52	1			
415.2196	14.81	1			
280.1225	10.53	1			
210.0913	13.13	1			
209.0455	10.61	1			
529.1702	12.29	1			
262.0753	12.32	1			
295.6989	12.77	1			
345.6052	2.96	1			
364.7255	12.93	1			
166.0731	9.55	1			
272.1729	10.84	1			
324.0912	12.15	1		Glycosides	Hexoses
201.5417	13.67	1			
492.089	10.12	1			
300.091	11.75	1			
370.091	10.91	1		Glycosides	Hexoses
514.0183	12.17	1			
209.2021	13.76	1			
431.029	11.1	1			
241.5419	12.81	1			
180.0679	10.61	1			
271.179	11.65	1			
210.0926	11.16	1			
218.5751	14.9	1			
433.2312	10.23	1		Peptides	Oligopeptides
247.1451	12.3	1	Indol-3-ylmethyl amino derivative	Amino acid derivatives and peptides	Alpha amino acids
419.5798	11.57	1			
498.181	13.29	1		other aromatic compounds	Anisoles
258.1573	9.67	1			
292.5656	12.28	1			
535.1106	13.49	1	Kaempferol derivate	flavonoid glycosides	Flavonoid-3-O-glycosides
218.1754	14.79	1			
308.1174	11.11	1			
307.0939	12.24	1		Amino acid derivatives and peptides	Alpha amino acids and derivatives
243.0597	12.53	1			
515.0809	13.63	1		other aromatic compounds	Benzene and substituted derivatives
257.0674	11.81	1		other aromatic compounds	Benzene and substituted derivatives
388.0338	12.19	1			
271.0343	10.12	1			
325.5489	12.98	1			
360.0201	11.34	1			
309.1132	12.28	1		other aromatic compounds	Benzenoids
350.0369	10.54	1			
292.1224	11.1	1			
222.0293	2.83	1			
485.1673	12.22	1		Coumarins and their glycosides	Coumarins and derivatives
417.0133	11.23	1		Amino acid derivatives and peptides	Alpha hydroxy acids and derivatives
382.1149	13.11	1		Glycosides	O-glycosyl compounds
671.0489	12.86	1			
220.0836	11.16	1			
382.1878	10.12	1			
539.0059	13.25	1			
292.0048	16.55	1			
343.0369	15.29	1		other aromatic compounds	Benzoyl derivatives
236.5531	11.81	1			
273.0328	13.25	1			
388.1619	13.35	1			
357.1306	11.09	1		other aromatic compounds	Benzene and substituted derivatives
483.048	12.85	1			
487.1944	12.39	1		Saccharides	Disaccharides
388.1253	11.37	1		Amino acid derivatives and peptides	Amino acids
298.1156	10.61	1		Nucleotide derivatives	Purine nucleosides
312.1566	11.47	1		Nucleotide derivatives	Pyrimidine nucleosides
219.5468	12.86	1			
244.0707	12.36	1			
393.1012	10.53	1			
314.1064	11.36	1			
304.1037	11.85	1			
461.9663	10.54	1			
185.5488	10.91	1			
312.0944	10.53	1			
361.0493	13.65	1			
305.023	11.1	1		other aromatic compounds	Halophenols
248.0924	13.67	1			
322.0913	10.61	1		Glycosides	Hexoses
263.0448	11.63	1			
251.53	11.95	1			
343.702	11.91	1			
422.0241	11.8	1			
281.1139	11.61	1			
335.0775	11.64	1		Coumarins and their glycosides	Coumarins and derivatives
375.1451	13.81	1		other aromatic compounds	Furofuran lignans
368.0814	13.18	1			
344.1354	9.95	1		Cyanogenic glycosides	Cyanogenic glycosides
231.066	11.64	1			
422.026	3.41	-1			
315.0725	10.78	-1			
285.062	11.53	-1			
622.0212	12.07	-1			
285.062	11.74	-1			
388.0748	11.52	-1	pentyl GSL	Glucosinolates	Alkylglucosinolates
226.1081	12.68	-1			
467.0505	11.6	-1			
352.0865	13.66	-1			
307.0863	12.34	-1			
490.0465	12.06	-1		Coumarins and their glycosides	Coumarin glycosides
357.083	11.94	-1		Glycosides	Hexoses
418.8143	0.07	-1			
186.8553	12.06	-1			
438.0547	10.85	-1			
289.0834	13.13	-1			
549.1256	13.13	-1	Kaempferol-dipentoside	flavonoid glycosides	Flavonoid-3-O-glycosides
444.0282	10.71	-1			
467.1596	14.33	-1			
417.0833	13.42	-1			
371.0982	12.48	-1			
394.8907	2.73	-1			
224.056	10.42	-1			
477.152	12.75	-1			
206.0819	12.93	-1			
364.1043	11.83	-1			
445.1358	11.71	-1			
322.9411	2.6	-1			
647.0171	11.73	-1			
464.1059	13.72	-1			
621.0195	11.72	-1			
208.0612	10.38	-1	formyl tyrosine	Amino acid derivatives and peptides	
322.094	11.07	-1			
323.1355	11.07	-1			
551.0285	11.72	-1			
547.931	12.05	-1			
213.049	2.86	-1			
218.1028	10.63	-1			
338.037	9.92	-1		other compounds	Imidolactams
364.8708	2.77	-1			
211.0247	12.52	-1		other aromatic compounds	Methoxybenzoic acids and derivatives
289.1121	13.56	-1			
581.1192	12.23	-1			
229.0232	2.86	-1			
402.0501	12.16	-1			
338.0885	10.95	-1			
401.1093	11.61	-1			
341.0279	2.8	-1			
469.1223	13.33	-1			
178.0863	13.56	-1			
245.0932	13.24	-1		Amino acid derivatives and peptides	N-acyl-alpha amino acids
468.152	12.28	-1			
549.93	12.05	-1			
424.99	2.8	-1			
327.0732	12.52	-1			
325.0539	2.81	-1			
310.0023	9.9	-1			
495.1184	11.91	-1		Glycosides	Hexoses
264.0877	13.57	-1			
375.1201	12.77	-1			
279.1086	10.71	-1			
503.0536	10.95	-1			
196.9467	2.79	-1			
247.0369	2.89	-1			
203.082	11.37	-1			
409.0161	2.79	-1			
549.1477	10.61	-1			
436.0752	12.53	-1			
226.966	2.72	-1			
449.0773	13.51	-1			
484.1473	10.93	-1			
282.085	9.84	-1			
351.1308	12.26	-1		Glycosides	Hexoses
171.0555	12.75	-1			
520.1178	10.7	-1			
325.1259	2.91	-1			
439.1834	11.78	-1			
443.1208	12.46	-1			
357.1299	2.88	-1	1-OH-indole-3- carboxylic acid Gly derivative	other aromatic compounds	Benzene and substituted derivatives
435.0617	13.01	-1			
320.0986	10.14	-1			
375.0944	10.96	-1			
363.0765	12.99	-1			
489.0382	10.78	-1			
264.9351	2.79	-1			
319.0946	12.77	-1			
225.9845	12.66	-1			
323.1258	10.17	-1			
340.0601	13.08	-1			
249.0878	12.74	-1			
358.1141	2.91	-1			
267.9638	2.89	-1		other compounds	Azoles
168.027	2.91	-1			
301.0937	10.29	-1			
393.042	2.79	-1			
225.0882	10.63	-1			
187.9964	2.83	-1			
