# Supplementary material for: Correlations Between the Metabolome and the Endophytic Fungal Metagenome Suggests Importance of Various Metabolite Classes in Community Assembly in Horseradish (Armoracia rusticana, Brassicaceae) Roots
Source: Front Plant Sci. 2022 Jun 17;13:921008. doi: 10.3389/fpls.2022.921008 (PMC9247618; doi:10.3389/fpls.2022.921008)
Supplement: Supplementary file 14 [file Table_9.PDF]

Table S9. Compound IDs for indices along axis x in Figure 4 and Figure Sx. Names indicate MSI level 1-2 identification/annotation, MSI level annotation is marked as class_mz_rt, while features of unknown compounds are marked mz_rt.

Order	ID
1	386.1317_10.52
2	520.0087_10.52
3	Glycosides_414.1627_11.71
4	202.127_11.71
5	238.0939_11.15
6	Glycosides_384.1524_11.16
7	220.0836_11.16
8	210.0413_17.29
9	Glucosinolates_446.0562_12.19
10	other aromatic compounds_211.0247_12.52
11	5-OH-indole-acetic acid-hexoside
12	other aromatic compounds_392.0991_12.3
13	264.0877_13.57
14	Glycosides_469.1837_11.57
15	Amino acid derivatives and peptides_281.1004_11.33
16	other aromatic compounds_309.1132_12.28
17	Nucleotide derivatives_384.1168_11.01
18	Indole 3 carboxylic acid
19	166.0731_9.55
20	Saccharides_487.1944_12.39
21	Glycosides_351.1308_12.26
22	Amino acid derivatives and peptides_388.1253_11.37
23	other aromatic compounds_429.1345_10.94
24	1-hexosyl-indole-3-carboxaldehyde
25	489.1945_3.04
26	Cyanogenic glycosides_360.1669_12.86
27	443.1208_12.46
28	5-O-Feruloylquinic acid
29	other aromatic compounds_272.0495_12.14
30	308.1352_10.55
31	Glycosides_382.1149_13.11
32	449.0773_13.51
33	363.0765_12.99
34	Kaempferol derivate
35	Amino acid derivatives and peptides_291.0988_13.33
36	Amino acid derivatives and peptides_245.0932_13.24
37	287.056_13.51
38	417.0833_13.42
39	539.0059_13.25
40	Kaempferol-hexoside
41	Kaempferol-dihexoside
42	287.0559_12.86
43	671.0489_12.86
44	Kaempferol-dipentoside
45	other aromatic compounds_325.0474_12.98
46	325.5489_12.98
47	272.5316_13.25
48	273.0328_13.25
49	gluconasturtiin
50	N,N-(Dimethyl)thiobenzamide
51	pentyl GSL
52	Amino acid derivatives and peptides_307.0939_12.24
53	Cyanogenic glycosides_344.1354_9.95
54	Glycosides_496.1947_12.85
55	Peptides_503.1895_11.09
56	484.1473_10.93
57	other aromatic compounds_192.0665_10.56
58	formyl tyrosine
59	180.0679_10.61
60	224.056_10.42
61	327.0732_12.52
62	Lipids and lipid-like molecules_492.2455_14.16
63	Saccharides_458.1497_12.86
64	352.0865_13.66
65	285.062_11.53
66	304.1037_11.85
67	285.062_11.74
68	467.1596_14.33
69	531.1159_12.54
70	Glycosides_324.0912_12.15
71	Amino acid derivatives and peptides_417.0133_11.23
72	420.115_12.1
73	Glycosides_357.083_11.94
74	Saccharides_487.1668_3.05
75	flavonoid glycosides_793.2335_3.11
76	Lipids and lipid-like molecules_793.7291_3.09
77	sinigrin
78	350.0369_10.54
79	280.1225_10.53
80	393.1012_10.53
81	289.1121_13.56
82	417.0834_10.39
83	Glycosides_322.0913_10.61
84	325.1259_2.91
85	Nucleotide derivatives_341.039_15.29
86	other aromatic compounds_343.0369_15.29
87	Amino acid derivatives and peptides_318.0346_12.94
88	320.0325_12.95
89	Gamma-Glu-Phe
90	1-OH-indole-3- carboxylic acid Gly derivative
91	358.1141_2.91
92	322.1888_12.41
93	336.2042_12.85
94	272.1729_10.84
95	258.1573_9.67
96	307.0863_12.34
97	349.2313_2.74
98	other compounds_772.2967_3.05
99	314.1836_9.93
100	226.1081_12.68
101	other compounds_288.1679_9.88
102	277.1058_12.32
103	Nucleotide derivatives_298.1156_10.61
104	other aromatic compounds_357.9849_12.13
105	Cys Cys Pro Thr
106	Lipids and lipid-like molecules_285.1683_9.54
107	282.085_9.84
108	glucobrassicin
109	262.0753_12.32
110	glucoiberin
111	Indol-3-ylmethyl amino derivative
112	other compounds_204.0158_11.29
113	GSH + 3-methylsulfinylpropyl ITC
114	indole-3-ylmethyl cysteine
115	Amino acid derivatives and peptides_455.1615_12.79
116	182.0834_6.85
117	382.1878_10.12
118	271.0343_10.12
119	other compounds_338.037_9.92
120	422.026_3.41
121	461.9663_10.54
122	310.079_10.54
123	312.0944_10.53
124	401.1093_11.61
125	388.1619_13.35
126	Glycosides_370.091_10.91
127	185.5488_10.91
128	other aromatic compounds_515.0809_13.63
129	Nucleotide derivatives_320.1354_10.48
130	444.0282_10.71
131	Glycosides_464.1783_11.82
132	Coumarins and their glycosides_335.0775_11.64
133	other aromatic compounds_305.023_11.1
134	Glycosides_348.1303_11.1
135	503.0536_10.95
136	375.0944_10.96
137	419.5798_11.57
138	435.0617_13.01
139	581.1192_12.23
140	Glycosides_495.1184_11.91
141	Coumarins and their glycosides_485.1673_12.22
142	256.5652_11.81
143	other aromatic compounds_257.0674_11.81
144	263.1398_14.24
145	Amino acid derivatives and peptides_322.0936_12.26
146	other aromatic compounds_375.1451_13.81
147	279.1086_10.71
148	other compounds_178.1269_10.14
149	268.1052_9.72
150	210.0913_13.13
151	323.0964_13.08
152	192.0519_14.16
153	219.0291_2.89
154	351.1564_13.14
155	340.0601_13.08
156	164.0206_12.71
157	423.1021_11.48
158	225.9845_12.66
159	1-18:3-lysoPC
160	1-16:0-lysoPE
161	1-18:0-lysoPE
162	Methoxycoumarin-hexoside
163	268.2277_17.81
164	224.1646_16.59
165	212.1649_15.03
