# Supplementary material for: Correlations Between the Metabolome and the Endophytic Fungal Metagenome Suggests Importance of Various Metabolite Classes in Community Assembly in Horseradish (Armoracia rusticana, Brassicaceae) Roots
Source: Front Plant Sci. 2022 Jun 17;13:921008. doi: 10.3389/fpls.2022.921008 (PMC9247618; doi:10.3389/fpls.2022.921008)
Supplement: Supplementary file 13 [file Table_8.PDF]

Table S8. Statistically significant phenomena, after Benjamini Hochberg correction of p-values. Column legend (in order of appearance): set; data-mining experiment type. Can be one of p1cordf_c (correlation between fungal data and sPCA scores of chemical data), pc1cordf_f (correlation between chemical data and sPCA scores of fungal data), p2cordf (correlation between sPCA scores of fungal and sPCA scores of chemical data), pcm (statistical difference between accessions for sPCA scores of chemical data), pcrstats_t (statistical difference between sample types in sPCA scores of fungal community data), rawcordf (correlation between chemical data and fungal data), type_diversity (significant differences among diversity indices); mcol, ID of chemical in selected core chemical data or number of chemical sPCA PC; k, level of aggregation for fungal data (1 = phylum-level aggregates, 2 = order-level aggregates, 3 = genus-level data, 4 = diversity indices); fcol, ID of fungal feature or number of fungal sPCA PC; corv, Spearman correlation value; effect_size, difference between Q1 and Q4 subset of fungal abundance, binned along chemical data; pv_bhadj, p-value after Benjamini Hochberg correction; sigtxt, significance (***, p-adj < 0.001, **, p-adj < 0.01, *, p-adj < 0.05); abslowerCI, lower edge of 95% confidence interval for bootstrapped Spearman correlation value (n = 250); fungal_part, fungal taxon used in correlation in UNITE notation ("p__", phylum; "o__", order; "c__", class; "f__", family; "g__", genus) or sPCA principal component number; mzmed, m/z of chemical feature in correlation; rtmed, retention time of chemical feature in correlation; polarity, 1 or -1 denotes positive or negative; putative_identification, MSI level 2 identification supported manual comparison with references, as in Table 1; manual_class, manually assigned compound class based on Table 1 putative identifications or canopus_class and CSI:FingerID suggestions; canopus_class, "most specific class" suggestion in the Classyfire hierarchy by the canopus algorithm; id, compound id as plotted in Fig 3.: identified compounds appear as names, class annotated compounds as class_mz_rt, unknowns as mz_rt.

set	mcol	k	fcol	corv	effect_size	pv_bhadj	sigtxt	abslowerCI	fungal_part	mzmed	rtmed	polarity	putative_identification	manual_class	canopus_class	id
p1cordf_c	1	2	28	0.42459904203734	0.723459484017734	0.0358626153239792	*	0.224840178096139	o__Saccharomycetales	NA	NA	NA	NA	Glucosinolates	NA	sinigrin
p1cordf_c	1	2	31	-0.583213134145841	-2.05131272033939	0.000473375022300972	***	0.388753634505046	o__Xylariales	NA	NA	NA	NA	Glucosinolates	NA	sinigrin
p1cordf_c	1	3	8	-0.433049415635823	-1.44388540504217	0.029364712848756	*	0.221035931216462	f__Morosphaeriaceae	NA	NA	NA	NA	Glucosinolates	NA	sinigrin
p1cordf_f	6	2	2	-0.573855311355311	-1.49422938626309	0.000952136004935433	***	0.409010996441003	PC2_2	287.0559	12.86	1				287.0559_12.86
p1cordf_f	8	2	2	0.631043956043956	1.90634103026659	0.000147759186710128	***	0.457143144876418	PC2_2	204.0158	11.29	1		other compounds	Organonitrogen compounds	other compounds_204.0158_11.29
p1cordf_f	8	2	3	-0.42797619047619	-1.35264763840649	0.0358626153239792	*	0.200805116856377	PC2_3	204.0158	11.29	1		other compounds	Organonitrogen compounds	other compounds_204.0158_11.29
p1cordf_f	18	2	2	-0.547619047619048	-1.41562790106602	0.00216965856663657	**	0.341129654726207	PC2_2	449.1098	12.86	1	Kaempferol-hexoside	flavonoid glycosides		Kaempferol-hexoside
p1cordf_f	21	2	2	-0.560485347985348	-1.75552746139664	0.00136694095964666	**	0.363997290162821	PC2_2	287.056	13.51	1				287.056_13.51
p1cordf_f	26	2	2	-0.462454212454212	-1.08403191827347	0.0171238171269551	*	0.24388453981427	PC2_2	268.1052	9.72	1				268.1052_9.72
p1cordf_f	31	2	2	0.525091575091575	1.90190626388791	0.0038588887692747	**	0.328245728298681	PC2_2	471.1058	11.06	1	GSH + 3-methylsulfinylpropyl ITC	Peptides	Gamma-glutamyl peptides	GSH + 3-methylsulfinylpropyl ITC
p1cordf_f	35	2	2	0.421336996336996	1.26830705427791	0.0420196666616092	*	0.178947444023242	PC2_2	351.1564	13.14	1				351.1564_13.14
p1cordf_f	38	2	2	-0.548037408889029	-1.79813808660295	0.00153901796546587	**	0.368414635009528	PC2_2	617.1502	12.98	1	Kaempferol-dihexoside	flavonoid glycosides	Flavonoid-3-O-glycosides	Kaempferol-dihexoside
p1cordf_f	41	2	2	-0.515338827838828	-1.64400904322247	0.00498680589927144	**	0.301269028490381	PC2_2	325.0474	12.98	1		other aromatic compounds	Benzenoids	other aromatic compounds_325.0474_12.98
p1cordf_f	49	2	2	0.509478021978022	2.01100082204841	0.00570624326541637	**	0.288652504209666	PC2_2	322.1888	12.41	1				322.1888_12.41
p1cordf_f	50	2	2	-0.589194139194139	-1.80592666649151	0.000564600896553549	***	0.396584179316211	PC2_2	272.5316	13.25	1				272.5316_13.25
p1cordf_f	52	2	2	0.495628448844503	1.35151689512788	0.00686646996985805	**	0.292145680654599	PC2_2	314.1836	9.93	1				314.1836_9.93
p1cordf_f	55	2	2	0.470283882783883	1.55896368274024	0.0147693974645664	*	0.240982600560248	PC2_2	285.1683	9.54	1		Lipids and lipid-like molecules	Glycerolipids	Lipids and lipid-like molecules_285.1683_9.54
p1cordf_f	59	2	2	0.709418136796267	2.17405645656585	3.15897270631867E-07	***	0.548199666654001	PC2_2	357.9849	12.13	1		other aromatic compounds	Benzenesulfonamides	other aromatic compounds_357.9849_12.13
p1cordf_f	64	2	2	0.440384615384615	1.45038555379757	0.0271120014186002	*	0.2727434972443	PC2_2	455.1615	12.79	1		Amino acid derivatives and peptides	N-acyl-alpha amino acids and derivatives	Amino acid derivatives and peptides_455.1615_12.79
p1cordf_f	69	2	2	0.579166666666667	1.60274813541045	0.000763953611660005	***	0.330850163629655	PC2_2	277.1058	12.32	1				277.1058_12.32
p1cordf_f	84	2	2	0.593438352847334	2.26640093927982	0.000340782169989379	***	0.409686391531759	PC2_2	423.1387	12.58	1	Cys Cys Pro Thr	Peptides	Peptides	Cys Cys Pro Thr
p1cordf_f	88	2	2	0.599267399267399	1.8874706134634	0.000376615104503238	***	0.397829394784552	PC2_2	262.0753	12.32	1				262.0753_12.32
p1cordf_f	90	2	2	0.440521978021978	1.32741647807717	0.0271120014186002	*	0.254909969242933	PC2_2	272.1729	10.84	1				272.1729_10.84
p1cordf_f	94	2	2	0.465659340659341	1.44201292864708	0.0163904646822836	*	0.270710515749092	PC2_2	247.1451	12.3	1	Indol-3-ylmethyl amino derivative	Amino acid derivatives and peptides	Alpha amino acids	Indol-3-ylmethyl amino derivative
p1cordf_f	96	2	2	0.502701465201465	1.53523180159994	0.00670608808897105	**	0.335835462861821	PC2_2	258.1573	9.67	1				258.1573_9.67
p1cordf_f	97	2	2	-0.576810810715916	-1.51472211614146	0.000588933327795724	***	0.370296983069883	PC2_2	535.1106	13.49	1	Kaempferol derivate	flavonoid glycosides	Flavonoid-3-O-glycosides	Kaempferol derivate
p1cordf_f	98	2	2	-0.567673992673993	-1.8918638439081	0.0012365400354162	**	0.409076065166961	PC2_2	307.0939	12.24	1		Amino acid derivatives and peptides	Alpha amino acids and derivatives	Amino acid derivatives and peptides_307.0939_12.24
p1cordf_f	102	2	2	-0.514703693986262	-1.50112691910303	0.00422846804454719	**	0.28483780993357	PC2_2	325.5489	12.98	1				325.5489_12.98
p1cordf_f	107	2	2	-0.427838827838828	-1.18885749059868	0.0358626153239792	*	0.190489419328113	PC2_2	382.1149	13.11	1		Glycosides	O-glycosyl compounds	Glycosides_382.1149_13.11
p1cordf_f	108	2	2	-0.5625	-1.49041949617542	0.00135552693099767	**	0.408793372772463	PC2_2	671.0489	12.86	1				671.0489_12.86
p1cordf_f	111	2	2	-0.630318312839099	-1.83857568363807	8.49633678718511E-05	***	0.454781697143151	PC2_2	539.0059	13.25	1				539.0059_13.25
p1cordf_f	113	2	2	-0.565247252747253	-1.70034301086087	0.00128102054961322	**	0.355987091062945	PC2_2	273.0328	13.25	1				273.0328_13.25
p1cordf_f	117	2	2	0.473443223443223	1.3742195088329	0.0139882158053529	*	0.252480212881612	PC2_2	298.1156	10.61	1		Nucleotide derivatives	Purine nucleosides	Nucleotide derivatives_298.1156_10.61
p1cordf_f	131	2	2	-0.452518315018315	-1.40125187911564	0.0207889288667243	*	0.276932869474044	PC2_2	388.0748	11.52	-1	pentyl GSL	Glucosinolates	Alkylglucosinolates	pentyl GSL
p1cordf_f	132	2	3	-0.475239870953442	NA	0.0120455526272312	*	0.249394205539036	PC2_3	226.1081	12.68	-1				226.1081_12.68
p1cordf_f	134	2	2	0.438003663003663	1.01947082136404	0.0289909064544089	*	0.157442772171269	PC2_2	307.0863	12.34	-1				307.0863_12.34
p1cordf_f	136	2	2	-0.525549450549451	-1.56546153845265	0.0038588887692747	**	0.321262238295363	PC2_2	549.1256	13.13	-1	Kaempferol-dipentoside	flavonoid glycosides	Flavonoid-3-O-glycosides	Kaempferol-dipentoside
p1cordf_f	139	2	2	-0.529487179487179	-1.42282208654981	0.00367321407497801	**	0.294224210523379	PC2_2	417.0833	13.42	-1				417.0833_13.42
p1cordf_f	155	2	2	0.445558608058608	1.28636953045502	0.0245912679295426	*	0.238571072994668	PC2_2	282.085	9.84	-1				282.085_9.84
p1cordf_f	163	2	2	-0.473901098901099	-1.29663708013814	0.0139857142857143	*	0.267662299174603	PC2_2	225.9845	12.66	-1				225.9845_12.66
p1cordf_f	6	3	1	0.421703296703297	1.54986768602795	0.0416886283329864	*	0.202589905303669	PC3_1	287.0559	12.86	1				287.0559_12.86
p1cordf_f	6	3	2	0.479166666666667	1.10024712582319	0.0122746095556082	*	0.284972212942699	PC3_2	287.0559	12.86	1				287.0559_12.86
p1cordf_f	8	3	1	-0.505631868131868	-1.30770923434303	0.00617718818668201	**	0.297742145988159	PC3_1	204.0158	11.29	1		other compounds	Organonitrogen compounds	other compounds_204.0158_11.29
p1cordf_f	8	3	2	-0.465338827838828	-1.41606991270087	0.0164649477444857	*	0.254225427526338	PC3_2	204.0158	11.29	1		other compounds	Organonitrogen compounds	other compounds_204.0158_11.29
p1cordf_f	18	3	1	0.42220695970696	1.40142221969102	0.0411753453460322	*	0.213116240045079	PC3_1	449.1098	12.86	1	Kaempferol-hexoside	flavonoid glycosides		Kaempferol-hexoside
p1cordf_f	18	3	2	0.428113553113553	1.05270323394413	0.0358626153239792	*	0.200959545284357	PC3_2	449.1098	12.86	1	Kaempferol-hexoside	flavonoid glycosides		Kaempferol-hexoside
p1cordf_f	21	3	2	0.471428571428571	1.24283101892837	0.0143730749980326	*	0.228658205099405	PC3_2	287.056	13.51	1				287.056_13.51
p1cordf_f	24	3	2	0.52489153954377	1.45537765277603	0.00335559367078998	**	0.333555342269886	PC3_2	423.1021	11.48	1				423.1021_11.48
p1cordf_f	26	3	2	0.525	1.57633496746559	0.0038588887692747	**	0.253047889469895	PC3_2	268.1052	9.72	1				268.1052_9.72
p1cordf_f	31	3	1	-0.468772893772894	-1.40755462676309	0.0153242129227423	*	0.278749263398151	PC3_1	471.1058	11.06	1	GSH + 3-methylsulfinylpropyl ITC	Peptides	Gamma-glutamyl peptides	GSH + 3-methylsulfinylpropyl ITC
p1cordf_f	31	3	2	-0.47220695970696	-1.24429312952922	0.0141685626570504	*	0.248559710469993	PC3_2	471.1058	11.06	1	GSH + 3-methylsulfinylpropyl ITC	Peptides	Gamma-glutamyl peptides	GSH + 3-methylsulfinylpropyl ITC
p1cordf_f	38	3	2	0.453897137130666	1.34970082330102	0.0183763052677789	*	0.20114880953069	PC3_2	617.1502	12.98	1	Kaempferol-dihexoside	flavonoid glycosides	Flavonoid-3-O-glycosides	Kaempferol-dihexoside
p1cordf_f	41	3	1	0.417078754578755	0.982444770663499	0.0461878660292202	*	0.193545007936741	PC3_1	325.0474	12.98	1		other aromatic compounds	Benzenoids	other aromatic compounds_325.0474_12.98
p1cordf_f	41	3	2	0.446382783882784	1.35907009477005	0.0241500287245895	*	0.253645390993915	PC3_2	325.0474	12.98	1		other aromatic compounds	Benzenoids	other aromatic compounds_325.0474_12.98
p1cordf_f	50	3	2	0.522527472527472	1.44218820912678	0.00409419606547673	**	0.315228245145112	PC3_2	272.5316	13.25	1				272.5316_13.25
p1cordf_f	55	3	1	-0.423855311355311	-0.855648644188217	0.0396555855846422	*	0.192421181908198	PC3_1	285.1683	9.54	1		Lipids and lipid-like molecules	Glycerolipids	Lipids and lipid-like molecules_285.1683_9.54
p1cordf_f	59	3	1	-0.500854997998686	-1.60476374470082	0.0060066267852576	**	0.286110914833061	PC3_1	357.9849	12.13	1		other aromatic compounds	Benzenesulfonamides	other aromatic compounds_357.9849_12.13
p1cordf_f	59	3	2	-0.551754335443334	-1.40955036987738	0.00136694095964666	**	0.316791748605009	PC3_2	357.9849	12.13	1		other aromatic compounds	Benzenesulfonamides	other aromatic compounds_357.9849_12.13
p1cordf_f	64	3	2	-0.483150183150183	-1.32960192619785	0.0113365433480006	*	0.294479398329851	PC3_2	455.1615	12.79	1		Amino acid derivatives and peptides	N-acyl-alpha amino acids and derivatives	Amino acid derivatives and peptides_455.1615_12.79
p1cordf_f	69	3	1	-0.509935897435897	-1.47958681457489	0.00568677355438378	**	0.304600021169395	PC3_1	277.1058	12.32	1				277.1058_12.32
p1cordf_f	69	3	2	-0.538003663003663	-1.55781023610827	0.00281385987969	**	0.331323846535642	PC3_2	277.1058	12.32	1				277.1058_12.32
p1cordf_f	84	3	1	-0.492848593837435	-1.36606638205388	0.00758877312583314	**	0.278810556123274	PC3_1	423.1387	12.58	1	Cys Cys Pro Thr	Peptides	Peptides	Cys Cys Pro Thr
p1cordf_f	84	3	2	-0.431422880343685	-1.57627118804203	0.0303705030061706	*	0.20204346908216	PC3_2	423.1387	12.58	1	Cys Cys Pro Thr	Peptides	Peptides	Cys Cys Pro Thr
p1cordf_f	88	3	2	-0.508608058608059	-1.43437757413208	0.00570624326541637	**	0.326149514904065	PC3_2	262.0753	12.32	1				262.0753_12.32
p1cordf_f	94	3	2	-0.420375457875458	-1.3755137030585	0.0431807795346817	*	0.206639170444501	PC3_2	247.1451	12.3	1	Indol-3-ylmethyl amino derivative	Amino acid derivatives and peptides	Alpha amino acids	Indol-3-ylmethyl amino derivative
p1cordf_f	96	3	1	-0.419917582417582	-0.763840308366856	0.0434793182003944	*	0.20700368926381	PC3_1	258.1573	9.67	1				258.1573_9.67
p1cordf_f	97	3	2	0.441179537296831	1.02550031362705	0.0247967954735852	*	0.214707343902745	PC3_2	535.1106	13.49	1	Kaempferol derivate	flavonoid glycosides	Flavonoid-3-O-glycosides	Kaempferol derivate
p1cordf_f	98	3	2	0.461217948717949	1.38224716386222	0.0172435710971282	*	0.269454127677542	PC3_2	307.0939	12.24	1		Amino acid derivatives and peptides	Alpha amino acids and derivatives	Amino acid derivatives and peptides_307.0939_12.24
p1cordf_f	102	3	1	0.415961721200801	1.22536660153233	0.0441384702816958	*	0.183101886165457	PC3_1	325.5489	12.98	1				325.5489_12.98
p1cordf_f	102	3	2	0.456964937815399	1.30099021864492	0.0172382148529493	*	0.249025592796539	PC3_2	325.5489	12.98	1				325.5489_12.98
p1cordf_f	108	3	2	0.464423076923077	1.14837401663533	0.0165514760376071	*	0.261875944536678	PC3_2	671.0489	12.86	1				671.0489_12.86
p1cordf_f	111	3	2	0.489077182504449	1.41692341906361	0.00842384927234428	**	0.302117502640713	PC3_2	539.0059	13.25	1				539.0059_13.25
p1cordf_f	113	3	1	0.457417582417582	1.64519378161631	0.0184938782162769	*	0.234371909406466	PC3_1	273.0328	13.25	1				273.0328_13.25
p1cordf_f	113	3	2	0.448397435897436	1.24715382370612	0.0232518803557677	*	0.199838730229814	PC3_2	273.0328	13.25	1				273.0328_13.25
p1cordf_f	124	3	4	0.46735347985348	1.81615169742888	0.0156211425903931	*	0.269202870427042	PC3_4	322.0913	10.61	1		Glycosides	Hexoses	Glycosides_322.0913_10.61
p1cordf_f	132	3	1	-0.477867175523781	NA	0.0113365433480006	*	0.236651691937812	PC3_1	226.1081	12.68	-1				226.1081_12.68
p1cordf_f	136	3	1	0.423214285714286	1.62165136949157	0.0403313882430335	*	0.206767174678426	PC3_1	549.1256	13.13	-1	Kaempferol-dipentoside	flavonoid glycosides	Flavonoid-3-O-glycosides	Kaempferol-dipentoside
p1cordf_f	136	3	2	0.425778388278388	1.17085241981053	0.0376904452264492	*	0.209899298337611	PC3_2	549.1256	13.13	-1	Kaempferol-dipentoside	flavonoid glycosides	Flavonoid-3-O-glycosides	Kaempferol-dipentoside
p1cordf_f	139	3	2	0.442628205128205	1.03917516744106	0.0257566659231812	*	0.204464388052174	PC3_2	417.0833	13.42	-1				417.0833_13.42
p1cordf_f	155	3	1	-0.414331501831502	-1.2202435370802	0.0494857527072041	*	0.183508232727133	PC3_1	282.085	9.84	-1				282.085_9.84
p1cordf_f	163	3	2	0.447435897435897	1.23806144880946	0.02370498877632	*	0.219806224918119	PC3_2	225.9845	12.66	-1				225.9845_12.66
p2cordf	1	2	2	0.519642857142857	1.55097372896247	0.00432907864950655	**	0.337645265161967	PC2_2	NA	NA	NA	NA	Glucosinolates	NA	sinigrin
p2cordf	1	3	1	-0.462225274725275	-1.307114963146	0.0171238171269551	*	0.231088408013996	PC3_1	NA	NA	NA	NA	Glucosinolates	NA	sinigrin
pcm	5	NA	NA	NA	2.22711460709871	0.0332571494056835	*	NA	PCNA_NA	268.2277	17.81	1				268.2277_17.81
pcrstats_t	NA	1	1	NA	9.83662348948587	1.28425032037887E-18	***	NA	PC1_1	NA	NA	NA	NA	NA	NA	NA
pcrstats_t	NA	2	1	NA	6.77796858573296	2.9252737742082E-18	***	NA	PC2_1	NA	NA	NA	NA	NA	NA	NA
pcrstats_t	NA	3	1	NA	4.6962424435269	8.16495047678345E-07	***	NA	PC3_1	NA	NA	NA	NA	NA	NA	NA
pcrstats_t	NA	3	3	NA	4.42946141891099	0.00137346913181165	**	NA	PC3_3	NA	NA	NA	NA	NA	NA	NA
rawcordf	6	2	31	0.50878036615196	1.47849310505124	0.00498680589927144	**	0.304818132474846	o__Xylariales	287.0559	12.86	1				287.0559_12.86
rawcordf	8	2	1	-0.438677625518478	-1.41199916526176	0.0257566659231812	*	0.252717276439803	c__Agaricomycetes	204.0158	11.29	1		other compounds	Organonitrogen compounds	other compounds_204.0158_11.29
rawcordf	8	2	9	0.534394139734059	1.67348020957354	0.00253381383234806	**	0.356658601420701	o__Capnodiales	204.0158	11.29	1		other compounds	Organonitrogen compounds	other compounds_204.0158_11.29
rawcordf	8	2	28	0.457385552050685	1.28212254805535	0.0171927972293665	*	0.196362634895196	o__Saccharomycetales	204.0158	11.29	1		other compounds	Organonitrogen compounds	other compounds_204.0158_11.29
rawcordf	8	2	29	-0.527438815129414	-1.47659410447496	0.00304594361070646	**	0.334508590957943	o__Sordariales	204.0158	11.29	1		other compounds	Organonitrogen compounds	other compounds_204.0158_11.29
rawcordf	8	2	31	-0.592577330426831	-2.00710343636237	0.000340782169989379	***	0.416991639891764	o__Xylariales	204.0158	11.29	1		other compounds	Organonitrogen compounds	other compounds_204.0158_11.29
rawcordf	17	2	31	-0.50410971568418	-1.80355695913705	0.00568677355438378	**	0.305532310768732	o__Xylariales	182.0834	6.85	1				182.0834_6.85
rawcordf	18	2	31	0.510268563604929	1.51013574536779	0.00482075608064268	**	0.304731492905305	o__Xylariales	449.1098	12.86	1	Kaempferol-hexoside	flavonoid glycosides		Kaempferol-hexoside
rawcordf	20	2	28	-0.460155920564382	-1.49140712775409	0.0164649477444857	*	0.269299977867918	o__Saccharomycetales	518.3259	16.79	1	1-18:3-lysoPC	Lipids and lipid-like molecules	Lysophosphatidylcholines	1-18:3-lysoPC
rawcordf	21	2	31	0.438903771898707	1.38395598309207	0.0257566659231812	*	0.232285269722273	o__Xylariales	287.056	13.51	1				287.056_13.51
rawcordf	23	2	31	0.466309500378767	1.46599001268831	0.0144261836998913	*	0.273653255431631	o__Xylariales	458.1497	12.86	1		Saccharides	Disaccharides	Saccharides_458.1497_12.86
rawcordf	26	2	28	-0.503749157341902	-1.60875937929945	0.00568677355438378	**	0.296794491060574	o__Saccharomycetales	268.1052	9.72	1				268.1052_9.72
rawcordf	31	2	9	0.491740599511937	1.85237874655199	0.00775474955496497	**	0.297307719239869	o__Capnodiales	471.1058	11.06	1	GSH + 3-methylsulfinylpropyl ITC	Peptides	Gamma-glutamyl peptides	GSH + 3-methylsulfinylpropyl ITC
rawcordf	31	2	28	0.478426615390254	1.25929858025224	0.0113365433480006	*	0.248492897242371	o__Saccharomycetales	471.1058	11.06	1	GSH + 3-methylsulfinylpropyl ITC	Peptides	Gamma-glutamyl peptides	GSH + 3-methylsulfinylpropyl ITC
rawcordf	31	2	29	-0.463861352258749	-1.53752022399535	0.0153242129227423	*	0.313907884985916	o__Sordariales	471.1058	11.06	1	GSH + 3-methylsulfinylpropyl ITC	Peptides	Gamma-glutamyl peptides	GSH + 3-methylsulfinylpropyl ITC
rawcordf	31	2	31	-0.614350803931039	-2.06182734315114	0.000147759186710128	***	0.445439310271052	o__Xylariales	471.1058	11.06	1	GSH + 3-methylsulfinylpropyl ITC	Peptides	Gamma-glutamyl peptides	GSH + 3-methylsulfinylpropyl ITC
rawcordf	32	2	29	-0.458664346831438	-1.13123895715248	0.0168594418576599	*	0.255820743372442	o__Sordariales	251.0857	12.44	1	indole-3-ylmethyl cysteine	Indole derivatives	Alpha amino acids and derivatives	indole-3-ylmethyl cysteine
rawcordf	35	2	28	0.411640128163351	1.2346041053503	0.0489845699835259	*	0.207395043322044	o__Saccharomycetales	351.1564	13.14	1				351.1564_13.14
rawcordf	37	2	28	0.475930994167172	1.0507969023494	0.0119405235046999	*	0.250951276511714	o__Saccharomycetales	318.0346	12.94	1		Amino acid derivatives and peptides	Cysteine and derivatives	Amino acid derivatives and peptides_318.0346_12.94
rawcordf	38	2	31	0.480693279781874	1.60877826961394	0.0106675789679842	*	0.288209172903474	o__Xylariales	617.1502	12.98	1	Kaempferol-dihexoside	flavonoid glycosides	Flavonoid-3-O-glycosides	Kaempferol-dihexoside
rawcordf	39	2	28	-0.475473082016147	-1.26375623073327	0.0120455526272312	*	0.280991303915113	o__Saccharomycetales	210.0413	17.29	1				210.0413_17.29
rawcordf	39	2	31	0.45163358395795	1.26076955373705	0.0191550300538583	*	0.235662866816025	o__Xylariales	210.0413	17.29	1				210.0413_17.29
rawcordf	41	2	31	0.481145684217597	1.54260009546764	0.0106455591907138	*	0.297844611308917	o__Xylariales	325.0474	12.98	1		other aromatic compounds	Benzenoids	other aromatic compounds_325.0474_12.98
rawcordf	42	2	31	0.453259153483501	1.18712134850615	0.0184938782162769	*	0.206352523778861	o__Xylariales	420.115	12.1	1				420.115_12.1
rawcordf	44	2	9	0.435350009298793	1.47203054177856	0.0280017946188769	*	0.236437680812337	o__Capnodiales	295.1299	11.82	1	Gamma-Glu-Phe	Peptides	Gamma-glutamyl amino acids	Gamma-Glu-Phe
rawcordf	49	2	9	0.508247954584481	1.76874403772444	0.00502602357696907	**	0.344881413074318	o__Capnodiales	322.1888	12.41	1				322.1888_12.41
rawcordf	50	2	31	0.485610276576504	1.81844115888104	0.00919737613061524	**	0.264109453449285	o__Xylariales	272.5316	13.25	1				272.5316_13.25
rawcordf	52	2	9	0.421605192510195	1.48585736541337	0.0384946297352834	*	0.201137305631044	o__Capnodiales	314.1836	9.93	1				314.1836_9.93
rawcordf	52	2	31	-0.471530707678733	-1.31161923952442	0.0133335718565073	*	0.25512742232148	o__Xylariales	314.1836	9.93	1				314.1836_9.93
rawcordf	54	2	31	0.53614030394116	1.75466451414884	0.00239220302718646	**	0.384689985795581	o__Xylariales	308.1352	10.55	1				308.1352_10.55
rawcordf	55	2	9	0.458771679533485	1.46665570034659	0.0168594418576599	*	0.269616416791783	o__Capnodiales	285.1683	9.54	1		Lipids and lipid-like molecules	Glycerolipids	Lipids and lipid-like molecules_285.1683_9.54
rawcordf	55	2	31	-0.440208806588234	-1.24222741451773	0.0254071466662875	*	0.257891684251995	o__Xylariales	285.1683	9.54	1		Lipids and lipid-like molecules	Glycerolipids	Lipids and lipid-like molecules_285.1683_9.54
rawcordf	59	2	1	-0.425389341741296	-1.42402561367119	0.035335290940394	*	0.217490019757968	c__Agaricomycetes	357.9849	12.13	1		other aromatic compounds	Benzenesulfonamides	other aromatic compounds_357.9849_12.13
rawcordf	59	2	9	0.518012021133822	1.69952861398197	0.0038588887692747	**	0.333320248140518	o__Capnodiales	357.9849	12.13	1		other aromatic compounds	Benzenesulfonamides	other aromatic compounds_357.9849_12.13
rawcordf	59	2	13	0.467180899214643	1.5210621618136	0.0141685626570504	*	0.270514938799587	o__Eurotiales	357.9849	12.13	1		other aromatic compounds	Benzenesulfonamides	other aromatic compounds_357.9849_12.13
rawcordf	59	2	28	0.499952479575584	1.27117040814909	0.00616206548599886	**	0.248383970302101	o__Saccharomycetales	357.9849	12.13	1		other aromatic compounds	Benzenesulfonamides	other aromatic compounds_357.9849_12.13
rawcordf	59	2	29	-0.467369611860715	-1.42012778755539	0.0141685626570504	*	0.209102665397871	o__Sordariales	357.9849	12.13	1		other aromatic compounds	Benzenesulfonamides	other aromatic compounds_357.9849_12.13
rawcordf	59	2	31	-0.576209040942587	-2.25475735276324	0.000588933327795724	***	0.428610426640157	o__Xylariales	357.9849	12.13	1		other aromatic compounds	Benzenesulfonamides	other aromatic compounds_357.9849_12.13
rawcordf	62	2	31	0.4296769476903	1.44397464294303	0.0319790089430781	*	0.197951818400975	o__Xylariales	369.1195	12.81	1	5-O-Feruloylquinic acid	Glycosides	Phenolic glycosides	5-O-Feruloylquinic acid
rawcordf	64	2	28	0.502352525281277	1.39164901430393	0.00570624326541637	**	0.291861112682566	o__Saccharomycetales	455.1615	12.79	1		Amino acid derivatives and peptides	N-acyl-alpha amino acids and derivatives	Amino acid derivatives and peptides_455.1615_12.79
rawcordf	64	2	31	-0.477367952221599	-0.960678980953774	0.0113802418002152	*	0.2382814439491	o__Xylariales	455.1615	12.79	1		Amino acid derivatives and peptides	N-acyl-alpha amino acids and derivatives	Amino acid derivatives and peptides_455.1615_12.79
rawcordf	66	2	31	0.486571881084576	1.79512234480387	0.0089364285956273	**	0.25915375727267	o__Xylariales	503.1895	11.09	1		Peptides	Dipeptides	Peptides_503.1895_11.09
rawcordf	69	2	28	0.420958640436697	0.981721907141417	0.0391828231596119	*	0.203720388695721	o__Saccharomycetales	277.1058	12.32	1				277.1058_12.32
rawcordf	69	2	31	-0.507017424553828	-1.54314245890301	0.00522511967667747	**	0.31115019081429	o__Xylariales	277.1058	12.32	1				277.1058_12.32
rawcordf	70	2	31	0.424182064787029	1.31818393513565	0.0358626153239792	*	0.234237024845631	o__Xylariales	272.0495	12.14	1		other aromatic compounds	Benzenoids	other aromatic compounds_272.0495_12.14
rawcordf	71	2	28	0.450196331279602	1.05523900305661	0.0198459044374519	*	0.244005507146211	o__Saccharomycetales	320.0325	12.95	1				320.0325_12.95
rawcordf	77	2	31	0.610344118480738	1.71892336260011	0.000152635332482008	***	0.450105006283633	o__Xylariales	531.1159	12.54	1				531.1159_12.54
rawcordf	84	2	28	0.433198217375263	1.32205963427207	0.029364712848756	*	0.166745971188426	o__Saccharomycetales	423.1387	12.58	1	Cys Cys Pro Thr	Peptides	Peptides	Cys Cys Pro Thr
rawcordf	84	2	29	-0.439151228637526	-0.831370043824843	0.0257566659231812	*	0.230316217811277	o__Sordariales	423.1387	12.58	1	Cys Cys Pro Thr	Peptides	Peptides	Cys Cys Pro Thr
rawcordf	84	2	31	-0.614768870011426	-3.03021734636255	0.000147759186710128	***	0.442748662214565	o__Xylariales	423.1387	12.58	1	Cys Cys Pro Thr	Peptides	Peptides	Cys Cys Pro Thr
rawcordf	88	2	9	0.554152596637812	1.35289978676933	0.00135552693099767	**	0.34169000721666	o__Capnodiales	262.0753	12.32	1				262.0753_12.32
rawcordf	88	2	13	0.42536579210553	1.15320350382665	0.035335290940394	*	0.197780268109645	o__Eurotiales	262.0753	12.32	1				262.0753_12.32
rawcordf	88	2	28	0.536970683898719	1.68465188211924	0.00236178465198178	**	0.305861514036747	o__Saccharomycetales	262.0753	12.32	1				262.0753_12.32
rawcordf	88	2	31	-0.570872542958913	-1.64928784998141	0.000748703216246641	***	0.360101340587463	o__Xylariales	262.0753	12.32	1				262.0753_12.32
rawcordf	90	2	9	0.451033141371876	1.20798083607553	0.0194698031104223	*	0.238829066254782	o__Capnodiales	272.1729	10.84	1				272.1729_10.84
rawcordf	90	2	28	0.414227331816639	1.01753772504667	0.046105813438408	*	0.207579117184775	o__Saccharomycetales	272.1729	10.84	1				272.1729_10.84
rawcordf	90	2	31	-0.442910457349009	-1.27399820695587	0.0239301397861397	*	0.23280691157126	o__Xylariales	272.1729	10.84	1				272.1729_10.84
rawcordf	91	2	28	-0.414914200043175	-1.47193497140371	0.0455216550554335	*	0.199610689056982	o__Saccharomycetales	324.0912	12.15	1		Glycosides	Hexoses	Glycosides_324.0912_12.15
rawcordf	91	2	31	0.530988851219344	1.50922972006375	0.00281385987969	**	0.33874140419376	o__Xylariales	324.0912	12.15	1		Glycosides	Hexoses	Glycosides_324.0912_12.15
rawcordf	94	2	9	0.442378799877533	1.0592239135324	0.0241500287245895	*	0.196540227596846	o__Capnodiales	247.1451	12.3	1	Indol-3-ylmethyl amino derivative	Amino acid derivatives and peptides	Alpha amino acids	Indol-3-ylmethyl amino derivative
rawcordf	96	2	9	0.520473929076491	1.76623021833297	0.0038588887692747	**	0.346071118936997	o__Capnodiales	258.1573	9.67	1				258.1573_9.67
rawcordf	96	2	28	0.440488593677886	1.01564983552084	0.0252859301898269	*	0.203545338735467	o__Saccharomycetales	258.1573	9.67	1				258.1573_9.67
rawcordf	96	2	31	-0.454243653337003	-1.39122878629219	0.0182537595104667	*	0.267964630455975	o__Xylariales	258.1573	9.67	1				258.1573_9.67
rawcordf	97	2	31	0.419496352685653	1.37289990566306	0.0404928599429265	*	0.202359685747004	o__Xylariales	535.1106	13.49	1	Kaempferol derivate	flavonoid glycosides	Flavonoid-3-O-glycosides	Kaempferol derivate
rawcordf	98	2	28	-0.492690578894663	-1.62181722421099	0.00758877312583314	**	0.316824169995973	o__Saccharomycetales	307.0939	12.24	1		Amino acid derivatives and peptides	Alpha amino acids and derivatives	Amino acid derivatives and peptides_307.0939_12.24
rawcordf	98	2	31	0.513382330583449	1.83863571767697	0.00432907864950655	**	0.312309208381553	o__Xylariales	307.0939	12.24	1		Amino acid derivatives and peptides	Alpha amino acids and derivatives	Amino acid derivatives and peptides_307.0939_12.24
rawcordf	101	2	31	-0.51716118168367	-1.81551445066207	0.00392018228543842	**	0.304966019205926	o__Xylariales	271.0343	10.12	1				271.0343_10.12
rawcordf	102	2	31	0.455485215907626	1.2115002194186	0.0177585603496793	*	0.247041044568727	o__Xylariales	325.5489	12.98	1				325.5489_12.98
rawcordf	104	2	31	-0.594507344739992	-2.0033022415893	0.000340782169989379	***	0.417899975706928	o__Xylariales	350.0369	10.54	1				350.0369_10.54
rawcordf	106	2	31	0.513542598001461	1.98717142030896	0.00432907864950655	**	0.306941263570288	o__Xylariales	417.0133	11.23	1		Amino acid derivatives and peptides	Alpha hydroxy acids and derivatives	Amino acid derivatives and peptides_417.0133_11.23
rawcordf	107	2	31	0.590287795883801	1.79545966458091	0.000361340756853175	***	0.380939087258355	o__Xylariales	382.1149	13.11	1		Glycosides	O-glycosyl compounds	Glycosides_382.1149_13.11
rawcordf	108	2	31	0.479978021600652	1.59988498055238	0.0108238383838384	*	0.23535723693928	o__Xylariales	671.0489	12.86	1				671.0489_12.86
rawcordf	110	2	31	-0.443322573566754	-1.58152181360199	0.02370498877632	*	0.265463387426818	o__Xylariales	382.1878	10.12	1				382.1878_10.12
rawcordf	111	2	31	0.458790396807909	1.52061265663784	0.0168594418576599	*	0.25101351889591	o__Xylariales	539.0059	13.25	1				539.0059_13.25
rawcordf	113	2	29	0.457084640335912	0.979947545286479	0.0172382148529493	*	0.166564978733299	o__Sordariales	273.0328	13.25	1				273.0328_13.25
rawcordf	113	2	31	0.529340386348363	1.83654318384085	0.00290862676677786	**	0.324772448570557	o__Xylariales	273.0328	13.25	1				273.0328_13.25
rawcordf	117	2	9	0.53929368756419	1.42982263095288	0.00216965856663657	**	0.320173041710284	o__Capnodiales	298.1156	10.61	1		Nucleotide derivatives	Purine nucleosides	Nucleotide derivatives_298.1156_10.61
rawcordf	117	2	31	-0.452938618647477	-1.07060559324573	0.0184938782162769	*	0.267968870984323	o__Xylariales	298.1156	10.61	1		Nucleotide derivatives	Purine nucleosides	Nucleotide derivatives_298.1156_10.61
rawcordf	118	2	31	-0.489959931439248	-1.79315821360183	0.00821352544676554	**	0.299941492751994	o__Xylariales	393.1012	10.53	1				393.1012_10.53
rawcordf	131	2	28	-0.463750530949923	-1.55819244930969	0.0153242129227423	*	0.266992435681786	o__Saccharomycetales	388.0748	11.52	-1	pentyl GSL	Glucosinolates	Alkylglucosinolates	pentyl GSL
rawcordf	131	2	31	0.496943472564499	1.7461051400475	0.00670608808897105	**	0.247895397725302	o__Xylariales	388.0748	11.52	-1	pentyl GSL	Glucosinolates	Alkylglucosinolates	pentyl GSL
rawcordf	132	2	1	-0.43384069323094	NA	0.0291069500947835	*	0.230576594313292	c__Agaricomycetes	226.1081	12.68	-1				226.1081_12.68
rawcordf	133	2	31	0.458616664314189	1.42000170880539	0.0168594418576599	*	0.240816001442567	o__Xylariales	352.0865	13.66	-1				352.0865_13.66
rawcordf	134	2	9	0.425459333305258	1.4705033569105	0.035335290940394	*	0.156152320085261	o__Capnodiales	307.0863	12.34	-1				307.0863_12.34
rawcordf	135	2	31	0.43913272535301	1.54128613626336	0.0257566659231812	*	0.235969875188923	o__Xylariales	357.083	11.94	-1		Glycosides	Hexoses	Glycosides_357.083_11.94
rawcordf	136	2	31	0.486800834538879	1.65212641497125	0.0089364285956273	**	0.291372945508839	o__Xylariales	549.1256	13.13	-1	Kaempferol-dipentoside	flavonoid glycosides	Flavonoid-3-O-glycosides	Kaempferol-dipentoside
rawcordf	138	2	31	0.497607437581977	1.51362643928118	0.00663821899974662	**	0.303030372192832	o__Xylariales	467.1596	14.33	-1				467.1596_14.33
rawcordf	139	2	2	-0.435604803197074	-1.44069016185667	0.0278862893808159	*	0.155307340965093	c__Laboulbeniomycetes	417.0833	13.42	-1				417.0833_13.42
rawcordf	142	2	31	-0.462714931146212	-1.68442031611931	0.0156211425903931	*	0.276373378864371	o__Xylariales	338.037	9.92	-1		other compounds	Imidolactams	other compounds_338.037_9.92
rawcordf	153	2	31	0.453602583664955	1.36414308595728	0.0184642740876054	*	0.22121987773154	o__Xylariales	449.0773	13.51	-1				449.0773_13.51
rawcordf	154	2	31	0.543100488951968	1.69032813343203	0.00190860428299149	**	0.339891326161696	o__Xylariales	484.1473	10.93	-1				484.1473_10.93
rawcordf	155	2	9	0.517909679744833	1.55779434668956	0.0038588887692747	**	0.352170904644003	o__Capnodiales	282.085	9.84	-1				282.085_9.84
rawcordf	155	2	29	-0.428283614663857	-1.2886659343365	0.0332571494056835	*	0.215794332775341	o__Sordariales	282.085	9.84	-1				282.085_9.84
rawcordf	159	2	32	-0.431905857827634	-1.06072046456306	0.0300257124177767	*	0.249652697478115	p__Ascomycota	357.1299	2.88	-1	1-OH-indole-3- carboxylic acid Gly derivative	other aromatic compounds	Benzene and substituted derivatives	1-OH-indole-3- carboxylic acid Gly derivative
rawcordf	162	2	31	0.528905374785187	1.76775314329985	0.00290862676677786	**	0.324818539283384	o__Xylariales	363.0765	12.99	-1				363.0765_12.99
rawcordf	163	2	9	-0.467380230861192	-1.4052442014271	0.0141685626570504	*	0.223639738054879	o__Capnodiales	225.9845	12.66	-1				225.9845_12.66
rawcordf	163	2	28	-0.471054229758762	-1.01845264517833	0.0134663073926796	*	0.281872136265039	o__Saccharomycetales	225.9845	12.66	-1				225.9845_12.66
rawcordf	163	2	31	0.612564966987476	1.83572526809551	0.000147759186710128	***	0.449082975487358	o__Xylariales	225.9845	12.66	-1				225.9845_12.66
rawcordf	164	2	28	0.424301399139175	1.2302986292424	0.0358626153239792	*	0.152030517740626	o__Saccharomycetales	340.0601	13.08	-1				340.0601_13.08
rawcordf	6	3	1	0.42972273838116	1.04735084709917	0.0319790089430781	*	0.188623123485077	c__Agaricomycetes	287.0559	12.86	1				287.0559_12.86
rawcordf	6	3	8	0.419540233844191	1.44935679070433	0.0404928599429265	*	0.186402569249747	f__Morosphaeriaceae	287.0559	12.86	1				287.0559_12.86
rawcordf	6	3	29	0.4615384666502	1.65507565520822	0.0161922981670532	*	0.222047110327371	g__Setophoma	287.0559	12.86	1				287.0559_12.86
rawcordf	8	3	1	-0.454816036972761	-1.4949500419793	0.0179796616439816	*	0.212562120799583	c__Agaricomycetes	204.0158	11.29	1		other compounds	Organonitrogen compounds	other compounds_204.0158_11.29
rawcordf	8	3	8	-0.44717681422131	-1.18336452006537	0.0213004189447958	*	0.166919965892862	f__Morosphaeriaceae	204.0158	11.29	1		other compounds	Organonitrogen compounds	other compounds_204.0158_11.29
rawcordf	8	3	22	-0.426198357109928	-1.10476963653342	0.0351007443371977	*	0.185778398793508	g__Melanoleuca	204.0158	11.29	1		other compounds	Organonitrogen compounds	other compounds_204.0158_11.29
rawcordf	8	3	23	-0.422754177925938	-1.28934063295426	0.0374905606750145	*	0.23561638079452	g__Monosporascus	204.0158	11.29	1		other compounds	Organonitrogen compounds	other compounds_204.0158_11.29
rawcordf	8	3	29	-0.477703876059092	-1.16019161306465	0.0113365433480006	*	0.246659917984151	g__Setophoma	204.0158	11.29	1		other compounds	Organonitrogen compounds	other compounds_204.0158_11.29
rawcordf	13	3	8	0.431881672802987	1.72018192724686	0.0300257124177767	*	0.203381938880001	f__Morosphaeriaceae	166.069	12.18	1	N,N-(Dimethyl)thiobenzamide	other compounds	Aralkylamines	N,N-(Dimethyl)thiobenzamide
rawcordf	18	3	1	0.448748770433733	1.53255861699956	0.020571066045602	*	0.235309147953278	c__Agaricomycetes	449.1098	12.86	1	Kaempferol-hexoside	flavonoid glycosides		Kaempferol-hexoside
rawcordf	18	3	8	0.425722401782735	1.42599993237533	0.035335290940394	*	0.17403694976583	f__Morosphaeriaceae	449.1098	12.86	1	Kaempferol-hexoside	flavonoid glycosides		Kaempferol-hexoside
rawcordf	18	3	29	0.438068856672701	1.398221119737	0.0260512538339147	*	0.224913790647443	g__Setophoma	449.1098	12.86	1	Kaempferol-hexoside	flavonoid glycosides		Kaempferol-hexoside
rawcordf	20	3	23	0.439468354154595	1.51271998618686	0.0257566659231812	*	0.196485850936192	g__Monosporascus	518.3259	16.79	1	1-18:3-lysoPC	Lipids and lipid-like molecules	Lysophosphatidylcholines	1-18:3-lysoPC
rawcordf	21	3	29	0.464102950862376	1.59123476276516	0.0153242129227423	*	0.22335103140211	g__Setophoma	287.056	13.51	1				287.056_13.51
rawcordf	24	3	30	0.47072906590769	1.1443874331073	0.0135202749917411	*	0.289393201345843	g__Tetracladium	423.1021	11.48	1				423.1021_11.48
rawcordf	26	3	33	-0.414175986473867	-0.73695163963922	0.046105813438408	*	0.193224564756835	o__Pleosporales	268.1052	9.72	1				268.1052_9.72
rawcordf	31	3	22	-0.413513609616703	-1.03060838644953	0.0465325858918075	*	0.195008483029881	g__Melanoleuca	471.1058	11.06	1	GSH + 3-methylsulfinylpropyl ITC	Peptides	Gamma-glutamyl peptides	GSH + 3-methylsulfinylpropyl ITC
rawcordf	31	3	29	-0.495838442988048	-1.60865169339729	0.00686646996985805	**	0.253413305637413	g__Setophoma	471.1058	11.06	1	GSH + 3-methylsulfinylpropyl ITC	Peptides	Gamma-glutamyl peptides	GSH + 3-methylsulfinylpropyl ITC
rawcordf	36	3	20	-0.414365762188411	-1.09335574857917	0.046105813438408	*	0.186610796318494	g__Fusarium	164.0206	12.71	1				164.0206_12.71
rawcordf	41	3	8	0.439277377410949	1.02421707536975	0.0257566659231812	*	0.195614791435706	f__Morosphaeriaceae	325.0474	12.98	1		other aromatic compounds	Benzenoids	other aromatic compounds_325.0474_12.98
rawcordf	41	3	29	0.433054374150679	1.17821683320508	0.029364712848756	*	0.174939163329208	g__Setophoma	325.0474	12.98	1		other aromatic compounds	Benzenoids	other aromatic compounds_325.0474_12.98
rawcordf	44	3	23	-0.463371915774758	-1.42883890664468	0.0154323946600516	*	0.215048683301855	g__Monosporascus	295.1299	11.82	1	Gamma-Glu-Phe	Peptides	Gamma-glutamyl amino acids	Gamma-Glu-Phe
rawcordf	44	3	29	-0.556699148666285	-2.11299638187658	0.00128102054961322	**	0.376485026375353	g__Setophoma	295.1299	11.82	1	Gamma-Glu-Phe	Peptides	Gamma-glutamyl amino acids	Gamma-Glu-Phe
rawcordf	50	3	8	0.413999179617793	1.19594896554119	0.0461878660292202	*	0.159359167475921	f__Morosphaeriaceae	272.5316	13.25	1				272.5316_13.25
rawcordf	50	3	29	0.504699651828333	1.51039859416093	0.00568677355438378	**	0.338049405681925	g__Setophoma	272.5316	13.25	1				272.5316_13.25
rawcordf	50	3	33	-0.43878707405661	-1.23994035696774	0.0257566659231812	*	0.192826730358146	o__Pleosporales	272.5316	13.25	1				272.5316_13.25
rawcordf	52	3	29	-0.438538565094816	-1.16751733003752	0.0257566659231812	*	0.192805917898397	g__Setophoma	314.1836	9.93	1				314.1836_9.93
rawcordf	55	3	29	-0.444319786939878	-1.18539037750103	0.0232518803557677	*	0.197750543297747	g__Setophoma	285.1683	9.54	1		Lipids and lipid-like molecules	Glycerolipids	Lipids and lipid-like molecules_285.1683_9.54
rawcordf	59	3	1	-0.456585082757914	-1.53209177500873	0.0172435710971282	*	0.245689893302368	c__Agaricomycetes	357.9849	12.13	1		other aromatic compounds	Benzenesulfonamides	other aromatic compounds_357.9849_12.13
rawcordf	59	3	8	-0.443855377141483	-1.55778368549059	0.0235089102922126	*	0.190761957211112	f__Morosphaeriaceae	357.9849	12.13	1		other aromatic compounds	Benzenesulfonamides	other aromatic compounds_357.9849_12.13
rawcordf	59	3	29	-0.474073505576181	-1.24322228944372	0.0122746095556082	*	0.227476683943461	g__Setophoma	357.9849	12.13	1		other aromatic compounds	Benzenesulfonamides	other aromatic compounds_357.9849_12.13
rawcordf	64	3	17	0.448085186068257	1.3422497012677	0.0208380863100638	*	0.247857950467397	g__Debaryomyces	455.1615	12.79	1		Amino acid derivatives and peptides	N-acyl-alpha amino acids and derivatives	Amino acid derivatives and peptides_455.1615_12.79
rawcordf	69	3	23	-0.416869872048177	-1.14352172384336	0.0433351694597489	*	0.20709929575508	g__Monosporascus	277.1058	12.32	1				277.1058_12.32
rawcordf	69	3	29	-0.551661768963798	-1.42239667787006	0.00136694095964666	**	0.346218052987258	g__Setophoma	277.1058	12.32	1				277.1058_12.32
rawcordf	69	3	30	-0.481385245278349	-1.77365728339592	0.0106455591907138	*	0.281019361000815	g__Tetracladium	277.1058	12.32	1				277.1058_12.32
rawcordf	69	3	33	0.46030746691966	1.20429379193247	0.0164649477444857	*	0.255504816537925	o__Pleosporales	277.1058	12.32	1				277.1058_12.32
rawcordf	73	3	31	-0.459386446886447	-1.15478826168389	0.0179796616439816	*	0.194122070527778	g__Thanatephorus	323.0964	13.08	1				323.0964_13.08
rawcordf	84	3	1	-0.426596755051613	-1.24008434779331	0.035089484591702	*	0.213261977312514	c__Agaricomycetes	423.1387	12.58	1	Cys Cys Pro Thr	Peptides	Peptides	Cys Cys Pro Thr
rawcordf	84	3	8	-0.491610659228884	-1.61075428469684	0.00775474955496497	**	0.294028059298928	f__Morosphaeriaceae	423.1387	12.58	1	Cys Cys Pro Thr	Peptides	Peptides	Cys Cys Pro Thr
rawcordf	84	3	22	-0.422360691219409	-1.10322727694467	0.0376904452264492	*	0.231143242994973	g__Melanoleuca	423.1387	12.58	1	Cys Cys Pro Thr	Peptides	Peptides	Cys Cys Pro Thr
rawcordf	84	3	23	-0.460202045231583	-1.90949095506196	0.0164649477444857	*	0.255614595414954	g__Monosporascus	423.1387	12.58	1	Cys Cys Pro Thr	Peptides	Peptides	Cys Cys Pro Thr
rawcordf	84	3	29	-0.480999190640127	-1.55679240219984	0.0106455591907138	*	0.257781255116784	g__Setophoma	423.1387	12.58	1	Cys Cys Pro Thr	Peptides	Peptides	Cys Cys Pro Thr
rawcordf	88	3	23	-0.426211493830769	-1.14725338471956	0.0351007443371977	*	0.218193340287949	g__Monosporascus	262.0753	12.32	1				262.0753_12.32
rawcordf	88	3	29	-0.4737426638385	-1.43992373506916	0.0123251163073485	*	0.2230034618111	g__Setophoma	262.0753	12.32	1				262.0753_12.32
rawcordf	90	3	22	-0.441745186618773	-0.818590216660028	0.0245571213136056	*	0.215519841516974	g__Melanoleuca	272.1729	10.84	1				272.1729_10.84
rawcordf	94	3	29	-0.470170703685827	-1.26382246552353	0.0137003866247964	*	0.261503497561179	g__Setophoma	247.1451	12.3	1	Indol-3-ylmethyl amino derivative	Amino acid derivatives and peptides	Alpha amino acids	Indol-3-ylmethyl amino derivative
rawcordf	96	3	22	-0.411796360407331	-0.949429404322661	0.0489192742827776	*	0.164992170656268	g__Melanoleuca	258.1573	9.67	1				258.1573_9.67
rawcordf	97	3	1	0.467710375202896	1.27769253743766	0.0141685626570504	*	0.235610931144838	c__Agaricomycetes	535.1106	13.49	1	Kaempferol derivate	flavonoid glycosides	Flavonoid-3-O-glycosides	Kaempferol derivate
rawcordf	97	3	29	0.450247402967942	1.67099812977681	0.0198459044374519	*	0.202853054494329	g__Setophoma	535.1106	13.49	1	Kaempferol derivate	flavonoid glycosides	Flavonoid-3-O-glycosides	Kaempferol derivate
rawcordf	98	3	33	-0.437939994763065	-1.25430155874307	0.0260512538339147	*	0.203464669415331	o__Pleosporales	307.0939	12.24	1		Amino acid derivatives and peptides	Alpha amino acids and derivatives	Amino acid derivatives and peptides_307.0939_12.24
rawcordf	102	3	8	0.43259642918055	1.28581093650447	0.0296950309027911	*	0.203253573966292	f__Morosphaeriaceae	325.5489	12.98	1				325.5489_12.98
rawcordf	102	3	10	0.426447019963766	1.396814339886	0.0351007443371977	*	0.167138774413041	f__Pezizaceae	325.5489	12.98	1				325.5489_12.98
rawcordf	102	3	29	0.452739683250486	1.3359718802473	0.0184938782162769	*	0.258765498811503	g__Setophoma	325.5489	12.98	1				325.5489_12.98
rawcordf	104	3	23	-0.452821982437182	-1.89486037296252	0.0184938782162769	*	0.227686972451106	g__Monosporascus	350.0369	10.54	1				350.0369_10.54
rawcordf	106	3	23	0.410756604852216	1.58367717337292	0.0498572792169073	*	0.184930918776001	g__Monosporascus	417.0133	11.23	1		Amino acid derivatives and peptides	Alpha hydroxy acids and derivatives	Amino acid derivatives and peptides_417.0133_11.23
rawcordf	111	3	1	0.433966158506034	1.32869539578563	0.0291069500947835	*	0.231138783613575	c__Agaricomycetes	539.0059	13.25	1				539.0059_13.25
rawcordf	111	3	29	0.469568405324122	1.59320477043979	0.0139073765128278	*	0.267914497767304	g__Setophoma	539.0059	13.25	1				539.0059_13.25
rawcordf	113	3	8	0.487750153432787	1.44956370819139	0.00870187084421334	**	0.228582720843139	f__Morosphaeriaceae	273.0328	13.25	1				273.0328_13.25
rawcordf	113	3	29	0.518117399581323	1.76075400453374	0.0038588887692747	**	0.317133181752626	g__Setophoma	273.0328	13.25	1				273.0328_13.25
rawcordf	117	3	29	-0.447639878107427	-1.01844112557023	0.0210610324296335	*	0.233018353160731	g__Setophoma	298.1156	10.61	1		Nucleotide derivatives	Purine nucleosides	Nucleotide derivatives_298.1156_10.61
rawcordf	118	3	23	-0.457668838832425	-1.79054185380083	0.0171238171269551	*	0.204532146487591	g__Monosporascus	393.1012	10.53	1				393.1012_10.53
rawcordf	131	3	8	0.43348445708335	1.41255747918374	0.0293330927161539	*	0.206817782035203	f__Morosphaeriaceae	388.0748	11.52	-1	pentyl GSL	Glucosinolates	Alkylglucosinolates	pentyl GSL
rawcordf	132	3	1	-0.532077345763977	NA	0.00275858054608679	**	0.341824581265314	c__Agaricomycetes	226.1081	12.68	-1				226.1081_12.68
rawcordf	132	3	8	-0.416194316838301	NA	0.0439713078774204	*	0.198667689962545	f__Morosphaeriaceae	226.1081	12.68	-1				226.1081_12.68
rawcordf	136	3	8	0.449100155357745	1.52142799054851	0.0204247348040311	*	0.199126356795726	f__Morosphaeriaceae	549.1256	13.13	-1	Kaempferol-dipentoside	flavonoid glycosides	Flavonoid-3-O-glycosides	Kaempferol-dipentoside
rawcordf	136	3	29	0.44596838393342	1.95811801684661	0.0221371982222611	*	0.231252422882077	g__Setophoma	549.1256	13.13	-1	Kaempferol-dipentoside	flavonoid glycosides	Flavonoid-3-O-glycosides	Kaempferol-dipentoside
rawcordf	139	3	29	0.481550602377356	2.05913639895174	0.0106455591907138	*	0.284300272032879	g__Setophoma	417.0833	13.42	-1				417.0833_13.42
rawcordf	145	3	14	0.425905577129062	1.12298054578333	0.0352989405320159	*	0.22556306126805	g__Brachyphoris	581.1192	12.23	-1				581.1192_12.23
rawcordf	155	3	29	-0.502593111225475	-1.1281570013065	0.00570624326541637	**	0.24287324033713	g__Setophoma	282.085	9.84	-1				282.085_9.84
rawcordf	159	3	34	-0.431905857827634	-1.06217055841409	0.0300257124177767	*	0.214938617068154	p__Ascomycota	357.1299	2.88	-1	1-OH-indole-3- carboxylic acid Gly derivative	other aromatic compounds	Benzene and substituted derivatives	1-OH-indole-3- carboxylic acid Gly derivative
rawcordf	163	3	23	0.488328699458644	1.47391189016777	0.00859173139660302	**	0.294765805579008	g__Monosporascus	225.9845	12.66	-1				225.9845_12.66
rawcordf	163	3	29	0.413431490491443	1.72182427842337	0.0465325858918075	*	0.203343321820305	g__Setophoma	225.9845	12.66	-1				225.9845_12.66
rawcordf	140	4	11	-0.410769477430851	-1.10121223833939	0.0498572792169073	*	0.175865432473265	Fisher_alpha	224.056	10.42	-1				224.056_10.42
rawcordf	141	4	5	-0.453937728937729	-1.22427269396288	0.0201090498303102	*	0.194070046262092	Shannon_H	208.0612	10.38	-1	formyl tyrosine	Amino acid derivatives and peptides		formyl tyrosine
rawcordf	141	4	7	-0.456438375011303	-1.23039364289722	0.0172435710971282	*	0.212394711981182	Brillouin	208.0612	10.38	-1	formyl tyrosine	Amino acid derivatives and peptides		formyl tyrosine
type_diversity	NA	4	1	NA	NA	0.0108238383838384	*	NA	PC4_1	NA	NA	NA	NA	NA	NA	NA
type_diversity	NA	4	2	NA	NA	0.0122746095556082	*	NA	PC4_2	NA	NA	NA	NA	NA	NA	NA
type_diversity	NA	4	3	NA	NA	0.0139857142857143	*	NA	PC4_3	NA	NA	NA	NA	NA	NA	NA
type_diversity	NA	4	4	NA	NA	0.0139857142857143	*	NA	PC4_4	NA	NA	NA	NA	NA	NA	NA
type_diversity	NA	4	5	NA	NA	0.0038588887692747	**	NA	PC4_5	NA	NA	NA	NA	NA	NA	NA
